# Supplementary material for: A neutral cyclic aluminium (I) trimer
Source: Nat Commun. 2026 Jan 30;17:1732. doi: 10.1038/s41467-026-68432-1 (PMC12913658; doi:10.1038/s41467-026-68432-1)
Supplement: Supplementary file 1 — Supplementary Information [file 41467_2026_68432_MOESM1_ESM.pdf]

Supplementary information for:

A neutral cyclic aluminium (I) trimer

Imogen Squire[ϕ], Matthew de Vere-Tucker[ϕ], Michelangelo Tritto[ϕ], Lygia Silva de Moraes, Tobias Krämer* and Clare Bakewell*

[ϕ]Authors contributed equally

Table of contents

1. **General Experimental Section** **S2**
2. **Synthetic procedures** **S3**
3. **Supplementary figures** **S14**
4. **X-ray crystallographic data** **S29**
5. **Computational analysis** **S45**
6. **Multinuclear NMR data** **S72**
7. **References** **S93**

1. General Experimental Section

All manipulations were carried out using standard Schlenk-line and glovebox techniques under an inert atmosphere. An MBraun Labmaster glovebox with an atmosphere of N_2 was employed, operating at < 0.1 ppm O_2 and < 0.1 ppm H_2O. Glassware was dried for at least 12 h at 125 °C prior to use. Toluene and hexane were dried over activated alumina from an SPS (solvent purification system) based upon the Grubbs design, pentane was distilled over CaH_2 and all solvents were stored over activated 3Å molecular sieves and degassed via the freeze-pump-thaw method prior to use. Additionally, hexane for synthesis of **2** was filtered onto K prior to use. Benzene-d_6 and cyclohexane-d_{12} were purchased dry and stored over activated 3Å molecular sieves. Ethene gas was purchased from CK isotopes. Dihydrogen gas was obtained from a PEAK Scientific hydrogen generator. NMR-scale reactions were conducted in J Young tap tubes and prepared in a glovebox. NMR tubes were heated using a DrySyn NMR tube heating block or a Julabo Corio CD-BC4 oil bath. Capillary internal standards of mesitylene in benzene-d_6 and cyclohexane-d_{12} were used to obtain NMR yields where appropriate. ^{1}H (tetramethylsilane; 0 ppm) and ^{13}C (tetramethylsilane; 0 ppm) NMR spectra were obtained on BRUKER 400 MHz, 700 MHz or 800 MHz (with cryoprobe) instruments; all chemical shift values are quoted in ppm. All attempts to obtain ^{27}Al NMR spectra were unsuccessful.[1]

Data was processed using MestReNova software. Elemental analysis was conducted by Orfhlaith McCullough at London Metropolitan University and has been obtained to the best of our abilities given the extremely air and moisture sensitive nature of the compounds. UV spectra were acquired using an Agilent Cary100 UV-Vis spectrometer. EPR spectra were obtained using a Magnettech ESR5000 at the PEPR facility (Imperial College London). Ligands[2,3], aluminium amidinate dihydride complexes[4], compound **II**[5] and $[(^{Mes}BDIMg)_2]$[6] were synthesised *via* literature procedures.

2. Synthetic procedures

Precursor synthesis

Compound 1 general synthesis (see figure S1)

Aluminium amidinate dihydride complex (1 equiv.) was dissolved in hexane (20 mL) and a solution of iodine (1 equiv.) in hexane was added dropwise with stirring until suspension remained light yellow. The mixture was then stirred for a further hour, during which time the colour disappeared. The solvent was removed *in vacuo* and the solid crystallised from toluene, giving the product as a colourless crystalline solid.

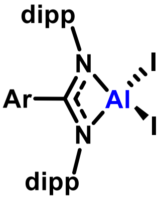

***1**$^{p\text{-}tol}$*

600 mg aluminium amidinate dihydride, 315.5 mg iodine, yield: 687.2 mg (75%). Isolated as powder, washed with hexane.

^{1}H NMR (400 MHz, benzene-d_6, 298 K): δ_H 0.89 (d, 12H, CH(CH_3)$_2$, $^3J_{HH}$ = 6.8 Hz), 1.38 (d, 12H, CH(CH_3)$_2$, $^3J_{HH}$ = 6.8 Hz), 1.58 (s, 3H, *p*-CH_3), 3.72 (hept, 4H, C*H*(CH$_3$)$_2$, $^3J_{HH}$ = 6.8 Hz), 6.32 (d, 2H, *p*-tol-*m-H*, $^3J_{HH}$ = 8.1 Hz), 6.95 – 7.04 (m, Ar*H*, 6H), 7.05 – 7.10 (m, Ar*H*, 2H). **^{13}C{^{1}H} NMR** (101 MHz, benzene-d_6, 298 K): δ_C 21.0 (*p*-CH$_3$), 23.1 (CH(*C*H$_3$)$_2$), 27.3 (CH(*C*H$_3$)$_2$), 29.1 (*C*H(CH$_3$)$_2$), 124.7 (Ar*C*), 127.5 (Ar*C*), 129.0 (Ar*C*), 131.0 (Ar*C*), 135.9 (C^{IV}), 143.3 (C^{IV}-*p*-CH$_3$), 144.6 (C^{IV}), 176.9 (N*C*(Ar)N). **^{27}Al NMR** (104 MHz, benzene-d_6, 298 K): not observed.

Elemental analysis calculated for $C_{32}H_{41}AlI_2N_4$: C, 52.33; H, 5.63; N, 3.81. Found: C, 52.98; H, 5.71; N, 3.74.

***1**$^{m\text{-}xyl}$*

591 mg aluminium amidinate dihydride, 293.2 mg iodine, yield: 542.4 mg (63%).

^{1}H NMR (400 MHz, benzene-d_6, 298 K): δ_H 0.92 (d, 12H, CH(CH_3)$_2$, $^3J_{HH}$ = 6.8 Hz), 1.38 (d, 12H, CH(CH_3)$_2$, $^3J_{HH}$ = 6.7 Hz), 1.70 (dd, 6H, *m*-Xyl-CH_3, $^4J_{HH}$ = 0.7 Hz), 3.72 (hept, 4H, C*H*(CH$_3$)$_2$, $^3J_{HH}$ = 6.8 Hz), 6.41 (dq, 1H, *m*-Xyl-*p-H*, $^3J_{HH}$ = 0.8, 1.6 Hz), 6.75 – 6.81 (qd, 2H, *m*-Xyl-*o-H*, $^3J_{HH}$ = 1.6, 0.8 Hz), 6.95 – 7.09 (m, 6H, Ar*H*). **^{13}C{^{1}H} NMR** (101 MHz, benzene-d_6, 298 K): δ_C 20.7 (*m*-Xyl-*C*H$_3$), 23.1 (CH(*C*H$_3$)$_2$), 27.4 (CH(*C*H$_3$)$_2$), 29.1 (*C*H(CH$_3$)$_2$), 124.5 (Ar*C*), 127.5 (*m*-Xyl-*C*), 128.9 (*m*-Xyl-*C*), 133.6 (*m*-Xyl-*C*), 135.8 (Ar*C*), 138.0 Ar*C*), 144.7 (Ar*C*), 177.5 (N*C*(Ar)N).

Elemental analysis calculated for $C_{33}H_{43}AlI_2N_2.0.1(C_7H_8)$: C 53.42, H 5.83, N 3.70. Found: C 53.79, H 6.05, N 3.43.

Compound 5

Route 1: Potassium (1 equiv.) and **1**$^{p\text{-tol}}$ (1 equiv.) were stirred vigorously in hexane (15 mL) for 8 days. The solvent was removed *in vacuo* and the product was extracted into toluene, then recrystallised at −30 °C. The product was isolated as a colourless crystalline solid.*

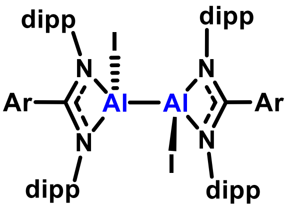

Route 2: To a J Young NMR tube charged with a solution of **1** (2 equiv.) in benzene-d_6 (0.6 mL) was added **II** (1 equiv.). The resultant solution was heated to 80 °C for 3 hours and allowed to cool slowly to room temperature. The solution was decanted, affording **5** as colourless crystals suitable for single-crystal X-ray diffraction.

*The low solubility of $\mathbf{5}^{\textbf{\textit{p}-tol}}$ in hexane, toluene and benzene makes isolating the product from the finely divided KI challenging, decreasing the isolated yield.

$5^{\text{p-}tol}$

Route 1: 5 mg potassium, 100 mg $\mathbf{1}^{\textbf{\textit{p}-tol}}$, yield 10.5 mg (13%).
Route 2: 97.5 mg $1^{p\text{-tol}}$, 29 mg II, yield 42.6 mg (68%).
^{1}H NMR (400 MHz, benzene-d_6, 298 K): δ_H 1.02 (d, 12H, CH(CH_3)$_2$, $^3J_{HH}$ = 6.8 Hz), 1.06 (d, 12H, CH(CH_3)$_2$, $^3J_{HH}$ = 6.8 Hz), 1.11 (d, 12H, CH(CH_3)$_2$, $^3J_{HH}$ = 6.8 Hz), 1.57 (s, 6H, *p*-CH_3), 1.59 (d, 12H, CH(CH_3)$_2$, $^3J_{HH}$ = 6.8 Hz), 4.00 (hept, 4H, C*H*(CH$_3$)$_2$, $^3J_{HH}$ = 6.8 Hz), 4.10 (hept, 4H, C*H*(CH$_3$)$_2$, $^3J_{HH}$ = 6.8 Hz), 6. (d, 4H, *p*-tol-*m-H*, $^3J_{\text{HH}}$ = 8.0 Hz), 6.96 (m, Ar*H,* 4H), 7.02 – 7.11 (m, Ar*H*, 12H). **^{13}C NMR** (101 MHz, benzene-d_6, 298 K): δ_C 20.9 (*p*-*C*H$_3$), 23.2 (CH(*C*H$_3$)$_2$), 23.9 (CH(*C*H$_3$)$_2$), 26.9 (CH(*C*H$_3$)$_2$), 27.5 (CH(*C*H$_3$)$_2$), 28.7 (*C*H(CH$_3$)$_2$), 28.8 (*C*H(CH$_3$)$_2$), 124.4 (Ar*C*), 124.6 (Ar*C*), 126.1 (Ar*C*), 126.9 (Ar*C*), 128.6 (Ar*C*), 130.8 (Ar*C*), 137.8 (C^{IV}), 141.9 (C^{IV}), 144.1 (C^{IV}), 145.3 (C^{IV}), 172.5 (C^{IV}-*p*-CH$_3$), 175.2 (N*C*(Ar)N).

$5^{\text{m-}xyl}$

Route 2: 10 mg $\mathbf{1}^{\textbf{\textit{m}-xyl}}$, 2.7 mg **II**, yield 5 mg (65%).
^{1}H NMR (400 MHz, benzene-d_6, 298 K): δ_H 1.04 (d, 12H, CH(CH_3)$_2$, $^3J_{\text{HH}}$ = 6.7 Hz), 1.11 (d, 12H, CH(CH_3)$_2$, $^3J_{\text{HH}}$ = 6.8 Hz), 1.12 (d, 12H, CH(CH_3)$_2$, $^3J_{\text{HH}}$ = 6.8 Hz), 1.60 (d, 12H, CH(CH_3)$_2$, $^3J_{\text{HH}}$ = 6.6 Hz), 1.71 (s, 12H *m*-Xyl-CH_3), 4.02 (hept, 4H, C*H*(CH$_3$)$_2$), 4.10 (hept, 4H, C*H*(CH$_3$)$_2$), 6.37 (s, 2H, *m*-Xyl-*p-H*), 6.85 (d, 4H, *m*-Xyl-*o-H*,$^4J_{\text{HH}}$ = 1.6 Hz), 6.93 (dd, Ar*H*, 4H), 7.00 – 7.08 (m, Ar*H*, 8H). **^{13}C{^{1}H} NMR** (101 MHz, benzene-d_6, 353 K) δ 20.6 (*m*-Xyl-*C*H$_3$), 23.2(CH(*C*H$_3$)$_2$), 23.8(CH(*C*H$_3$)$_2$), 27.1(CH(*C*H$_3$)$_2$), 27.7(CH(*C*H$_3$)$_2$), 28.7 (*C*H(CH$_3$)$_2$), 28.8 (*C*H(CH$_3$)$_2$), 124.4 (Ar*C*), 124.4 (Ar*C*), 126.8 (Ar*C*), 128.9 (Ar*C*), 132.6 (Ar*C*), 137.4 (Ar*C*), 138.0 (Ar*C*), 144.5 (Ar*C*), 145.7 (Ar*C*), 175.8 (N*C*(Ar)N.

Synthesis of compound $2^{\text{p-tol}}$ and $2^{\text{m-xyl}}$ (cyclotrialumanes)

$2^{\text{p-}tol}$

Route 1: Aluminium diiodide $\mathbf{1}^{\textbf{\textit{p}-tol}}$ (200 mg, 0.27 mmol) and potassium* (42 mg, 1.1 mmol) were stirred vigorously in hexane (15 mL) for 24 hours†, during which time the solution turned dark red-black (Figure S 2). The mixture was filtered and stored at −30 °C overnight, affording the product as a black crystalline solid (37.8 mg, 29 %).

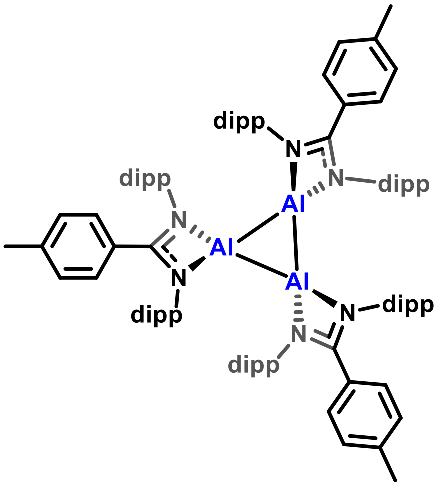

Further crops were obtained by concentrating and cooling the filtrate to −30 °C, although these are contaminated with a mixture of **5**$^{p\text{-tol}}$ and **A**. The yield of total crude material is generally around 60%, and several small crystalline crops of **2**$^{p\text{--tol}}$ can be obtained from this in varying yields. Doing the reactions on smaller scale (100 mg) leads to higher yields, likely due to the low solubility of **5**$^{p\text{-tol}}$ limiting reaction efficiency on larger scale. This could be surmounted by working at higher dilution.

*The potassium is divided into *ca.* 4 mg pieces before being added to the reaction vial. Vigorous stirring at room temperature in aliphatic solvents breaks these pieces up over a period of approximately 2 hours to afford a suspension of finely divided potassium. This is carried out *in situ* with **1** added to the vial.

†As the reduction is heterogeneous, the rate of reaction depends on the efficiency of stirring and consequently reaction times can vary.[7] Generally, the rate of reduction of **1**$^{m\text{-xyl}}$ is faster than **1**$^{p\text{-tol}}$ (reactions generally complete at 16 hours and 24 hours respectively from ^{1}H NMR of aliquots). However, the first reduction product (**5**$^{m\text{-xyl}/p\text{-tol}}$) is essentially insoluble in the reaction solvent so it is difficult to determine when this has been fully consumed by aliquot.

Route 2: Compound **II** (4.3 mg, 0.009 mmol) and compound **A** (9.4 mg, 0.009 mmol) were combined in cyclohexane-d_{12} (0.6 mL) and heated at 80 °C for 1 hour, at which point a mixture of all four species were observed in solution (Figure S 12). The resonances of **2**$^{p\text{-tol}}$ match those observed when synthesised independently *via* route 1, but it does not appear possible to push the equilibrium of the reaction far enough to allow isolation of the products.

^{1}H NMR (400 MHz, benzene-d_6, 298 K): δ_H 0.43 (d, 18H, CH(C*H*)$_3$, $^{3}J_{HH}$ = 6.8 Hz), 0.44 (d, 18H, CH(C*H*)$_3$, $^{3}J_{HH}$ = 6.8 Hz), 1.37 (d, 18H, CH(C*H*)$_3$, $^{3}J_{HH}$ = 6.8 Hz), 1.56 (d, 18H, CH(C*H*)$_3$, $^{3}J_{HH}$ = 6.8 Hz), 1.66 (s, 9H, *p*-C*H*$_3$), 3.33 (hept, 6H, C*H*(CH$_3$)$_2$, $^{3}J_{HH}$ = 6.8 Hz), 4.01 (hept, 6H, C*H*(CH$_3$)$_2$, $^{3}J_{HH}$ = 6.8 Hz), 6.42 (d, 6H, Ar*H*, $^{3}J_{HH}$ = 8.1 Hz), 6.98 – 7.07 (m, 12H, Ar*H*), 7.08 – 7.15 (m, 12H, Ar*H*). **^{13}C{^{1}H} NMR** (101 MHz, benzene-d_6, 298 K): δ_C 21.0 (*p*-*C*H$_3$), 23.1 (CH(*C*H$_3$)$_2$), 23.3 (CH(*C*H$_3$)$_2$), 24.0 (CH(*C*H$_3$)$_2$), 28.0 (CH(*C*H$_3$)$_2$), 28.6 (*C*H(CH$_3$)$_2$), 29.1 (*C*H(CH$_3$)$_2$), 123.4 (*C*H), 124.4 (*C*H), 125.4 (*C*H), 128.5 (*C*H), 131.4 (*C*H), 140.1 (C^{IV}), 140.7 (C^{IV}), 143.7 (C^{IV}), 144.7 (C^{IV}), 164.7 (N*C*(Ar)N). **^{27}Al NMR** (104 MHz, benzene-d_6, 298 K): not observed.

^{1}H NMR (400 MHz, cyclohexane-d_{12}, 298 K): δ_H 0.06 (d, 18H, CH(C*H*)$_3$, $^{3}J_{HH}$ = 6.8 Hz), 0.15 (d, 18H, CH(C*H*)$_3$, $^{3}J_{HH}$ = 6.8 Hz), 1.03 (d, 18H, CH(C*H*)$_3$, $^{3}J_{HH}$ = 6.8 Hz), 1.27 (d, 18H, CH(C*H*)$_3$, $^{3}J_{HH}$ = 6.8 Hz), 2.02 (s, 9H, *p*-C*H*$_3$), 3.00 (hept, 6H, C*H*(CH$_3$)$_2$, $^{3}J_{HH}$ = 6.8 Hz), 3.66 (hept, 6H, C*H*(CH$_3$)$_2$, $^{3}J_{HH}$ = 6.8 Hz), 6.59 (d, 6H, Ar*H*, $^{3}J_{HH}$ = 8.2 Hz), 6.73 – 6.77 (m, 6H, Ar*H*), 6.79-6.83 (m, 6H, Ar*H*), 6.87 – 6.94 (m, 12H, Ar*H*). **^{13}C{^{1}H} NMR** (101 MHz, cyclohexane-d_{12}, 298 K): δ_C 21.3 (*p*-*C*H$_3$), 22.9 (CH(*C*H$_3$)$_2$), 23.3 (CH(*C*H$_3$)$_2$), 24.1 (CH(*C*H$_3$)$_2$), 28.2 (CH(*C*H$_3$)$_2$), 28.8 (*C*H(CH$_3$)$_2$), 29.2 (*C*H(CH$_3$)$_2$), 123.5 (*C*H), 124.4 (*C*H), 125.4 (*C*H), 127.9 (*C*H), 128.5 (*C*H), 132.0 (*C*H), 139.9 (C^{IV}), 140.9 (C^{IV}), 143.8 (C^{IV}), 145.0 (C^{IV}), 164.7 (N*C*(Ar)N).

UV/Vis (cyclohexane): λ_{max} 434 nm and 311 nm.

$2^{m\text{-}xyl}$

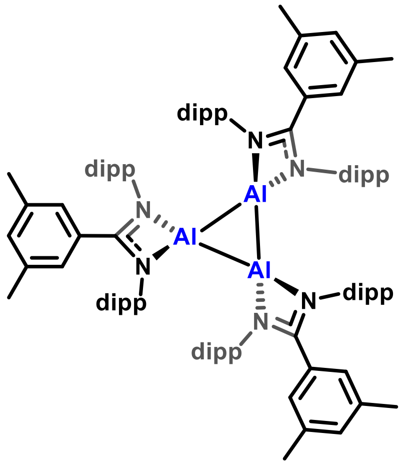

A vial charged with aluminium diiodide complex **1**$^{m\text{-}xyl}$ (1 equiv.), potassium (4 equiv.) and hexane (*ca*. 15 mL) was vigorously stirred at room temperature for 16 hours, affording a deep red-black solution. The mixture was allowed to settle, filtered, concentrated and stored at -40 °C overnight to afford **2**$^{m\text{--}xyl}$ as a black crystalline solid. Further crops were obtained by the same method to afford pure **2**$^{m\text{-}xyl}$ (54 mg, 82%).
The yield of total crude material is as high as 95%, although in all cases crystalline crops were used for onward reactivity.
^{1}H NMR (400 MHz, benzene-d_6, 298 K): δ_H 0.38 (d, CH(C*H*)$_3$, $^3J_{HH}$ = 6.7 Hz, 18H), 0.46 (d, CH(C*H*)$_3$, $^3J_{HH}$ = 6.8 Hz, 18H), 1.38 (d, CH(C*H*)$_3$, $^3J_{HH}$ = 6.8 Hz, 18H), 1.57 (d, CH(C*H*)$_3$, $^3J_{HH}$ = 6.6 Hz, 18H), 1.80 (s, *m*Xyl-*m*-C*H*$_3$, 18H), 3.33 (hept, C*H*(CH$_3$)$_2$, $^3J_{HH}$ = 6.3 Hz, 6H), 4.00 (hept, C*H*(CH$_3$)$_2$, $^3J_{HH}$ = 6.8 Hz, 6H), 6.44 (s, *m*Xyl-*p-H*, 3H), 6.79 (s, *m*Xyl-*o-H*, 6H), 6.98 (dd, Ar*H*, *J* = 2.2, 7.1 Hz, 6H), 7.06 – 7.13 (m, Ar*H*, 12H). **^{13}C{^{1}H} NMR** (101 MHz, benzene-d_6 298 K): δ_c 20.9 (*m*Xyl-*C*H$_3$), 23.1 (CH(*C*H$_3$)$_2$), 23.3 (CH(*C*H$_3$)$_2$), 24.3 (CH(*C*H$_3$)$_2$), 28.0 (CH(*C*H$_3$)$_2$), 28.6 (*C*H(CH$_3$)$_2$), 28.9 (*C*H(CH$_3$)$_2$), 123.2 (Ar*C*), 124.3 (Ar*C*), 125.4 (Ar*C*), 129.6 (Ar*C*), 130.5 (Ar*C*), 131.2 (Ar*C*), 137.0 (Ar*C*), 140.6 (Ar*C*), 143.8 (Ar*C*), 144.9 (Ar*C*), 164.8 (N*C*(Ar)N).

^{1}H NMR (400 MHz, cyclohexane-d_{12}, 298 K): δ_H 0.04 (d, CH(C*H*)$_3$, $^3J_{HH}$ = 6.7 Hz, 18H), 0.17 (d, CH(C*H*)$_3$, $^3J_{HH}$ = 6.7 Hz, 18H), 1.06 (d, CH(C*H*)$_3$, $^3J_{HH}$ = 6.8 Hz, 18H), 1.28 (d, CH(C*H*)$_3$, $^3J_{HH}$ = 6.6 Hz, 18H), 1.82 (s, *m*Xyl-*m*-C*H*$_3$, 18H), 3.01 (hept, C*H*(CH$_3$)$_2$, $^3J_{HH}$ = 6.9 Hz, 6H), 3.68 (hept, C*H*(CH$_3$)$_2$, $^3J_{HH}$ = 6.7 Hz, 6H), 6.49 (s, *m*Xyl-*o-H*, 6H), 6.56 (s, *m*Xyl-*p-H*, 3H), 6.79 – 6.93 (m, Ar*H*, 18H). **^{13}C{^{1}H} NMR** (101 MHz, cyclohexane-d_{12}): δ_C 20.9 (*m*-Xyl-*C*H$_3$), 22.9 (CH(*C*H$_3$)$_2$), 23.3 (CH(*C*H$_3$)$_2$), 23.5 (CH(*C*H$_3$)$_2$), 24.3 (CH(*C*H$_3$)$_2$), 28.3 (*C*H(CH$_3$)$_2$), 28.8 (*C*H(CH$_3$)$_2$), 29.1 (*C*H(CH$_3$)$_2$), 123.3 (Ar*C*), 124.3 (Ar*C*), 125.3 (Ar*C*), 130.1 (Ar*C*), 130.9 (Ar*C*), 131.0 (Ar*C*), 137.1 (Ar*C*), 140.9 (Ar*C*), 144.0 (Ar*C*), 145.1 (Ar*C*), 164.9 (N*C*(Ar)N).

Elemental analysis calculated for $C_{107}H_{153}Al_3N_6O_2Si_3$ (**2**$^{m\text{-}xyl}$.($Si_3O_2C_8H_{24}$)): C 74.69, H 8.96, N 4.88. Found: C 74.12, H 8.57, N 4.68.

UV/Vis (cyclohexane): λ_{max} 478 nm and 315 nm.

Note: Several aliphatic solvents have been tested for the reaction, and generally the reactions have been found to proceed similarly in pentane, hexane and cyclohexane for **1**$^{p\text{-}tol}$, however **1**$^{m\text{-}xyl}$ appears more sensitive to the reaction solvent, with lower yields obtained from attempted reductions in pentane, and notably larger quantities of the apparent decomposition product [(^{Dip}Am^{mXyl}AlH)$_2$] (dihydrodialane) (as verified *via* comparison with independently synthesised **8**$^{m\text{-}xyl}$).

Reactivity studies with $2^{p\text{-}tol}$ and $2^{m\text{-}xyl}$

Compound 3

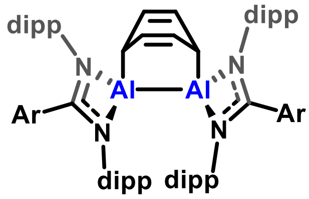

Route 1: A J Young NMR tube charged with a dark red-black solution of **2** (1 equiv.) in excess benzene (0.6 mL) was heated to 80 °C for three hours. The resultant red-orange solution was dried *in vacuo* and redissolved in benzene-d_6. ^{1}H NMR spectroscopic analysis showed full conversion of **2** to **3**.
Route 2: A vial containing $\mathbf{1}^{p\text{-tol}}$ (1 equiv.) and KC_8 (2 equiv.)* in benzene (15 mL) was stirred vigorously for 5 days. The solution was allowed to settle and filtered before concentrating to an oil and adding pentane, to afford the product as a red crystalline solid.
*The route 2 reaction was also attempted with K as the reducing agent, but although the starting material was consumed, very little of **3** was formed. This may be in part due to difficulties finely dividing K in benzene, which results in a reduced surface area and precludes effective reduction.

$\mathit{3}^{p\text{-}tol}$

Route 1: 34.8 mg $\mathbf{2}^{p\text{-tol}}$, 0.6 mL benzene, yield: 29.5 mg (78%).
Route 2: 200 mg $\mathbf{1}^{p\text{-tol}}$, 74 mg KC_8, yield 103 mg (72%).

Characterisation has been previously reported.[8,9]

$\mathit{3}^{m\text{-}xyl}$

Route 1: 26.4 mg $\mathbf{2}^{m\text{-xyl}}$, 0.6 mL benzene, yield: 21.4 mg (75%).
^{1}H NMR (400 MHz, benzene-d_6, 298 K): δ_H 0.42 (d, 6H, CH(CH_3)$_2$, $^3J_{HH}$ = 6.8 Hz), 0.54 (d, 6H, CH(CH_3)$_2$, $^3J_{HH}$ = 6.7 Hz), 0.84 (d, 6H, CH(CH_3)$_2$, $^3J_{HH}$ = 6.7 Hz), 1.24 (d, 6H, CH(CH_3)$_2$, $^3J_{HH}$ = 6.6 Hz), 1.35 (d, 6H, CH(CH_3)$_2$, $^3J_{HH}$ = 6.9 Hz), 1.40 (d, 6H, CH(CH_3)$_2$, $^3J_{HH}$ = 6.8 Hz), 1.50 (d, 12H, CH(CH_3)$_2$, $^3J_{HH}$ = 6.8 Hz), 1.74 (s, 12H, *m*-Xyl-CH_3), 2.75 (tt, 2H, AlC*H*, $^3J_{HH}$ = 6.3 Hz, $^4J_{HH}$ = 1.3 Hz), 3.23 (v. dhept, 4H, C*H*(CH$_3$)$_2$, $^3J_{HH}$ = 6.8 Hz), 3.94 (hept, 2H, C*H*(CH$_3$)$_2$, $^3J_{HH}$ = 5.9 Hz), 4.05 (hept, 2H, C*H*(CH$_3$)$_2$, $^3J_{HH}$ = 6.8 Hz), 5.40 (dd, 2H, AlCH-C*H*=C*H*, $^4J_{HH}$ = 6.3 Hz, $^3J_{HH}$ = 8.4 Hz), 6.09 (dd, 2H, AlCH-C*H*=C*H*, $^4J_{HH}$ = 6.4 Hz, $^3J_{HH}$ = 8.3 Hz), 6.40 (s, 2H, *m*-Xyl-*p*-*H*), 6.73 (s, 4H, *m*-Xyl-*o*-*H*), 6.83 (m, 2H, Ar*H*), 6.96 – 7.13 (m, 8H, Ar*H*), 7.18 – 7.25 (m, 4H, Ar*H*). **^{13}C{^{1}H} NMR** (101 MHz, Benzene-d_6, 298 K): δ_C 20.8 (*m*-Xyl-*C*H$_3$), 22.7 (CH(*C*H$_3$)$_2$), 22.7 (CH(*C*H$_3$)$_2$), 23.2 (CH(*C*H$_3$)$_2$), 23.8 (CH(*C*H$_3$)$_2$), 24.1 (CH(*C*H$_3$)$_2$), 24.7 (CH(*C*H$_3$)$_2$), 25.5 (CH(*C*H$_3$)$_2$), 26.0 (CH(*C*H$_3$)$_2$), 26.5 (CH(*C*H$_3$)$_2$), 28.1 (*C*H(CH$_3$)$_2$), 28.4 (*C*H(CH$_3$)$_2$), 28.6 (*C*H(CH$_3$)$_2$), 28.7 (*C*H(CH$_3$)$_2$), 38.6 (Al*C*H), 119.7 (AlCH*C*H), 123.3 (Ar*C*), 123.7 (Ar*C*), 124.3 (Ar*C*), 124.3 (Ar*C*), 125.8 (Ar*C*), 126.2 (AlCH*C*H), 126.6 (Ar*C*), 128.3 (Ar*C*), 128.6 (*m*-Xyl-*o*-*C*H), 129.5 (Ar*C*), 131.8 (*m*-Xyl-*p*-*C*H), 137.2 (*m*-Xyl-*m*-*C*), 139.3 (Ar*C*), 139.4 (Ar*C*), 143.4 (Ar*C*), 143.7 (Ar*C*), 144.5 (Ar*C*), 145.3 (Ar*C*), 172.5 (N*C*(*m*-Xyl)N).

Compound 4$^{p\text{-}tol}$

A vial containing **1$^{p\text{-}tol}$** (200 mg, 1 equiv.) and KC_8 (74 mg, 2 equiv.) in toluene (15 mL) was stirred vigorously for 6 days. The solution was allowed to settle and filtered before concentrating to approximately 5 mL and storing at -40 °C to afford the product as a red crystalline solid (85 mg, 44%)
Characterisation has been previously reported.[9]

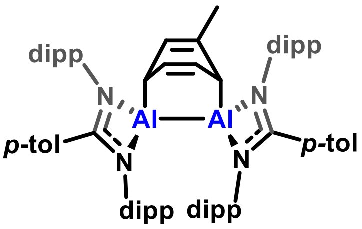

Compound 6

To a J Young NMR tube charged with a solution of **2** (1 equiv.) in cyclohexane-d_{12} (0.6 mL) was added *bis*(trimethylsilyl)acetylene (3 equiv.). This was heated to 80 °C for 6 hours, at which point complete conversion was confirmed *via* ^{1}H NMR spectroscopic analysis. **6** could be isolated following the removal of solvent *in vacuo* and subsequent extraction into pentane.

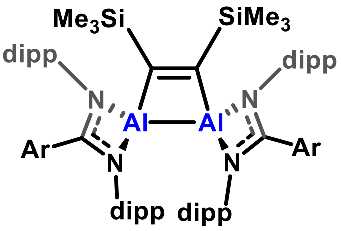

6$^{p\text{-}tol}$

8.9 mg **2$^{p\text{-}tol}$**, 3.2 mg bis(trimethylsilyl)acetylene, yield: 5.8 mg (55%).
^{1}H NMR (400 MHz, benzene-d_6, <u>298 K</u>): δ_H 0.39 (s, 18H, Si(CH_3)$_3$), 1.32 (d, 12H, CH(CH)$_3$, $^3J_{HH}$ = 6.7 Hz), 1.67 (s, 6H, *p*-CH_3), 3.48 (br s, 8H, CH(CH$_3$)$_2$), 6.38 (d, 4H, ArH, $^3J_{HH}$ = 8.1 Hz), 6.91 (d, 4H, ArH, $^3J_{HH}$ = 8.1 Hz), 7.08 (m, 12H, ArH). Some resonances corresponding to diisopropyl groups are missing, but spectrum is broad suggesting restricted rotation. **^{13}C{^{1}H} NMR** (101 MHz, benzene-d_6, 298 K): δ_C 3.0 (Si(CH$_3$)$_3$), 21.0 (*p*-CH$_3$), 22.8 (CH(CH$_3$)$_2$), 23.1 (CH(CH$_3$)$_2$), 24.3 (CH(CH$_3$)$_2$), 28.7 (CH(CH$_3$)$_2$), 28.7 (CH(CH$_3$)$_2$), 124.2 (CH), 124.4 (CH), 125.7 (CH), 127.7 (CH), 128.8 (CH), 131.6 (CH), 139.7 (C^{IV}), 140.8 (C^{IV}), 143.2 (C^{IV}), 171.2 (NC(Ar)N), 243.4 (AlCSiMe$_3$).

6$^{m\text{-}xyl}$

7.2 mg **2$^{m\text{-}xyl}$**, 2.5 mg bis(trimethylsilyl)acetylene, yield: 5.5 mg (65%).
^{1}H NMR (400 MHz, benzene-d_6, <u>298 K</u>): δ_H 0.40 (s, 18H, Si(CH_3)$_3$), 1.32 (d, 12H, CH(CH_3)$_2$, $^3J_{HH}$ = 6.7 Hz), 1.77 (s, 12H, *m*Xyl-*m*-CH_3), 3.34 – 3.65 (br. m, 8H, CH(CH$_3$)$_2$), 6.37 – 6.49 (s, 2H, *m*Xyl-*p-H*), 6.65 (S, 4H, *m*Xyl-*o-H*), 7.06 (m, 12H, ArH). Some resonances correspond to diisopropyl groups are missing, but broadened spectrum indicates restricted rotation. **^{1}H NMR** (400 MHz, benzene-d_6, <u>353 K</u>): δ_H 0.35 (s, 18H, Si(CH_3)$_3$), 0.71 (d, 12H, CH(CH_3)$_2$, $^3J_{HH}$ = 6.7 Hz), 0.82 (br. d, 12H, CH(CH_3)$_2$, $^3J_{HH}$ = 6.5 Hz), 1.05 (d, CH(CH_3)$_2$, 12H, $^3J_{HH}$ = 6.7 Hz), 1.29 (d, CH(CH_3)$_2$, 12H, $^3J_{HH}$ = 6.7 Hz), 1.79 (s, 12H, *m*-Xyl-CH_3), 3.34 – 3.61 (m, 8H, CH(CH$_3$)$_2$), 6.50 (s, 2H, *m*-Xyl-*p-H*), 6.62 (s, 4H, *m*-Xyl-*o-H*), 7.02 – 7.07 (m, 10H, ArH). **^{13}C{^{1}H} NMR** (101 MHz, benzene-d_6, 298 K): δ_C 3.0 (Si(CH$_3$)$_3$), 20.9 (*m*-Xyl-*m*-CH$_3$), 22.7 (CH(CH$_3$)$_2$), 22.8 (CH(CH$_3$)$_2$), 24.2 (CH(CH$_3$)$_2$), 28.6 (CH(CH$_3$)$_2$), 28.8 (CH(CH$_3$)$_2$), 124.1 (ArC), 125.6 (ArC), 129.7 (ArC), 130.3 (ArC), 131.7 (ArC), 137.4 (ArC), 139.6 (ArC), 143.2 (ArC), 171.4 (NC(Ar)N), 230.6 (AlCSiMe$_3$).

Elemental analysis calculated for $C_{74}H_{104}Al_2N_4Si_2$ (**6$^{m\text{-}xyl}$**.2.5(toluene)): C 79.06 H 8.99 N 4.03. Found: C 79.23, H 9.53, N 4.57

Compound $7^{p\text{-}tol}$

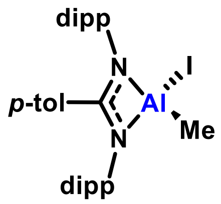

Route 1: To an J Young NMR tube containing a solution of $\mathbf{2}^{\boldsymbol{p}\textbf{-tol}}$ (37.5 mg, 1 equiv.) in benzene-d_6 (0.6 mL) was added methyl iodide (11.1 mg, 4.8 µL, 3 equiv.) and a colour change to very light brown was observed over the course of two minutes. The product was isolated as a white solid by removal of solvent *in vacuo* and washing with pentane (34.2 mg, 70 %).
Route 2: To a flask charged with a toluene solution of pro-ligand (1.0 g, 2.2 mmol, 1 equiv.) at room temperature was added a 2M solution of trimethylaluminium in toluene (1.3 mL, 2.6 mmol, 1.2 equiv) and stirred at room temperature overnight. The solvent was removed *in vacuo* and the product was washed with hexane to yield $^{p\text{-tol}}AmAlMe_2$ as a white powder (546 mg, 49%).[10] To a J Young NMR tube containing a solution of $^{p\text{-tol}}AmAlMe_2$ (50 mg, 1 equiv.) in benzene-d_6 (0.6 mL) was added iodine (25 mg, 1 equiv.). The product was dried *in vacuo* to remove the solvent and methyl iodide side-product and washed with pentane to obtain a white solid (52 mg, 85%).

^{1}H NMR (400 MHz, benzene-d_6, 298 K): δ_H 0.23 (s, 3H, AlCH_3), 0.91 (d, 6H, CH(CH_3)$_2$, $^3J_{HH}$ = 6.8 Hz), 0.95 (d, 6H, CH(CH_3)$_2$, $^3J_{HH}$ = 6.8 Hz), 1.18 (d, 6H, CH(CH_3)$_2$, $^3J_{HH}$ = 6.8 Hz), 1.46 (d, 6H, CH(CH_3)$_2$, $^3J_{HH}$ = 6.8 Hz), 1.61 (s, 9H, *p*-CH_3), 3.45 (hept, 2H, C*H*(CH$_3$)$_2$, $^3J_{HH}$ = 6.8 Hz), 3.87 (hept, 2H, C*H*(CH$_3$)$_2$, $^3J_{HH}$ = 6.8 Hz), 6.35 (d, 2H, Ar*H*, $^3J_{HH}$ = 8.2 Hz), 6.95 – 7.11 (m, 8H, Ar*H*). **^{13}C{^{1}H} NMR** (101 MHz, benzene-d_6, 298 K): δ_C 21.0 (*p*-*C*H$_3$), 23.0 (CH(*C*H$_3$)$_2$), 23.1 (CH(*C*H$_3$)$_2$), 25.7 (CH(*C*H$_3$)$_2$), 27.3 (CH(*C*H$_3$)$_2$), 28.9 (*C*H(CH$_3$)$_2$), 29.0 (*C*H(CH$_3$)$_2$), 124.0 (Ar*C*), 124.8 (Ar*C*), 125.5 (C^{IV}), 126.9 (Ar*C*), 128.9 (Ar*C*), 130.8 (C^{IV}), 137.1 (C^{IV}), 142.3 (C^{IV}), 143.7 (C^{IV}), 144.8 (C^{IV}), 175.6 (N*C*(Ar)N).

Compound 8/A

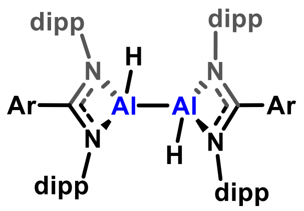

Route 1: To a J Young NMR tube charged with a degassed solution of **2** (1 equiv.) in benzene-d_6 (0.6 mL) was added an excess of H_2 gas (approx. 1 atm.). A rapid decolouration is observed. The product may be isolated *via* extraction into pentane to afford compound **8**.
Route 2: To a flask charged with aluminium dihydride (1 equiv.) and [(^{Mes}BDIMg)$_2$] (0.55 equiv.) was added toluene. The resultant reaction was stirred overnight at room temperature, filtered from toluene and extracted into benzene to afford **8** as a colourless solid.

A

Route 1: 17 mg $\mathbf{2}^{\boldsymbol{p}\textbf{-tol}}$, yield: 11.0 mg (65%). Characterisation has been previously reported.[6]

$8^{m\text{-}xyl}$

Route 1: 12 mg $\mathbf{2}^{\boldsymbol{m}\textbf{-xyl}}$, yield: 5.4 mg (45%).
Route 2: 100 mg aluminium dihydride, 79.2 mg [(^{Mes}BDIMg)$_2$], yield: 73.6 mg (74%).

^{1}H NMR (400 MHz, benzene-d_6, 298 K): δ_H 1.09 (d, 12H, CH(CH_3)$_2$, $^3J_{HH}$ = 6.8 Hz), 1.14 (d, 12H, CH(CH_3)$_2$, $^3J_{HH}$ = 6.9 Hz), 1.19 (d, 12H, CH(CH_3)$_2$, $^3J_{HH}$ = 6.8 Hz), 1.42 (d, 12H, CH(CH_3)$_2$, $^3J_{HH}$ = 6.8 Hz), 1.77 (s, 12H, *m*-Xyl-CH_3), 3.88 (v. dh, 8H, C*H*(CH$_3$)$_2$, $^3J_{HH}$ = 6.7 Hz), 5.68 (s, 2H, Al*H*), 6.39 (s, 2H, *m*-Xyl-*p-H*), 6.87 (s, 4H, *m*-Xyl-*o-H*), 6.98 – 7.03 (m, 12H, Ar*H*). **^{13}C{^{1}H} NMR** (101 MHz, benzene-d_6, 298 K): δ_C 20.8 (*m*-Xyl-*C*H$_3$), 21.4 (CH(*C*H$_3$)$_2$), 23.1 (CH(*C*H$_3$)$_2$), 23.1 (CH(*C*H$_3$)$_2$), 25.8 (CH(*C*H$_3$)$_2$), 26.0 (CH(*C*H$_3$)$_2$), 28.8 (*C*H(CH$_3$)$_2$), 28.9 (*C*H(CH$_3$)$_2$), 123.6 (Ar*C*), 123.8 (Ar*C*), 125.7 (Ar*C*), 126.0 (Ar*C*), 128.6 (Ar*C*), 129.3 (Ar*C*), 131.7 (Ar*C*), 137.1 (Ar*C*), 139.3 (Ar*C*), 144.0 (Ar*C*), 144.3 (Ar*C*), 171.8 (N*C*(Ar)N).

Compound 9

To a J Young NMR tube charged with a frozen degassed solution of **2** (1 equiv.) in cyclohexane (0.6 mL) was added ethene gas (1 atm.). The solution was allowed to thaw, instantaneously affording a red-orange solution. The solvent was immediately removed *in vacuo* and the red-orange solid redissolved in cyclohexane-d_{12} or benzene-d_6 to afford a red-orange solution of **9**$^{p\text{-tol}}$ or **9**$^{m\text{-xyl}}$, which was stable at room temperature. Crystals were afforded *via* the storage of concentrated pentane, hexane or heptane solutions at room temperature for several days.

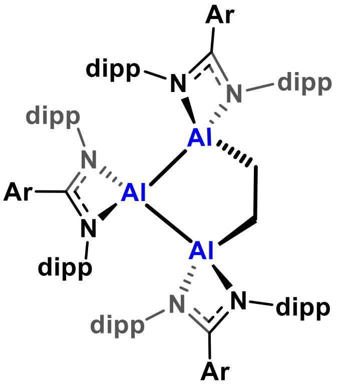

***9*$^{p\text{-}tol}$**

47 mg **2**$^{p\text{-tol}}$, 95% NMR conversion versus a mesitylene internal standard.
^{1}H NMR (700 MHz, benzene-d_6, 298 K): δ_H 0.29 (d, 6H, CH(CH_3)$_2$, $^3J_{HH}$ = 6.8 Hz), 0.32 (br, 2H, AlCH_2), 0.44 (d, 6H, CH(CH_3)$_2$, $^3J_{HH}$ = 6.8 Hz), 0.48 (d, 6H, CH(CH_3)$_2$, $^3J_{HH}$ = 6.8 Hz), 0.63 (d, 6H, CH(CH_3)$_2$, $^3J_{HH}$ = 6.8 Hz), 0.77 (d, 6H, CH(CH_3)$_2$, $^3J_{HH}$ = 6.8 Hz), 1.21 (d, 6H, CH(CH_3)$_2$, $^3J_{HH}$ = 6.8 Hz), 1.32 (d, 6H, CH(CH_3)$_2$, $^3J_{HH}$ = 6.8 Hz), 1.33 (d, 6H, CH(CH_3)$_2$, $^3J_{HH}$ = 6.8 Hz), 1.38 (br, 2H, AlCH_2), 1.45 (d, 6H, CH(CH_3)$_2$, $^3J_{HH}$ = 6.8 Hz), 1.52 (d, 6H, CH(CH_3)$_2$, $^3J_{HH}$ = 6.8 Hz), 1.62 – 1.67 (9H, *p*-CH_3 and CH(CH_3)$_2$), 3.21 (hept, 2H, C*H*(CH$_3$)$_2$, $^3J_{HH}$ = 6.8 Hz), 3.32 (hept, 2H, C*H*(CH$_3$)$_2$, $^3J_{HH}$ = 6.8 Hz), 3.36 (hept, 2H, C*H*(CH$_3$)$_2$, $^3J_{HH}$ = 6.8 Hz), 3.94 (hept, 2H, C*H*(CH$_3$)$_2$, $^3J_{HH}$ = 6.8 Hz), 4.05 (hept, 2H, C*H*(CH$_3$)$_2$, $^3J_{HH}$ = 6.8 Hz), 4.14 (hept, 2H, C*H*(CH$_3$)$_2$, $^3J_{HH}$ = 6.8 Hz), 6.37 (d, 4H, Ar*H*, $^3J_{HH}$ = 8.3 Hz), 6.40 (d, 2H, Ar*H*, $^3J_{HH}$ = 8.0 Hz), 6.97 (dd, J = 11.0, 7.9 Hz, 8H), 7.01 – 7.12 (m, 8H), 7.13 – 7.26 (m, 8H). **^{13}C{^{1}H} NMR** (176 MHz, benzene-d_6, 298 K): δ_C 8.8 (Al*C*H$_2$), 20.9 (*p*-*C*H$_3$), 22.7 (CH(*C*H$_3$)$_2$), 23.4 (CH(*C*H$_3$)$_2$), 23.7 (CH(*C*H$_3$)$_2$), 23.8 (CH(*C*H$_3$)$_2$), 24.0 (CH(*C*H$_3$)$_2$), 24.2 (CH(*C*H$_3$)$_2$), 24.5 (CH(*C*H$_3$)$_2$), 25.9 (CH(*C*H$_3$)$_2$), 26.3 (CH(*C*H$_3$)$_2$), 26.4 (CH(*C*H$_3$)$_2$), 27.1 (CH(*C*H$_3$)$_2$), 28.2 (*C*H(CH$_3$)$_2$), 28.5 (*C*H(CH$_3$)$_2$), 28.5 (*C*H(CH$_3$)$_2$), 28.6 (*C*H(CH$_3$)$_2$), 28.7 (*C*H(CH$_3$)$_2$), 28.8 (*C*H(CH$_3$)$_2$), 123.2 (*C*H), 123.8 (*C*H), 124.0 (*C*H), 124.4 (*C*H), 124.6 (*C*H), 124.7 (*C*H), 125.2 (*C*H), 125.8 (*C*H), 125.9 (*C*H), 127.6 (*C*H), 128.4 (*C*H), 128.5 (*C*H), 130.9 (*C*H), 140.1 (C^{IV}), 140.4 (C^{IV}), 140.6 (C^{IV}), 140.8 (C^{IV}), 143.0 (C^{IV}), 144.0 (C^{IV}), 144.2 (C^{IV}), 144.4 (C^{IV}), 145.3 (C^{IV}), 145.5 (C^{IV}), 167.2 (N*C*(Ar)N), 170.4 (N*C*(Ar)N).

***9*$^{m\text{-}xyl}$**

20 mg **2**$^{m\text{-xyl}}$, 95% NMR conversion versus a mesitylene internal standard.

^{1}H NMR (<u>400</u> MHz, benzene-d_6, 298 K) **:** δ_H 0.28 (d, 6H, CH(CH_3)$_2$, $^3J_{HH}$ = 6.8 Hz), 0.31 (br. t, 2H, AlCH_2, $^3J_{HH}$ = 13.3 Hz), 0.49 (d, 6H, CH(CH_3)$_2$, $^3J_{HH}$ = 6.6 Hz), 0.51 (d, 6H, CH(CH_3)$_2$, $^3J_{HH}$ = 6.8 Hz), 0.61 (d, 6H, CH(CH_3)$_2$, $^3J_{HH}$ = 6.8 Hz), 0.76 (d, 6H, CH(CH_3)$_2$, $^3J_{HH}$ = 6.8 Hz), 1.20 (d, 6H, CH(CH_3)$_2$, J = 6.9 Hz), 1.32 (d, 6H, CH(CH_3)$_2$, $^3J_{HH}$ = 6.9 Hz), 1.33 (d, 6H, CH(CH_3)$_2$, $^3J_{HH}$ = 7.0 Hz), 1.40 (d, 6H, CH(CH_3)$_2$, $^3J_{HH}$ = 6.7 Hz), 1.41 (t, 2H, AlCH_2), 1.44 (d, 6H, CH(CH_3)$_2$, $^3J_{HH}$ = 6.8 Hz), 1.52 (d, 6H, CH(CH_3)$_2$, $^3J_{HH}$ = 6.5 Hz), 1.63 (d, 6H, CH(CH_3)$_2$, $^3J_{HH}$ = 6.7 Hz), 1.76 (s, 12H, *m*-Xyl-CH_3), 1.78 (s, 6H, *m*-Xyl-CH_3), 3.20 (hept, 2H, C*H*(CH$_3$)$_2$, $^3J_{HH}$ = 6.6 Hz), 3.32 (hept, 2H, C*H*(CH$_3$)$_2$, $^3J_{HH}$ = 6.6 Hz), 3.39 (hept, 2H, C*H*(CH$_3$)$_2$, $^3J_{HH}$ = 7.0 Hz), 3.92 (hept, 2H, C*H*(CH$_3$)$_2$, $^3J_{HH}$ = 6.8 Hz), 4.03 (hept, 2H, C*H*(CH$_3$)$_2$, $^3J_{HH}$ = 6.8 Hz), 4.14 (hept, 2H, C*H*(CH$_3$)$_2$, $^3J_{HH}$ = 6.8 Hz), 6.40 (dq, 4H, *m*-Xyl-C*H*, $^3J_{HH}$ = 0.8, 1.6 Hz), 6.70 (s, 4H, *m*-Xyl-C*H*), 6.74 (s, 2H, *m*-Xyl-C*H*), 6.91 – 7.24 (m, 18H, Ar*H*).

^{1}H NMR (<u>800</u> MHz, benzene-d_6, 298 K): δ_H 0.28 (d, 6H, CH(CH_3)$_2$, $^3J_{HH}$ = 6.9 Hz), 0.30 – 0.34 (m, 2H, AlCH_2), 0.49 (d, 6H, CH(CH_3)$_2$, $^3J_{HH}$ = 6.8 Hz), 0.51 (d, 6H, CH(CH_3)$_2$, $^3J_{HH}$ = 6.9 Hz), 0.61 (d, 6H, CH(CH_3)$_2$, $^3J_{HH}$ = 6.7 Hz), 0.76 (d, 6H, CH(CH_3)$_2$, $^3J_{HH}$ = 6.8 Hz), 1.20 (d, 6H, CH(CH_3)$_2$, $^3J_{HH}$ = 6.9 Hz), 1.32 (d, 6H, CH(CH_3)$_2$, $^3J_{HH}$ = 6.8 Hz), 1.33 (d, 6H, CH(CH_3)$_2$, $^3J_{HH}$ = 7.1 Hz), 1.35 – 1.38 (m, 2H, AlCH_2), 1.40 (d, 6H, CH(CH_3)$_2$, $^3J_{HH}$ = 6.9 Hz), 1.44 (d, CH(CH_3)$_2$, 6H, $^3J_{HH}$ = 6.6 Hz), 1.52 (d, CH(CH_3)$_2$, 6H, $^3J_{HH}$ = 6.6 Hz), 1.63 (d, 6H, CH(CH_3)$_2$, $^3J_{HH}$ = 6.7 Hz), 1.76 (s, 12H, *m*-Xyl-CH_3), 1.78 (s, 6H, *m*-Xyl-CH_3), 3.19 (hept, 2H, C*H*(CH$_3$)$_2$, $^3J_{HH}$ = 6.9 Hz), 3.32 (hept, 2H, C*H*(CH$_3$)$_2$, $^3J_{HH}$ = 7.2 Hz), 3.38 (hept, 2H, C*H*(CH$_3$)$_2$, $^3J_{HH}$ = 6.8 Hz), 3.92 (hept, 2H, C*H*(CH$_3$)$_2$, $^3J_{HH}$ = 6.7 Hz), 4.03 (hept, 2H, C*H*(CH$_3$)$_2$, $^3J_{HH}$ = 6.8 Hz), 4.14 (hept, 2H, C*H*(CH$_3$)$_2$, $^3J_{HH}$ = 6.9 Hz), 6.40 (s, 4H, *m*-Xyl-C*H*), 6.70 (s, 4H, *m*-Xyl-C*H*), 6.74 (s, 2H, *m*-Xyl-C*H*), 6.78 – 6.79 (m, 1H, *m*-Xyl-C*H*), 6.81 – 7.15 (m, 16H, Ar*H*), 7.20 (d, 2H, Ar*H*, $^3J_{HH}$ = 7.7 Hz).

^{13}C{^{1}H} NMR (201 MHz, benzene-d_6, 298 K): δ_C 8.6 (AlCH$_2$), 20.8 (*m*-Xyl-*C*H$_3$), 20.9 (*m*-Xyl-*C*H$_3$), 22.7 (CH(*C*H$_3$)$_2$), 23.4 (CH(*C*H$_3$)$_2$), 23.6 (CH(*C*H$_3$)$_2$), 23.6 (CH(*C*H$_3$)$_2$), 23.9 (CH(*C*H$_3$)$_2$), 24.3 (CH(*C*H$_3$)$_2$), 24.5 (CH(*C*H$_3$)$_2$), 26.1 (CH(*C*H$_3$)$_2$), 26.4 (CH(*C*H$_3$)$_2$), 27.1 (CH(*C*H$_3$)$_2$), 28.1 (*C*H(CH$_3$)$_2$), 28.5 (*C*H(CH$_3$)$_2$), 28.5 (*C*H(CH$_3$)$_2$), 28.7 (*C*H(CH$_3$)$_2$), 28.8 (*C*H(CH$_3$)$_2$), 123.0 (Ar*C*), 123.6 (Ar*C*), 123.8 (Ar*C*), 124.3 (Ar*C*), 124.4 (Ar*C*), 124.6 (Ar*C*), 125.1 (Ar*C*), 125.7 (Ar*C*), 125.9 (Ar*C*), 129.0 (Ar*C*), 130.2 (Ar*C*), 131.2 (Ar*C*), 137.0 (Ar*C*), 137.0 (Ar*C*), 140.2 (Ar*C*), 140.5 (Ar*C*), 140.7 (Ar*C*), 143.1 (Ar*C*), 144.1 (Ar*C*), 144.3 (Ar*C*), 144.5 (Ar*C*), 145.4 (Ar*C*), 145.5 (Ar*C*), 170.8 (N*C*(Ar)N).

Synthesis of compounds 10, 11 and 12

To a J Young NMR tube charged with a frozen degassed solution of **2** (1 equiv.) in cyclohexane-d_{12} (0.6 mL) was added ethene gas (1 bar gauge). The frozen solution was allowed to thaw, affording a red-orange solution, which over the course of approximately 10 hours lost intensity, eventually affording a colourless solution. Product distribution could be worked out *via* ^{1}H NMR spectroscopy versus a mesitylene internal standard.

For assignment of **10$^{p\text{-tol}}$** and **10$^{m\text{-xyl}}$** in both cases, due to the number of resonances and overlapping crosspeaks assignment of ^{13}C resonances is tentative; unfortunately it proved impossible to completely separate **10** from **11** and **12**, nevertheless acquiring ^{1}H spectra on an 800 MHz spectrometer allowed for ^{1}H NMR assignment (Figure S 103).

As above, for assignment of **12$^{p\text{-tol}}$** and **12$^{m\text{-xyl}}$** due to the number of resonances and overlapping crosspeaks assignment of aromatic and all ^{13}C resonances is not possible, but tentative assignment of ^{1}H resonances is possible using an 800 MHz spectrometer (Figure S 109).

10 $^{p\text{-}tol}$

19 mg **2** $^{p\text{-}tol}$, 45% conversion by ^{1}H NMR spectroscopy.

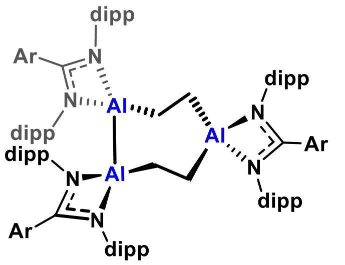

^{1}H NMR (700 MHz, benzene-d_6, 298 K): δ_H 0.27 (d, 6H, CH(CH_3)$_2$, $^3J_{HH}$ = 6.8 Hz), 0.48 (d, 6H, CH(CH_3)$_2$, $^3J_{HH}$ = 6.8 Hz), 0.63 (br, AlCH_2, 4H), 0.69 (d, 6H, CH(CH_3)$_2$, $^3J_{HH}$ = 6.8 Hz), 0.94 (br, AlCH_2, 2H), 0.98 – 1.04 (m, 6H, CH(CH_3)$_2$), 1.11 (br, CH(CH_3)$_2$, 2H), 1.18 (d, 6H, CH(CH_3)$_2$, $^3J_{HH}$ = 6.8 Hz), 1.23 (d, 6H, CH(CH_3)$_2$, $^3J_{HH}$ = 6.8 Hz), 1.24 – 1.29 (m, 6H, CH(CH_3)$_2$), 1.31 (m, 6H, CH(CH_3)$_2$), 1.37 (d, 12H, CH(CH_3)$_2$, $^3J_{HH}$ = 6.8 Hz), 1.45 (d, 6H, CH(CH_3)$_2$, $^3J_{HH}$ = 6.8 Hz), 1.47 (d, 6H, CH(CH_3)$_2$, $^3J_{HH}$ = 6.8 Hz), 1.62 (s, 9H, *p*-CH_3), 3.20 (hept, 2H, C*H*(CH$_3$)$_2$, $^3J_{\mathrm{HH}}$ = 6.8 Hz), 3.32 (hept, 2H, C*H*(CH$_3$)$_2$, $^3J_{\mathrm{HH}}$ = 6.8 Hz), 3.46 (hept, 2H, C*H*(CH$_3$)$_2$, $^3J_{\mathrm{HH}}$ = 6.8 Hz), 3.80 (hept, 2H, C*H*(CH$_3$)$_2$, $^3J_{\mathrm{HH}}$ = 6.8 Hz), 3.99 (hept, 2H, C*H*(CH$_3$)$_2$, $^3J_{\mathrm{HH}}$ = 6.8 Hz), 4.24 (hept, 2H, C*H*(CH$_3$)$_2$, $^3J_{\mathrm{HH}}$ = 6.8 Hz), 6.23 – 6.49 (m, 6H, Ar*H*), 6.88 – 7.14 (m, 20H, Ar*H*), 7.21 – 7.35 (m, 4H, Ar*H*). **^{13}C{^{1}H} NMR** (176 MHz, benzene-d_6, 298 K): δ_C -1.7 (Al*C*H$_2$), 8.1 (Al*C*H$_2$), 20.9 (*p*-*C*H$_3$), 22.8 (CH(*C*H$_3$)$_2$), 22.9 (CH(*C*H$_3$)$_2$), 23.1 (CH(*C*H$_3$)$_2$), 23.2 (CH(*C*H$_3$)$_2$), 23.3 (CH(*C*H$_3$)$_2$), 23.8 (CH(*C*H$_3$)$_2$), 23.9 (CH(*C*H$_3$)$_2$), 25.0 (CH(*C*H$_3$)$_2$), 25.5 (CH(*C*H$_3$)$_2$), 25.7 (CH(*C*H$_3$)$_2$), 26.0 (CH(*C*H$_3$)$_2$), 26.2 (CH(*C*H$_3$)$_2$), 26.9 (CH(*C*H$_3$)$_2$), 27.5 (*C*H(CH$_3$)$_2$), 28.3 (*C*H(CH$_3$)$_2$), 28.5 (*C*H(CH$_3$)$_2$), 28.7 (*C*H(CH$_3$)$_2$), 28.8 (*C*H(CH$_3$)$_2$), 29.0 (*C*H(CH$_3$)$_2$), 123.1 (*C*H), 123.4 (*C*H), 124.0 (*C*H), 124.4 (*C*H), 125.1 (*C*H), 125.2 (*C*H), 125.6 (*C*H), 125.9 (*C*H), 126.2 (*C*H), 126.3 (*C*H), 130.3 (*C*H), 130.5 (*C*H), 137.8 (*C*H), 138.4 (*C*H), 140.1 (C^{IV}), 140.2 (C^{IV}), 140.4 (C^{IV}), 143.3 (C^{IV}), 143.6 (C^{IV}), 143.7 (C^{IV}), 143.9 (C^{IV}), 144.3 (C^{IV}), 145.3 (C^{IV}), 146.1 (C^{IV}), 170.8 (N*C*(Ar)N), 172.2 (N*C*(Ar)N).

10 $^{m\text{-}xyl}$

20 mg **2** $^{m\text{-}xyl}$, 42% conversion by ^{1}H NMR spectroscopy.

^{1}H NMR (800 MHz, benzene-d_6, 298 K): δ_H 0.23 (d, 6H, CH(CH_3)$_2$, $^3J_{HH}$ = 6.8 Hz), 0.57 (d, 6H, CH(CH_3)$_2$, $^3J_{HH}$ = 6.8 Hz), 0.59 (d, 2H, AlCH_2, $^3J_{HH}$ = 14.2 Hz), 0.65 (d, 2H, AlCH_2, $^3J_{HH}$ = 12.8 Hz), 0.73 (d, 6H, CH(CH_3)$_2$, $^3J_{HH}$ = 6.8 Hz), 0.85 (d, 2H, AlCH_2, $^3J_{HH}$ = 13.4 Hz), 1.00 (d, 6H, CH(CH_3)$_2$, $^3J_{HH}$ = 6.8 Hz), 1.13 – 1.17 (m, 2H, AlCH_2), 1.20 (d, 6H, CH(CH_3)$_2$, $^3J_{HH}$ = 6.9 Hz), 1.22 (d, 6H, CH(CH_3)$_2$, $^3J_{HH}$ = 6.8 Hz), 1.23 (d, 6H, CH(CH_3)$_2$, $^3J_{HH}$ = 6.8 Hz), 1.39 (v. dd, 12H, CH(CH_3)$_2$, $^3J_{HH}$ = 6.6, 6.6 Hz), 1.42 (d, 6H, CH(CH_3)$_2$, $^3J_{HH}$ = 6.7 Hz), 1.46 (d, 6H, CH(CH_3)$_2$, $^3J_{HH}$ = 6.8 Hz), 1.75 (s, 6H, *m*-Xyl-CH_3), 1.78 (s, 12H, *m*-Xyl-CH_3), 3.16 (hept, 2H, C*H*(CH$_3$)$_2$, $^3J_{HH}$ = 6.8 Hz), 3.38 (hept, 2H, C*H*(CH$_3$)$_2$, $^3J_{HH}$ = 6.8 Hz), 3.48 (hept, 2H, C*H*(CH$_3$)$_2$, $^3J_{HH}$ = 6.6 Hz), 3.80 (hept, 2H, C*H*(CH$_3$)$_2$, $^3J_{HH}$ = 6.5 Hz), 3.96 (hept, 2H, C*H*(CH$_3$)$_2$, $^3J_{HH}$ = 6.8 Hz), 4.24 (hept, 2H, C*H*(CH$_3$)$_2$, $^3J_{HH}$ = 6.7 Hz), 6.39 (s, 1H, *m*-Xyl-*p-H*), 6.42 (s, 2H, *m*-Xyl-*p-H*), 6.71 (s, 4H, *m*-Xyl-*o-H*), 6.74 (a, 2H, *m*-Xyl-*o-H*), 6.91 (dd, 2H, Ar*H*, $^3J_{HH}$ = 1.6, 7.4 Hz), 6.94 (dd, 2H, Ar*H*, $^3J_{HH}$ = 1.6, 7.6 Hz), 6.98 (d, 1H, Ar*H*, $^3J_{HH}$ = 7.5 Hz), 7.03 (t, 4H, Ar*H*, $^3J_{HH}$ = 7.6 Hz), 7.06 (dd, 2H, Ar*H*, $^3J_{HH}$ = 1.7, 7.6 Hz), 7.08 (dd, 2H, Ar*H*, $^3J_{HH}$ = 1.5, 7.8 Hz), 7.14 (dd, 2H, Ar*H*, $^3J_{HH}$ = 1.5, 7.8 Hz), 7.20 (t, 2H, Ar*H*, $^3J_{HH}$ = 7.7 Hz), 7.26 (dd, 2H, Ar*H*, $^3J_{HH}$ = 1.6, 7.9 Hz). **^{13}C{^{1}H} NMR** (201 MHz, benzene-d_6): δ_C -1.9 (Al*C*H$_2$), 7.9 (Al*C*H$_2$), 20.8 (*m*-Xyl-*C*H$_3$), 20.8 (*m*-Xyl-*C*H$_3$), 22.7 (CH(*C*H$_3$)$_2$), 22.9 (CH(*C*H$_3$)$_2$), 23.1 (CH(*C*H$_3$)$_2$), 23.2 (CH(*C*H$_3$)$_2$), 23.4 (CH(*C*H$_3$)$_2$), 23.7 (CH(*C*H$_3$)$_2$), 24.1 (CH(*C*H$_3$)$_2$), 25.1 (CH(*C*H$_3$)$_2$), 25.3 (CH(*C*H$_3$)$_2$), 25.9 (CH(*C*H$_3$)$_2$), 26.0 (CH(*C*H$_3$)$_2$), 26.2 (CH(*C*H$_3$)$_2$), 28.3 (*C*H(CH$_3$)$_2$), 28.4 (*C*H(CH$_3$)$_2$), 28.5 (*C*H(CH$_3$)$_2$), 28.7 (*C*H(CH$_3$)$_2$), 28.8 (*C*H(CH$_3$)$_2$), 29.0 (*C*H(CH$_3$)$_2$), 123.0 (Ar*C*), 123.2 (Ar*C*), 123.8 (Ar*C*), 123.8 (Ar*C*), 123.9 (Ar*C*), 124.3 (Ar*C*), 124.4 (Ar*C*), 125.0 (Ar*C*), 125.1 (Ar*C*), 125.6 (Ar*C*), 125.7 (Ar*C*), 126.0 (Ar*C*), 128.9 (Ar*C*), 129.3 (Ar*C*), 130.4 (Ar*C*), 131.2 (Ar*C*), 139.2 (Ar*C*), 139.9 (Ar*C*), 140.4 (Ar*C*), 143.3 (Ar*C*), 143.7 (Ar*C*), 143.8 (Ar*C*), 144.0 (Ar*C*), 144.2 (Ar*C*), 144.5 (Ar*C*), 146.1 (Ar*C*), 171.2 (N*C*(Ar)N), 172.8 (N*C*(Ar)N).

11 **p-*tol***

27% conversion *via* ^{1}H NMR spectroscopy

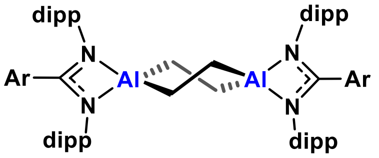

^{1}H NMR (400 MHz, benzene-d_6, 298 K): δ_H 1.01 (d, 24H, CH(C*H*$_3$)$_2$, $^3J_{HH}$ = 6.8 Hz), 1.20 (s, 8H, AlC*H*$_2$), (d, 24H, CH(C*H*$_3$)$_2$, $^3J_{HH}$ = 6.7 Hz), 1.62 (s, 6H, *p*-C*H*$_3$), 3.71 (hept, 4H, C*H*(CH$_3$)$_2$, $^3J_{HH}$ = 6.8 Hz), 6.35 – 6.41 (m, 4H, Ar*H*), 6.97 – 7.08 (m, 32H, Ar*H*). **^{13}C{^{1}H} NMR** (101 MHz, benzene-d_6, 298 K): δ_C 2.6 (Al*C*H$_2$), 20.9 (*p*-*C*H$_3$), 23.1 (CH(*C*H$_3$), 25.7 (CH(*C*H$_3$), 28.8 (*C*H(CH$_3$), 124.0 (*C*H), 125.9 (*C*H), 130.5 (*C*H), 139.2 (*C*H), 144.0 (C^{IV}), 173.1 (N*C*(Ar)N).

11 **m-*xyl***

32% conversion *via* ^{1}H NMR spectroscopy (RT)

^{1}H NMR (400 MHz, benzene-d_6, 298 K): δ_H 1.04 (d, 24H, CH(C*H*$_3$)$_2$, $^3J_{HH}$ = 6.9 Hz), 1.21 (s, 8H, AlC*H*$_2$), 1.31 (d, 24H, CH(C*H*$_3$)$_2$, $^3J_{HH}$ = 6.7 Hz), 1.76 (s, 12H, *m*-Xyl-C*H*$_3$), 3.72 (hept, 8H, C*H*(CH$_3$)$_2$, $^3J_{HH}$ = 6.8 Hz), 6.40 (m, 4H, Ar*H*), 6.68 – 6.75 (m, 2H, Ar*H*), 6.99 (s, 24H, Ar*H*). **^{13}C{^{1}H} NMR** (101 MHz, benzene-d_6, 298 K): δ_C 2.5 (Al*C*H$_2$), 14.3 (*m*-Xyl-*C*H$_3$), 20.8 (CH(*C*H$_3$)$_2$), 23.1 (CH(*C*H$_3$)$_2$), 25.9 (*C*H(CH$_3$)$_2$), 28.8 (CH(*C*H$_3$)$_2$), 123.8 (Ar*C*), 125.9 (Ar*C*), 137.1 (Ar*C*), 139.1 (Ar*C*), 144.2 (Ar*C*), 173.7 (N*C*(Ar)N).

12 **p-*tol***

Proposed:

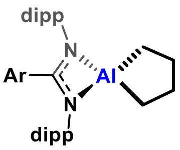

28% conversion *via* ^{1}H NMR spectroscopy (RT)

^{1}H NMR (400 MHz, benzene-d_6, 298 K): δ_H 0.55 (br, 4H, Al-(C*H*$_2$-CH$_2$)$_2$), 0.87 (d, 12H, CH(C*H*$_3$)$_2$, $^3J_{HH}$ = 6.8 Hz), 1.29 (d, 12H, CH(C*H*$_3$)$_2$, $^3J_{HH}$ = 6.8 Hz), 1.62 (s, 9H, *p*-C*H*$_3$), 2.06 (br, 4H, Al-(CH$_2$-C*H*$_2$)$_2$), 3.70 (hept, 4H, C*H*(CH$_3$)$_2$, $^3J_{HH}$ = 6.8 Hz).

12 **m-*xyl***

26% conversion *via* ^{1}H NMR spectroscopy (RT)

^{1}H NMR (800 MHz, Benzene-d_6, 298 K) δ 0.55 (t, 4H, AlC*H*$_2$CH$_2$, $^3J_{HH}$ = 6.9 Hz), 1.04 (d, 12H, CH(C*H*$_3$)$_2$, $^3J_{HH}$ = 7.0 Hz), 1.29 (d, 12H, CH(C*H*$_3$)$_2$, $^3J_{HH}$ = 6.9 Hz), 1.78 (s, 6H, *m*-Xyl-C*H*$_3$), 2.05 (p, 4H, AlCH$_2$C*H*$_2$, $^3J_{HH}$ = 3.8 Hz), 3.65 (hept, 4H, C*H*(CH$_3$)$_2$, $^3J_{HH}$ = 6.9 Hz).

3. Supplementary figures

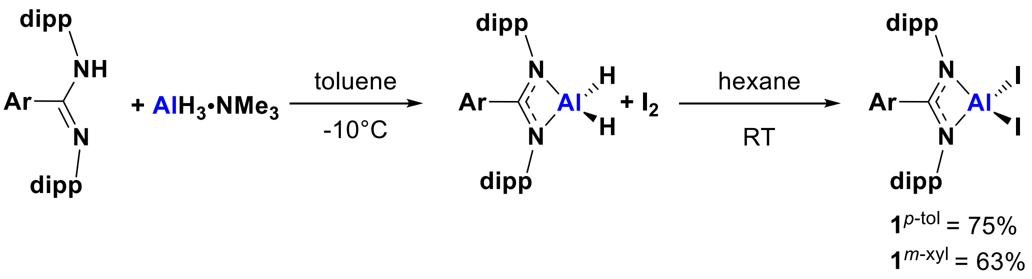

*Figure S 1: Reaction scheme for the formation of complexes **1** from amidine pro-ligands.*

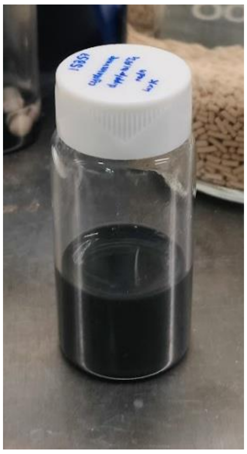

Figure S 2: Colour of reaction mixture for route 1 synthesis of $\mathbf{2}^{p\text{-}tol}$.

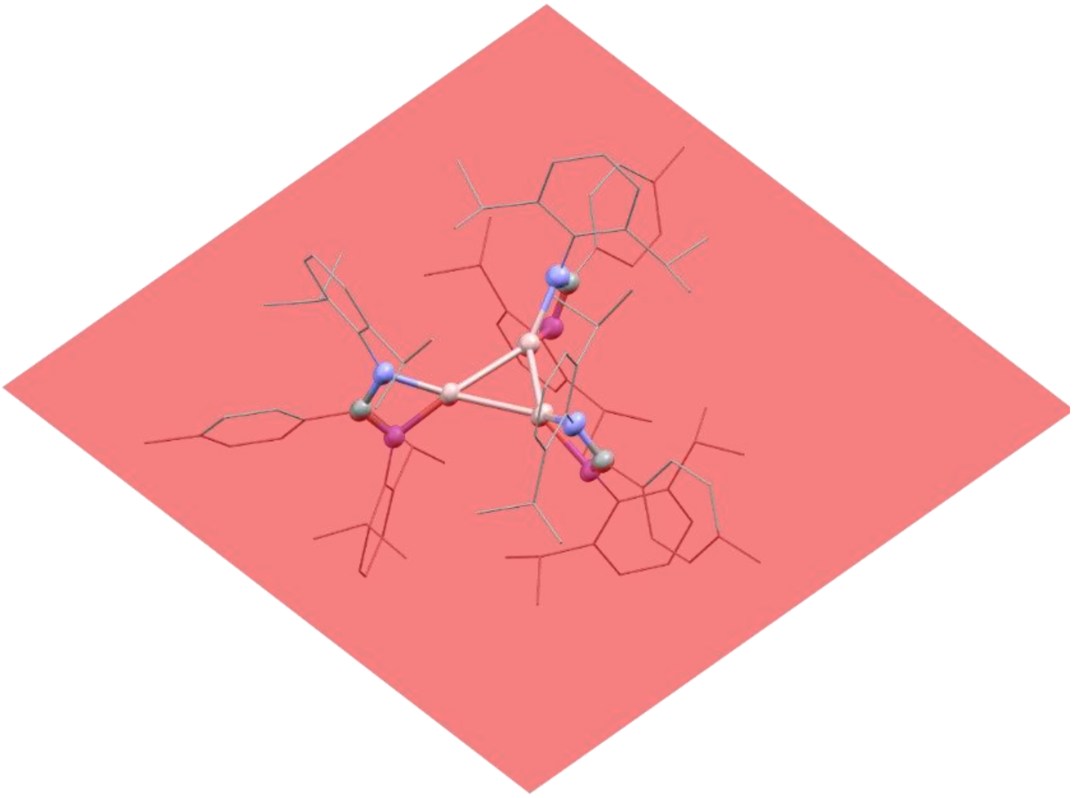

Figure S 3: Plane through Al_3 core of $\mathbf{2}^{p\text{-}tol}$ approximately bisecting the NCN backbone of the ligand.

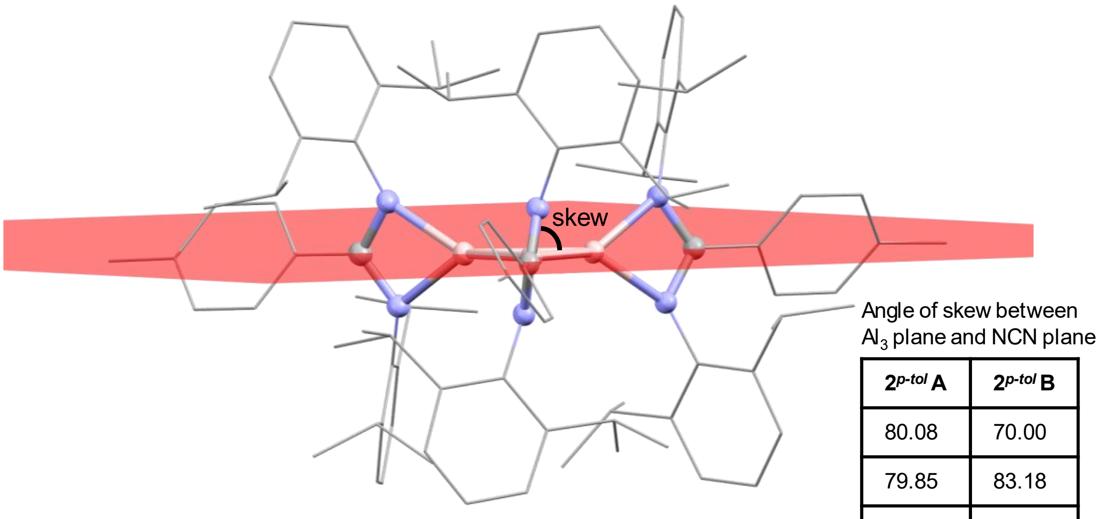

$2^{p\text{-}tol}$ A	$2^{p\text{-}tol}$ B
80.08	70.00
79.85	83.18
84.69	77.20

Figure S 4: Angle of skew between Al_3 and NCN planes, forming concentric pinwheel structure.

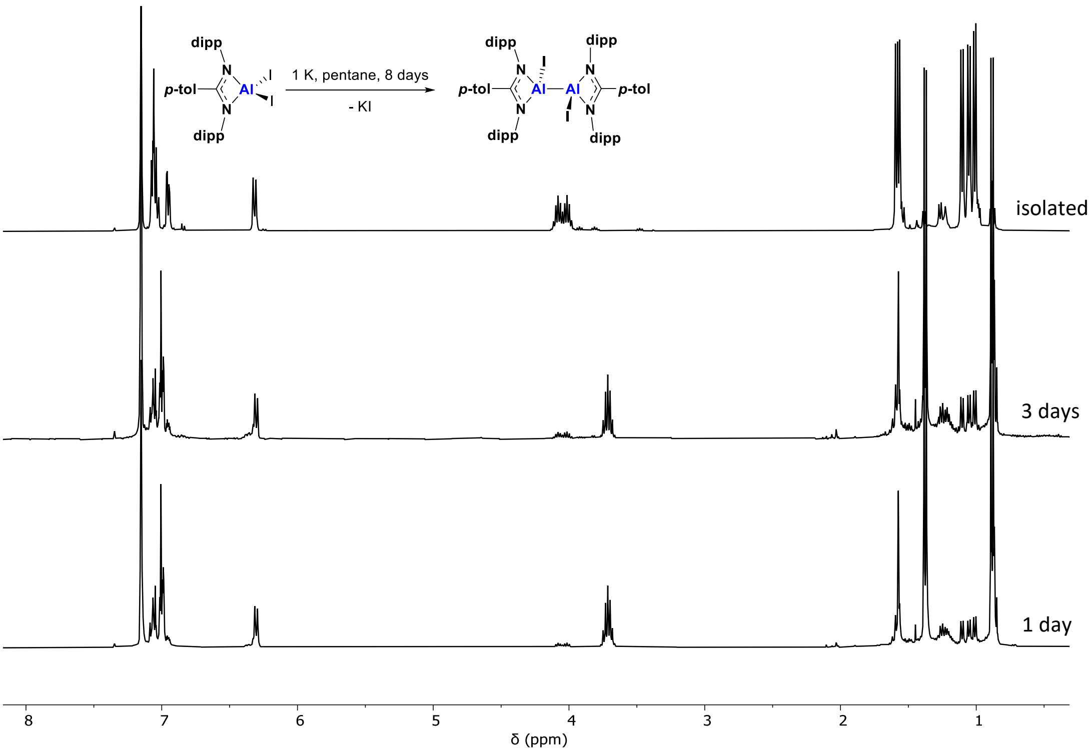

Figure S 5: ^{1}H NMR (400 MHz, 298 K, benzene-d_6) spectra showing reaction progress for reducing ***1****$^{p\text{-}tol}$ in pentane to make the diiododialane* ***5****$^{p\text{-}tol}$.*

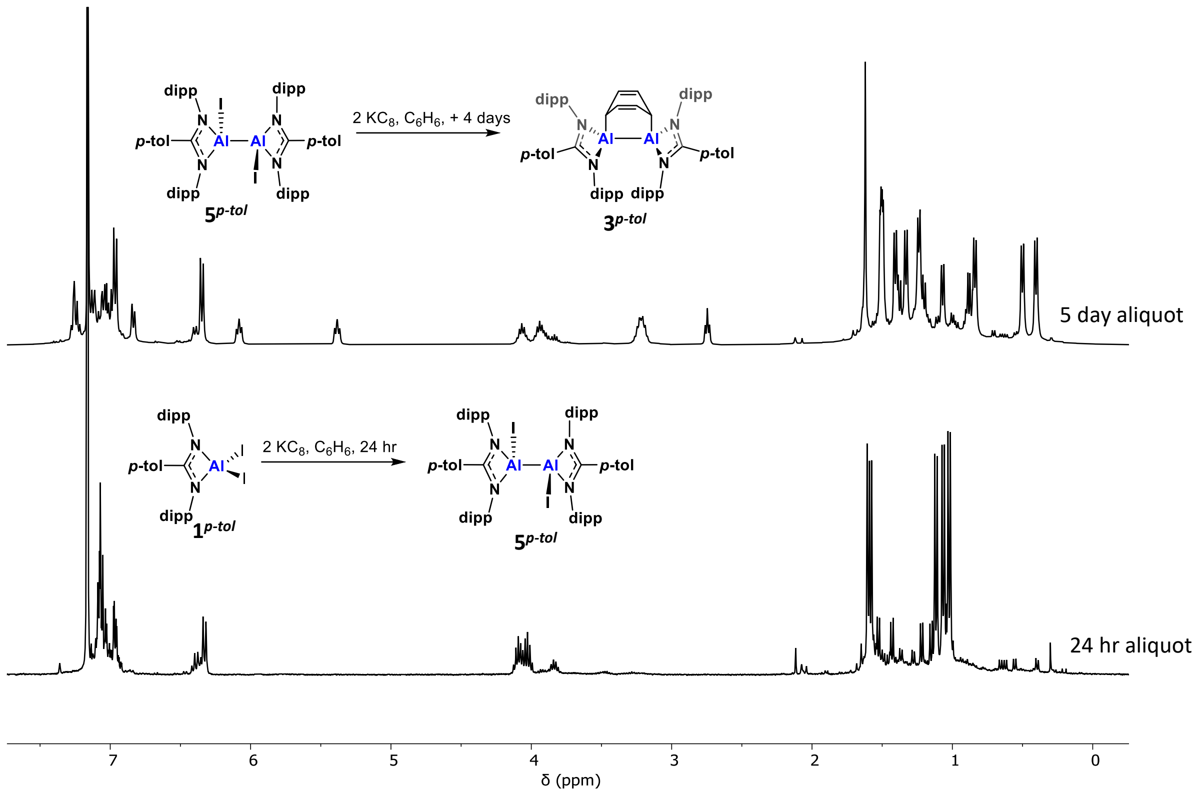

Figure S 6: ^{1}H NMR (400 MHz, 298 K, benzene-d$_6$) spectra showing reduction of ***1$^{p\text{-}tol}$*** *in C_6H_6 initially forming diiododialane* ***5$^{p\text{-}tol}$****, which is further reduced over 4 days to make* ***3$^{p\text{-}tol}$****.*

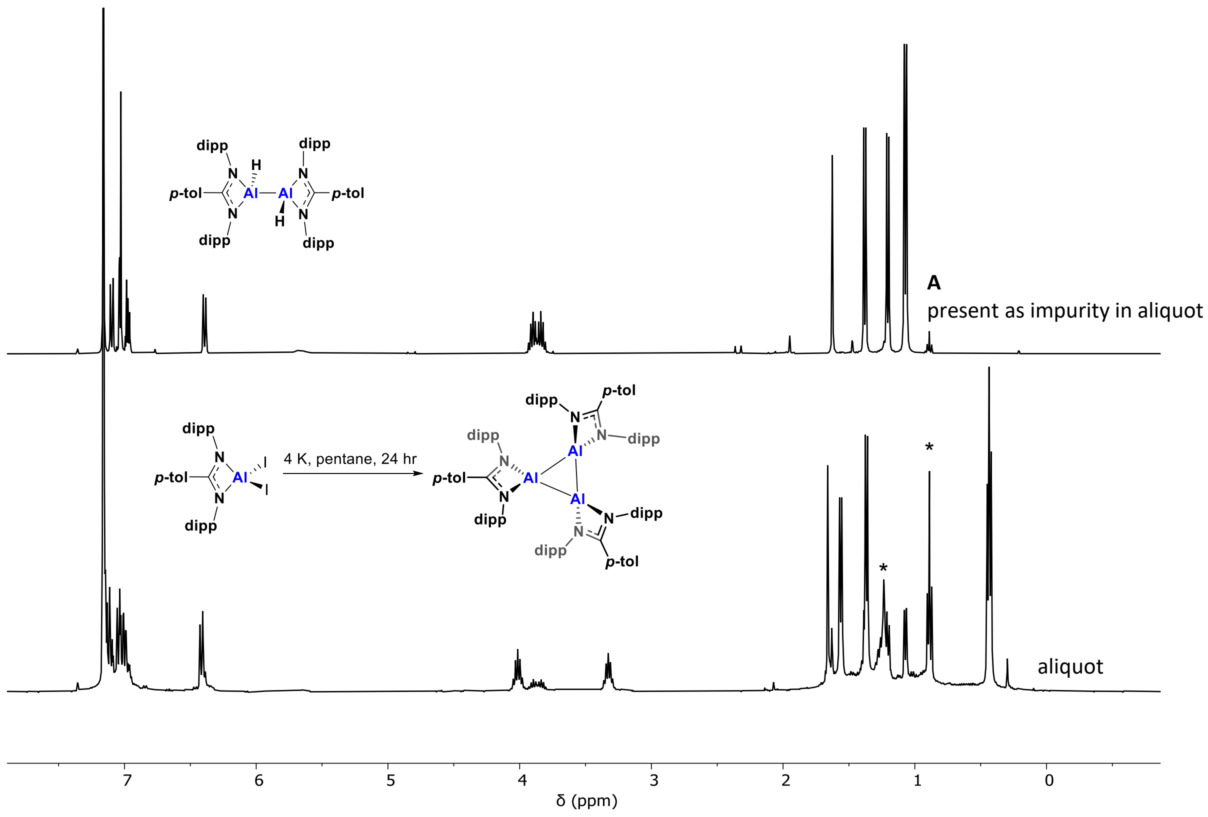

Figure S 7: ^{1}H NMR (400 MHz, 298 K, benzene-d$_6$) spectra showing aliquot from route 1 synthesis of ***2$^{p\text{-}tol}$*** *containing* ***A*** *(*pentane).*

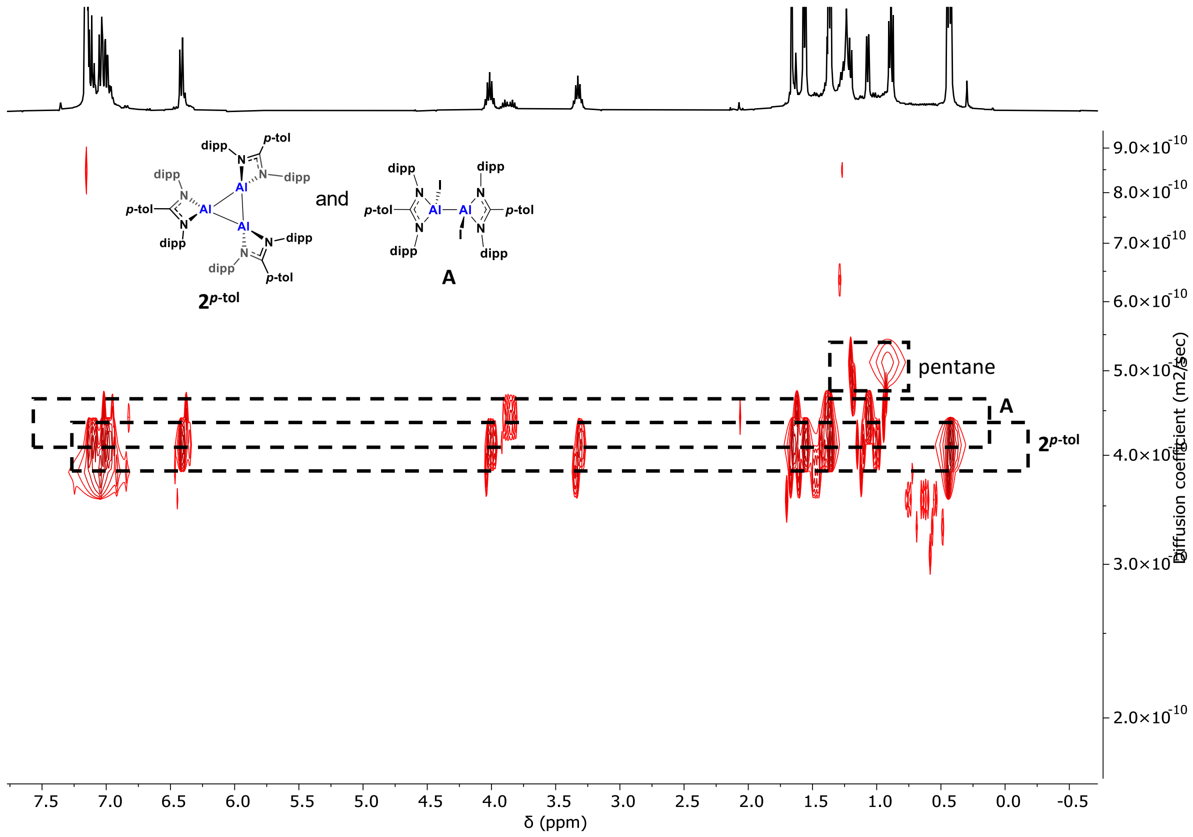

*Figure S 8: DOSY NMR (400 MHz, 298 K, benzene-d$_6$) spectrum showing a larger diffusion coefficient for **2**$^{p\text{-tol}}$ than **A**.*

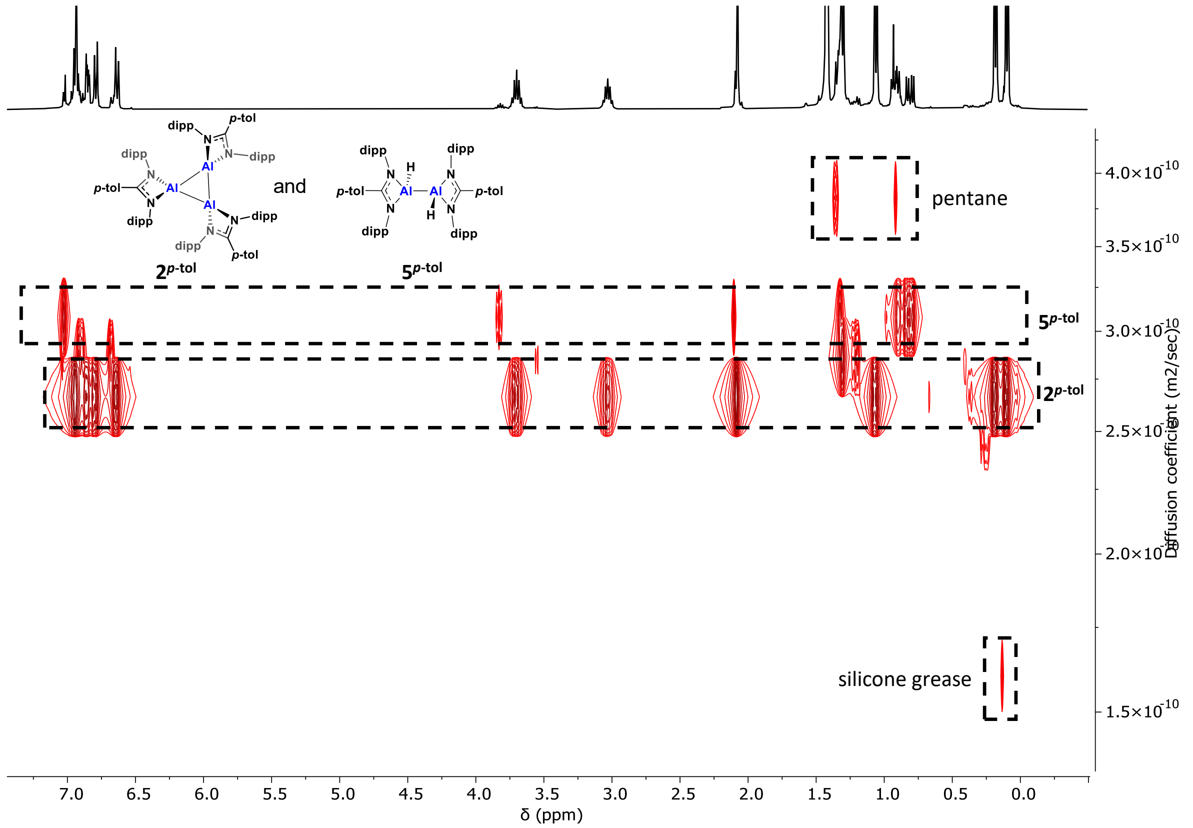

*Figure S 9: DOSY NMR (400 MHz, 298 K, benzene-d$_6$) spectrum showing a larger diffusion coefficient for **2**$^{p\text{-tol}}$ than **5**$^{p\text{-tol}}$.*

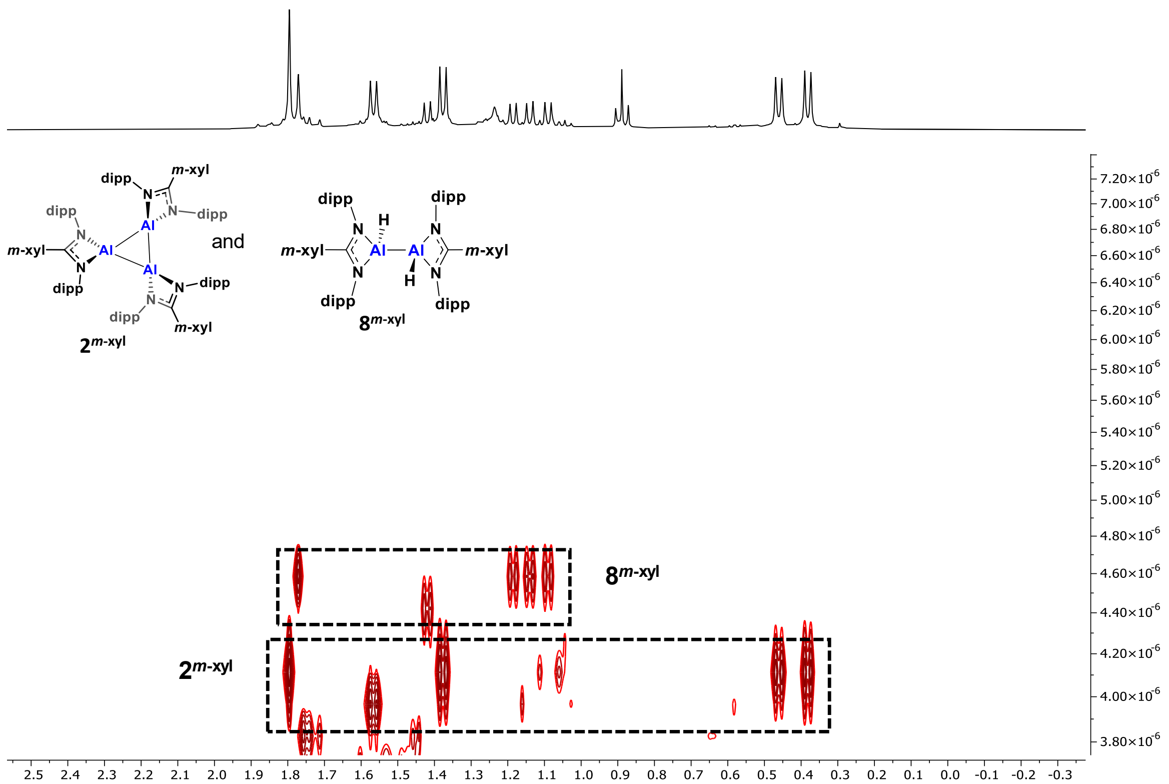

Figure S 10: DOSY NMR (400 MHz, 298 K, benzene-d$_6$) spectrum in benzene-d$_6$ of ***2****$^{m\text{-xyl}}$ and* ***8****$^{m\text{-xyl}}$ showing differing diffusion coefficients.*

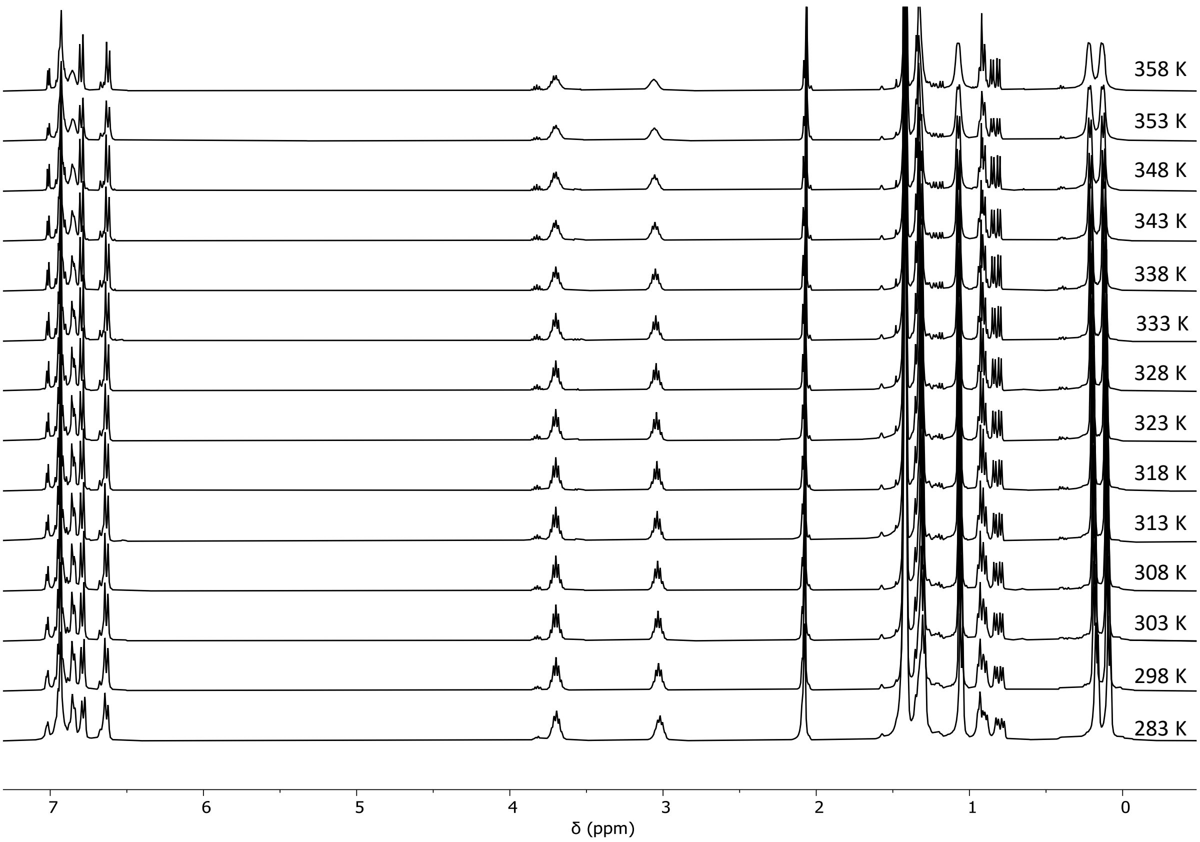

Figure S 11: ^{1}H VT-NMR (400 MHz, 283-358 K, benzene-d$_6$) experiment of ***2****$^{p\text{-tol}}$ in C_6D_6 showing no evidence of dissociation of the trimer up to 358 K.*

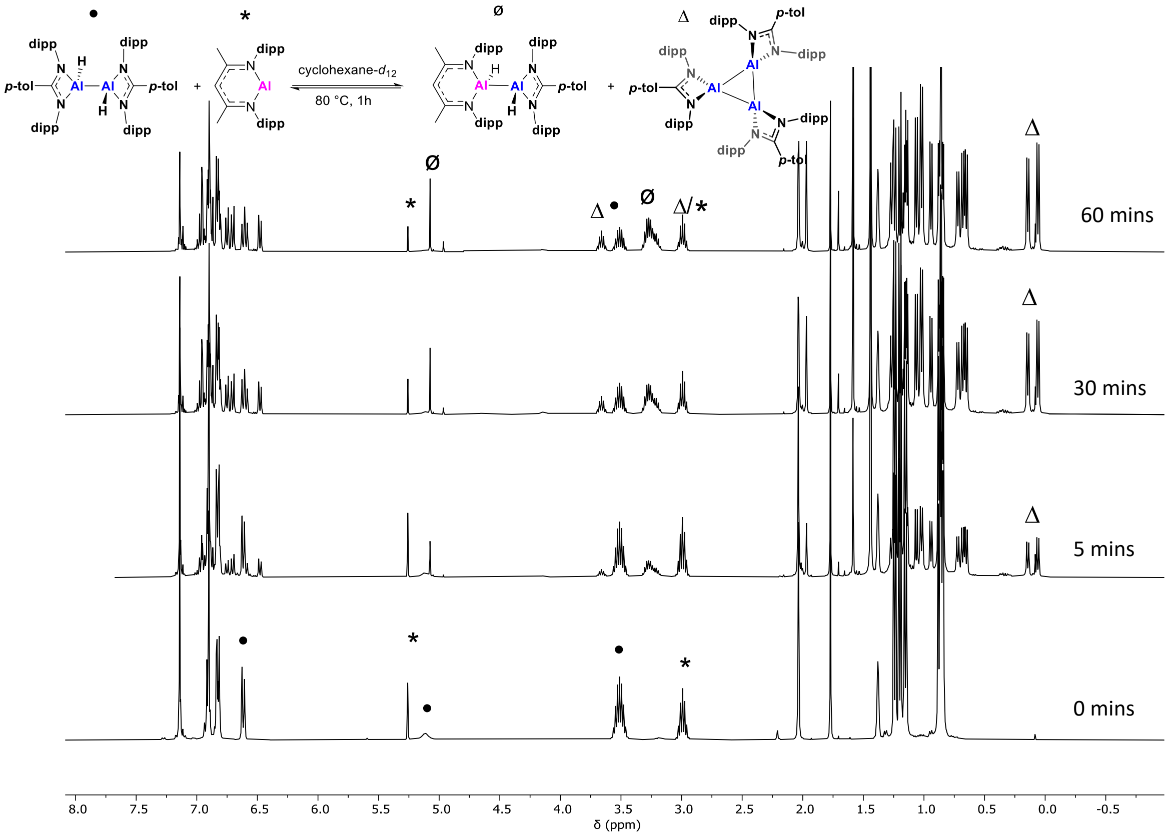

*Figure S 12: ^{1}H NMR (400 MHz, 298 K, benzene-d_6) spectra showing reaction progress for route 2 synthesis of **2**$^{p\text{-}tol}$.*

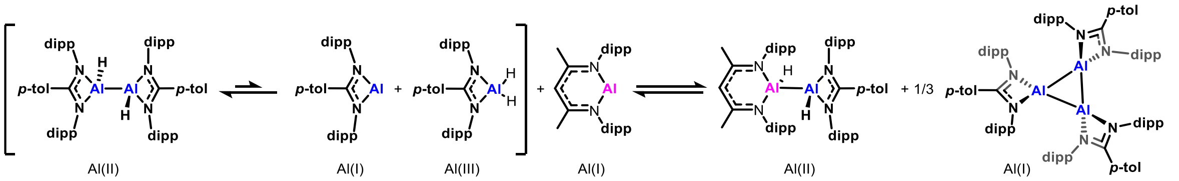

*Figure S 13: Reaction scheme showing putative disproportionation of **A** and subsequent trimerisation forming **2**$^{p\text{-}tol}$.*

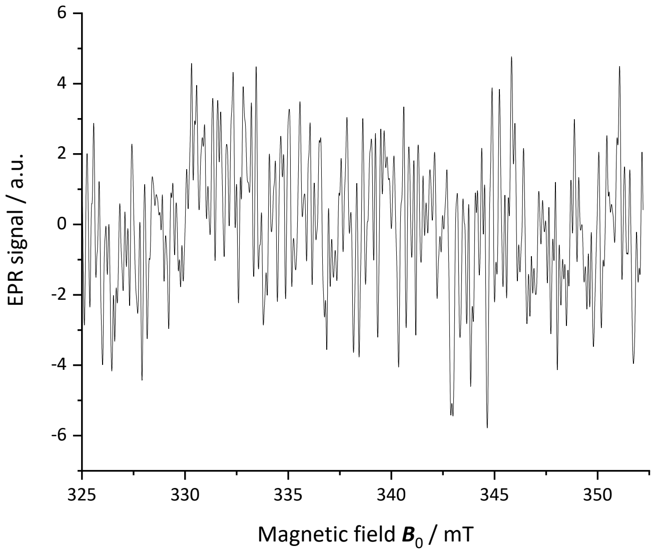

*Figure S 14: EPR spectrum of **2**$^{p\text{-}tol}$ in 3-methylcyclopentane, RT, 1 mW, 0.02 mT, showing no signal.*

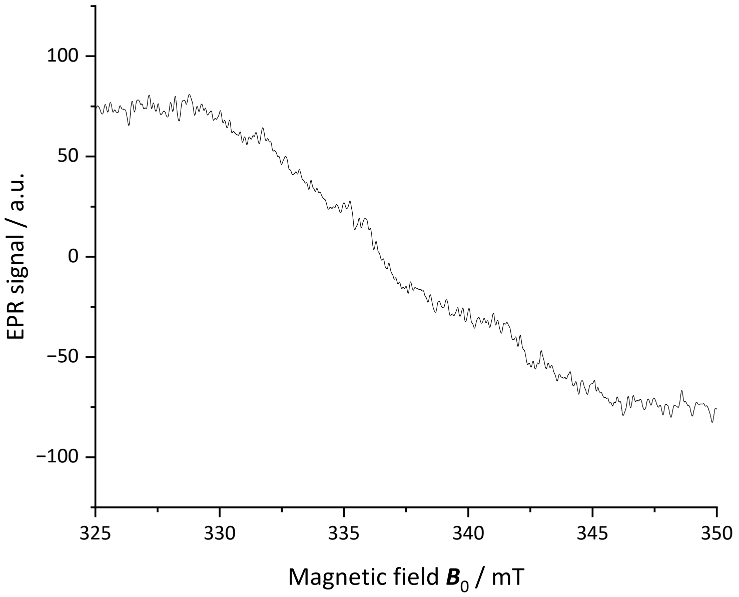

*Figure S 15: EPR spectrum of **2**$^{p\text{-}tol}$ in 3-methylcyclopentane, RT, 10mW, 1mT, showing no signal.*

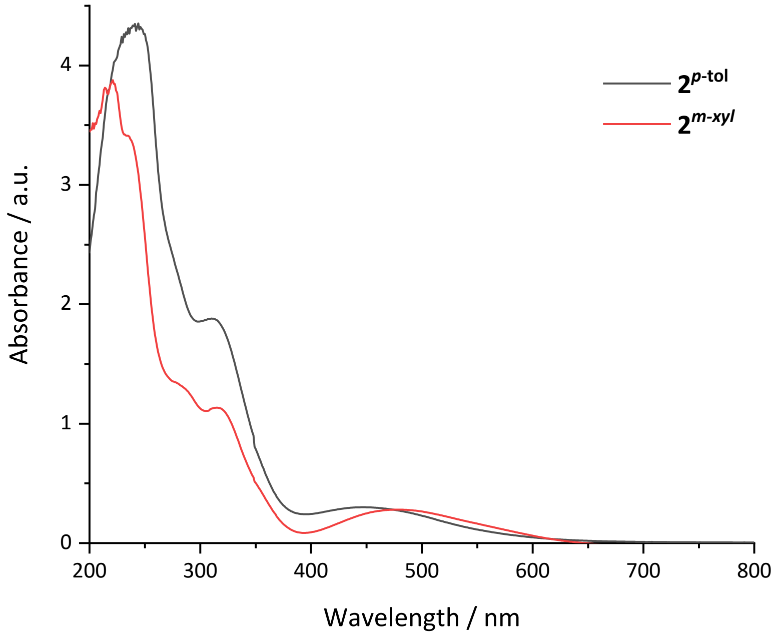

Figure S 16: Experimental UV-vis of ***2****$^{p\text{-}tol}$ and* ***2****$^{m\text{-}xyl}$ (cyclohexane solution).*

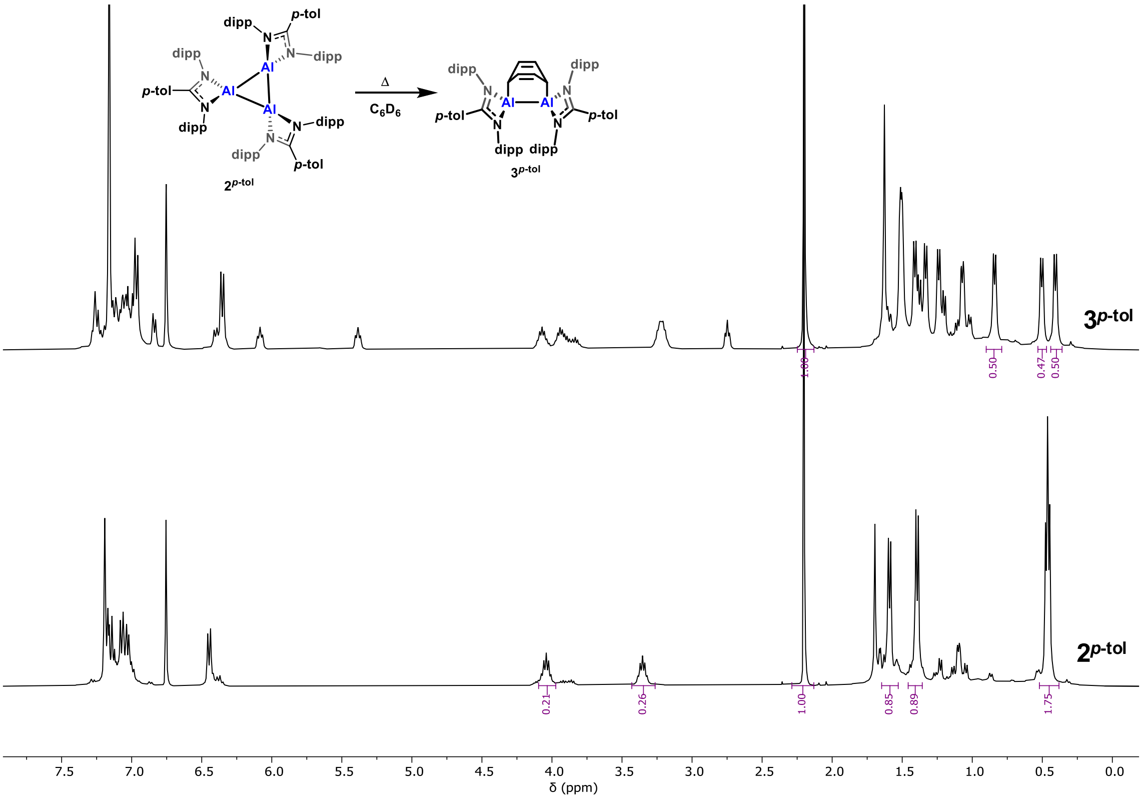

Figure S 17: ^{1}H NMR (400 MHz, 298 K, benzene-d$_6$) spectra showing ***2****$^{p\text{-}tol}$ before and after heating in benzene (non-deuterated) for 2 hours at 80 °C.*

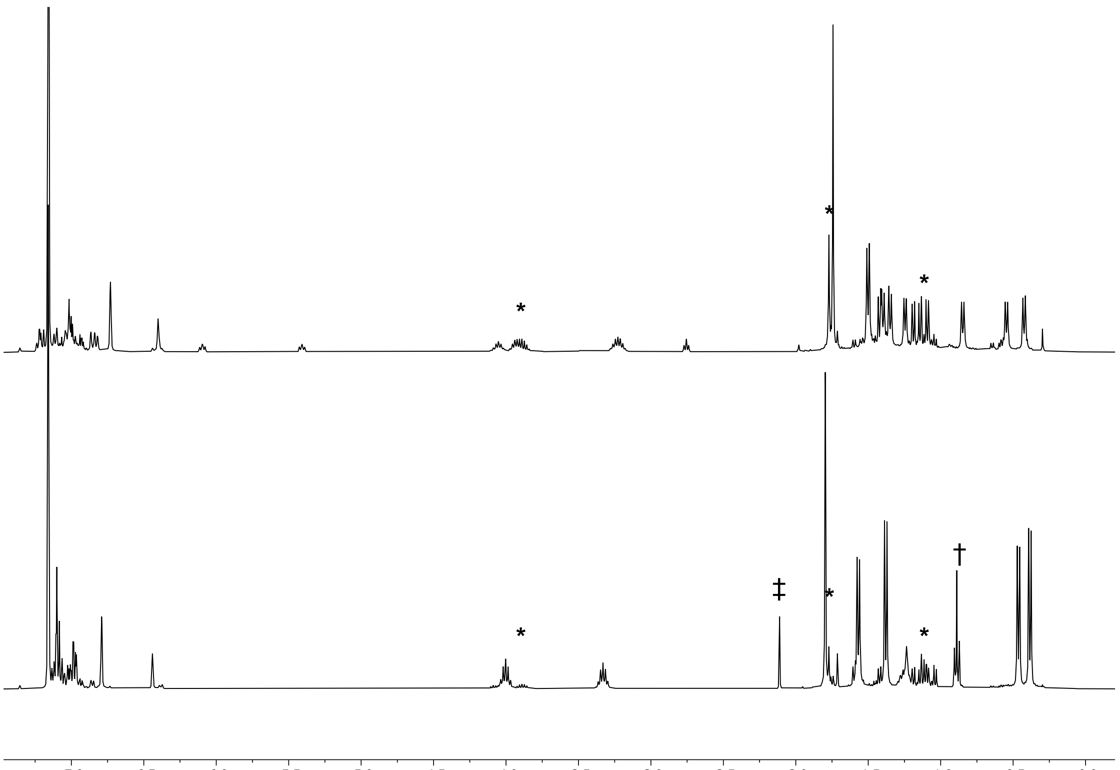

*Figure S 18: ^{1}H NMR (400 MHz, 298 K, benzene-d_6) spectra showing **2**$^{m\text{-}xyl}$ prior to treatment with C_6H_6 (below, some **8**$^{m\text{-}xyl}$ impurity, marked with stars, and pentane (†) and toluene (‡) impurities) and subsequent to heating to 80 °C for 2 hours (above, some **8**$^{m\text{-}xyl}$ impurity, marked with stars).*

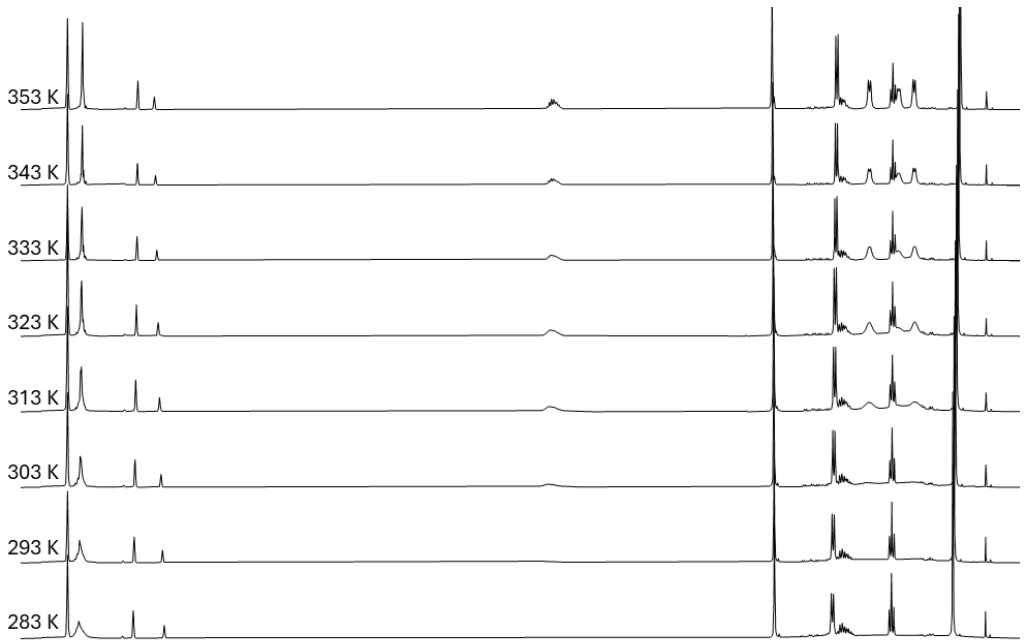

*Figure S 19: ^{1}H VT NMR (400 MHz, 283-353 K, benzene-d_6) experiment of **6**$^{m\text{-}xyl}$ in benzene-d_6 showing resolution of broad resonances corresponding to the iso-propyl protons.*

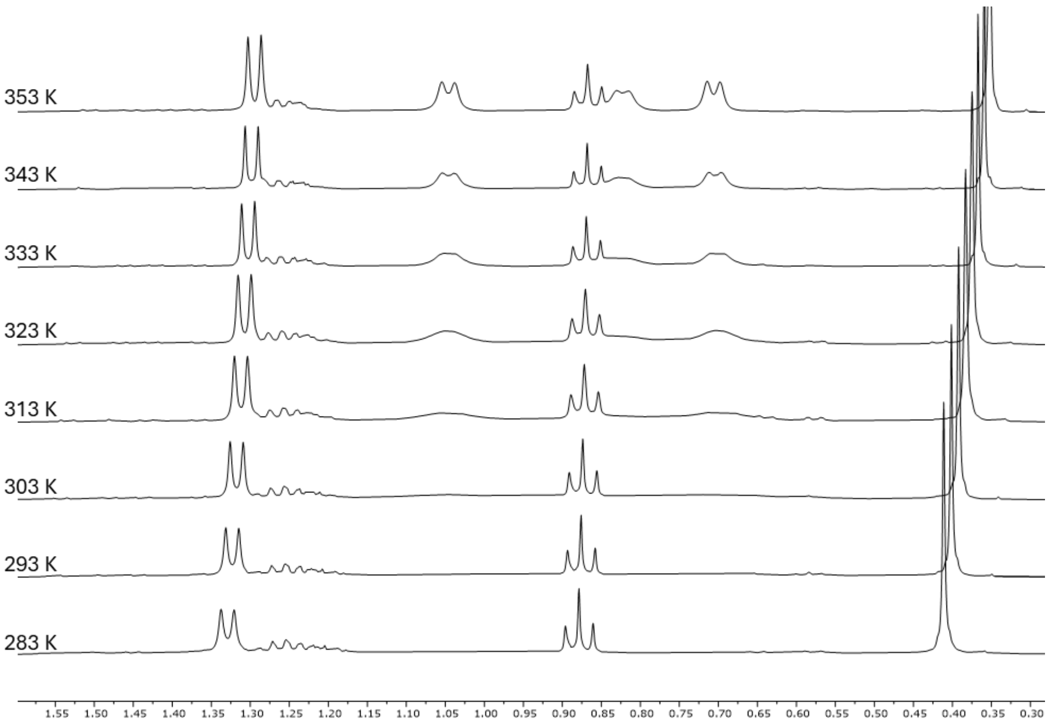

*Figure S 20: ¹H VT NMR (400 MHz, 283-353 K, benzene-d$_6$) experiment of **6**$^{m\text{-}xyl}$ in benzene-d$_6$ the region 0.30-1.60 ppm showing resolution of broad resonances corresponding to the iso-propyl protons.*

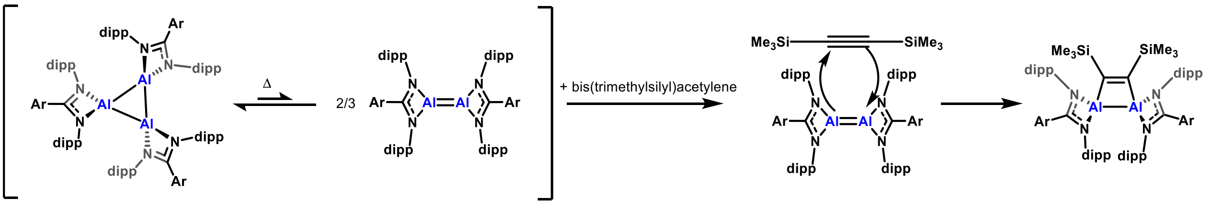

Figure S 21: Proposed [2+2] cycloaddition of a transiently formed dialumene from dissociated trimer and TMS acetylene.

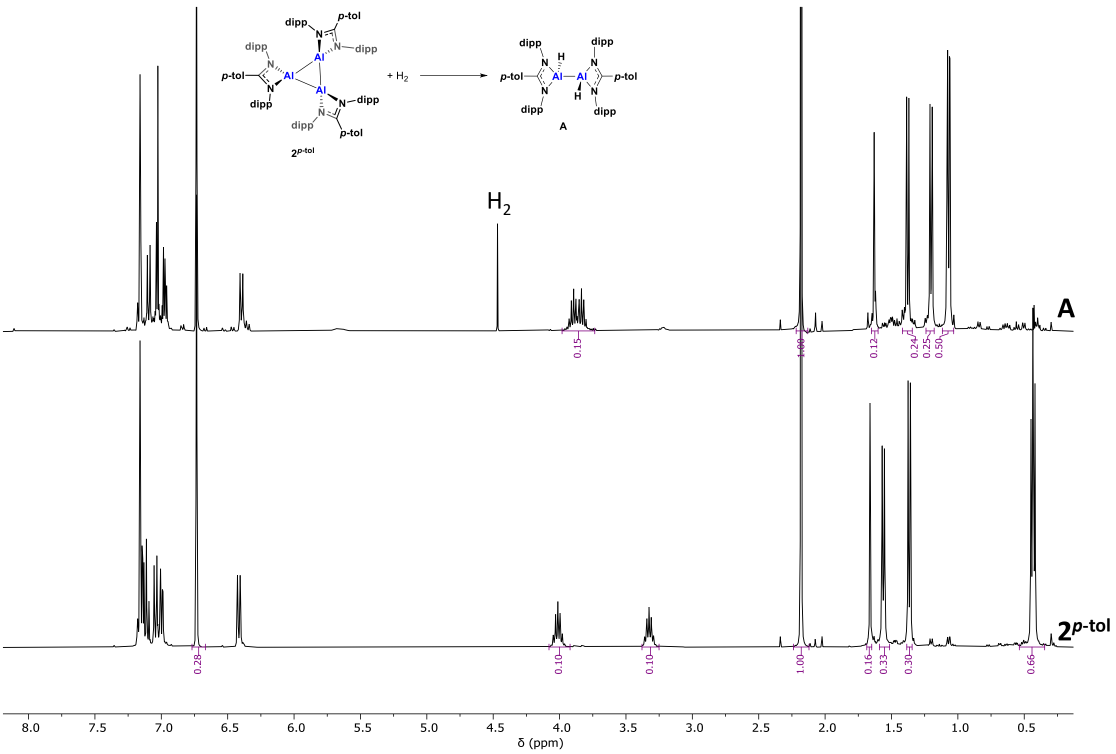

*Figure S 22: ^{1}H NMR (400 MHz, 298 K, benzene-d$_6$) spectra with mesitylene internal standard of reaction from **2$^{p\text{-}tol}$** to form **A**.*

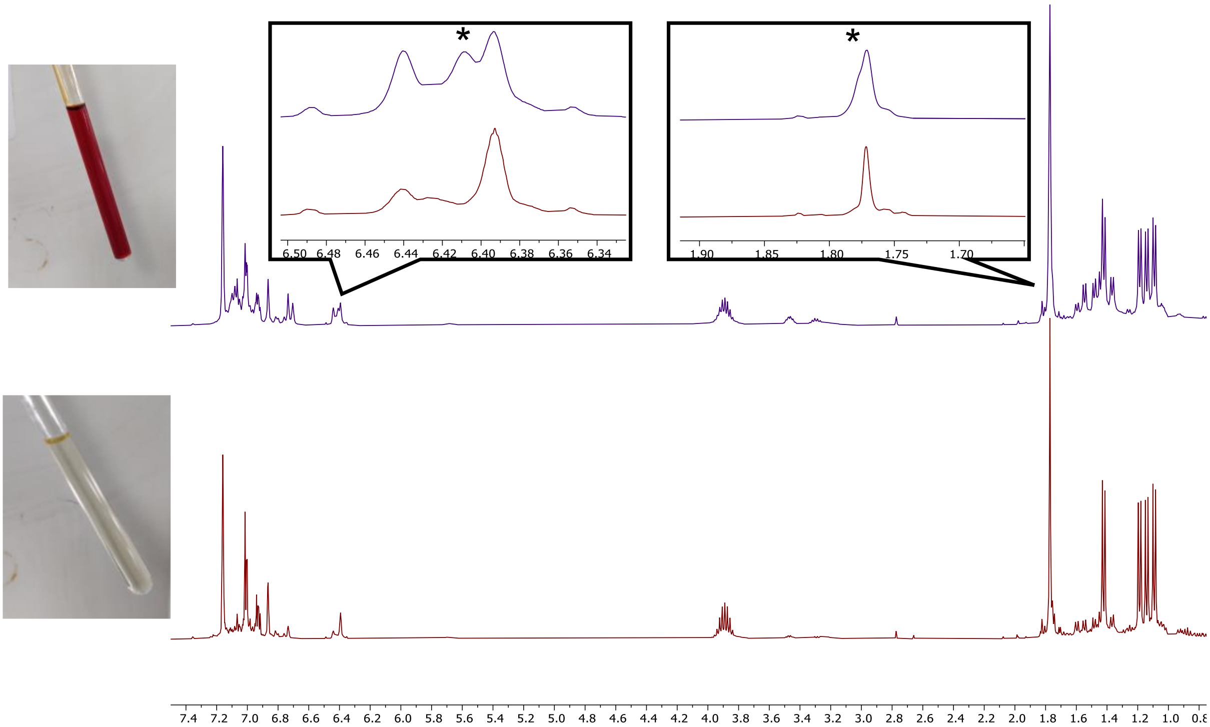

*Figure S 23: Stacked ^{1}H NMR (400 MHz, 298 K, benzene-d$_6$) spectra of **8$^{m\text{-}xyl}$** (bottom) and **8$^{m\text{-}xyl}$** immediately subsequent to H_2 addition to **2$^{m\text{-}xyl}$** showing evidence of intermediate species (inset, top, starred resonances). It is notable that only resonances corresponding to meta-xylyl protons are observed, potentially indicating a cluster-type species. Images of NMR tubes showing notable red colour where intermediate species is present.*

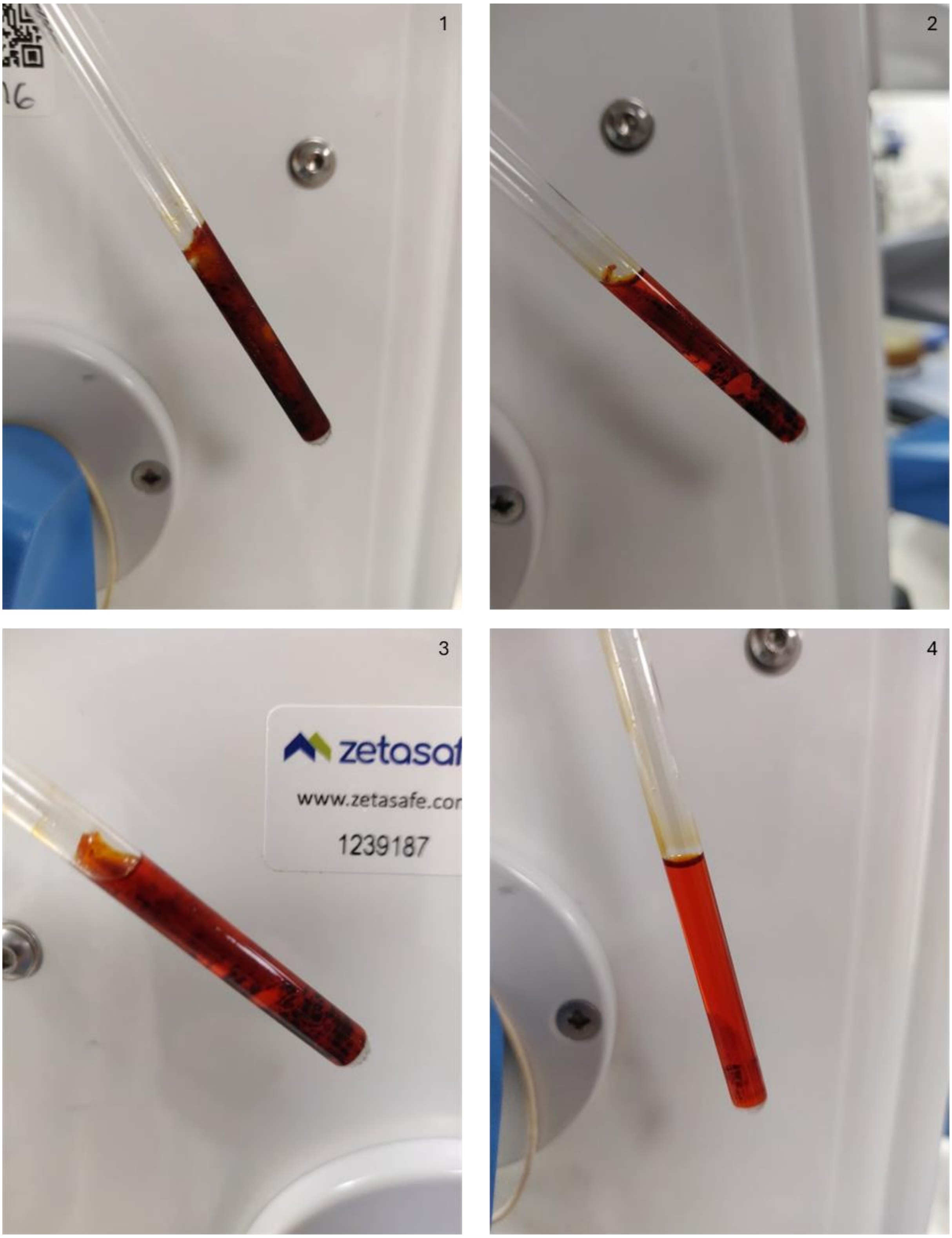

*Figure S 24: Colour progression of the reaction of **2**$^{m\text{-}xyl}$ immediately subsequent to the addition of 1 bar of ethylene gas to afford **9**$^{m\text{-}xyl}$.*

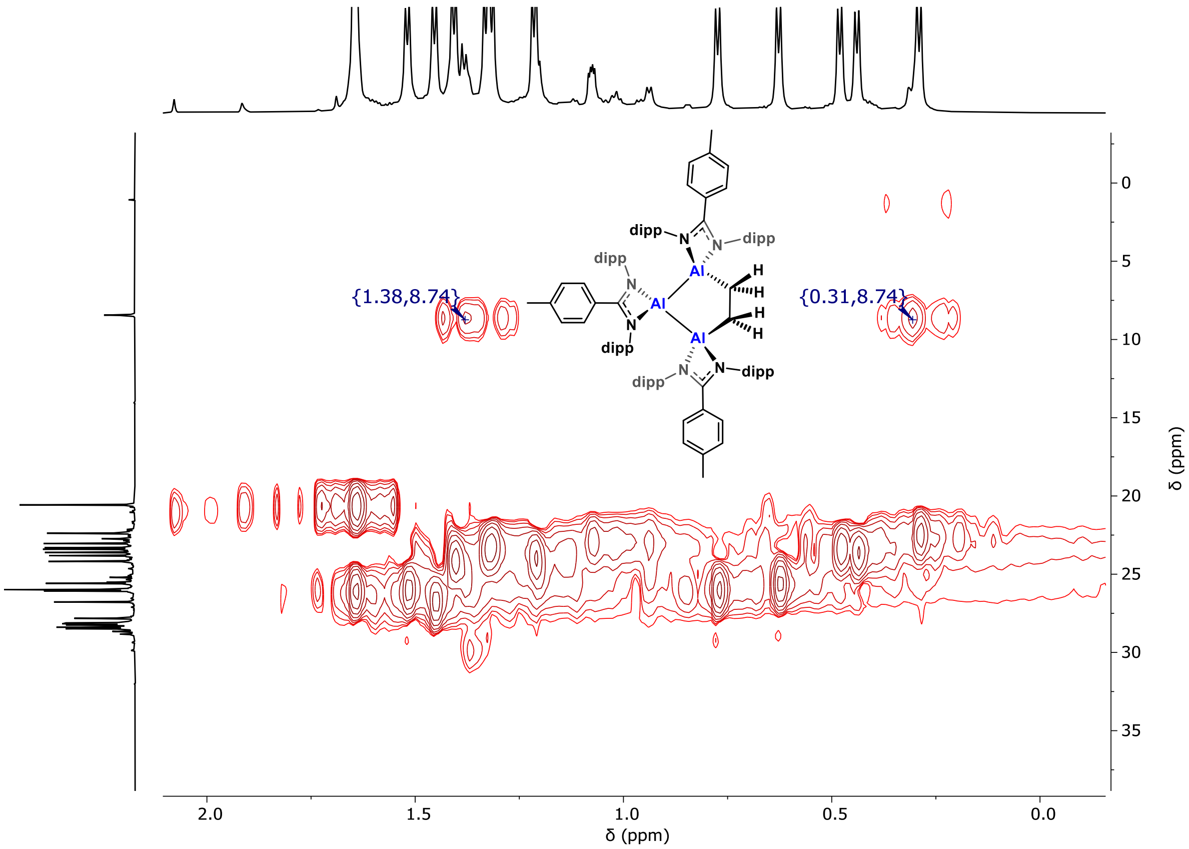

*Figure S 25: HSQC NMR (400, 101 MHz, 298 K, benzene-d$_6$) of compound **9**$^{p\text{-}tol}$ with C-H resonances from ethylene fragment marked.*

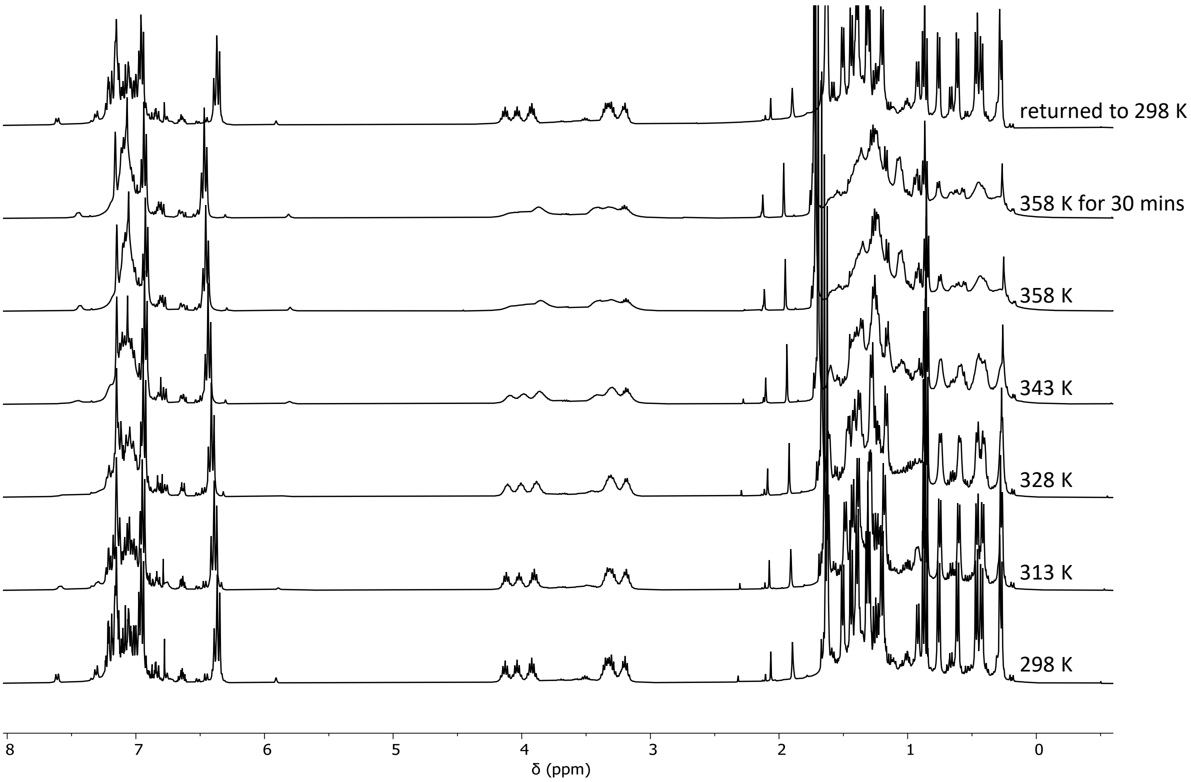

*Figure S 26: ^{1}H VT NMR (400 MHz, 298-358 K, benzene-d$_6$) experiment of **9**$^{p\text{-}tol}$ in benzene-d$_6$ showing no formation of ethene (δ$_H$ = 5.25 ppm) after holding at 358 K for 30 minutes, and then returning sample to room temperature*

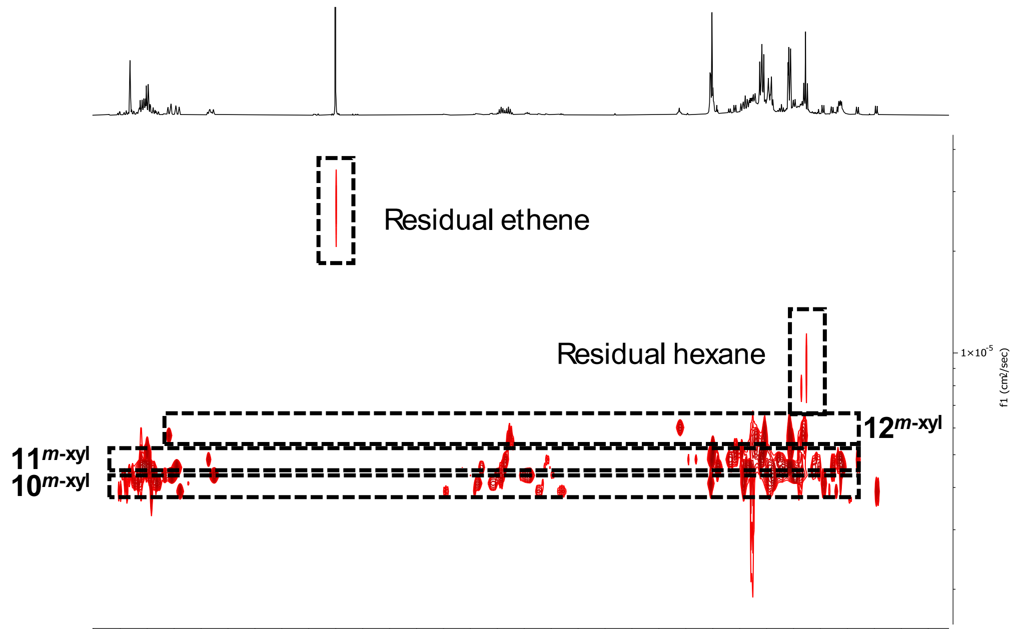

*Figure S 27: DOSY NMR (400 MHz, 298 K, benzene-d$_6$) spectrum of **10**, **11** and **12**$^{m\text{-}xyl}$ showing differing diffusion coefficients for all three species.*

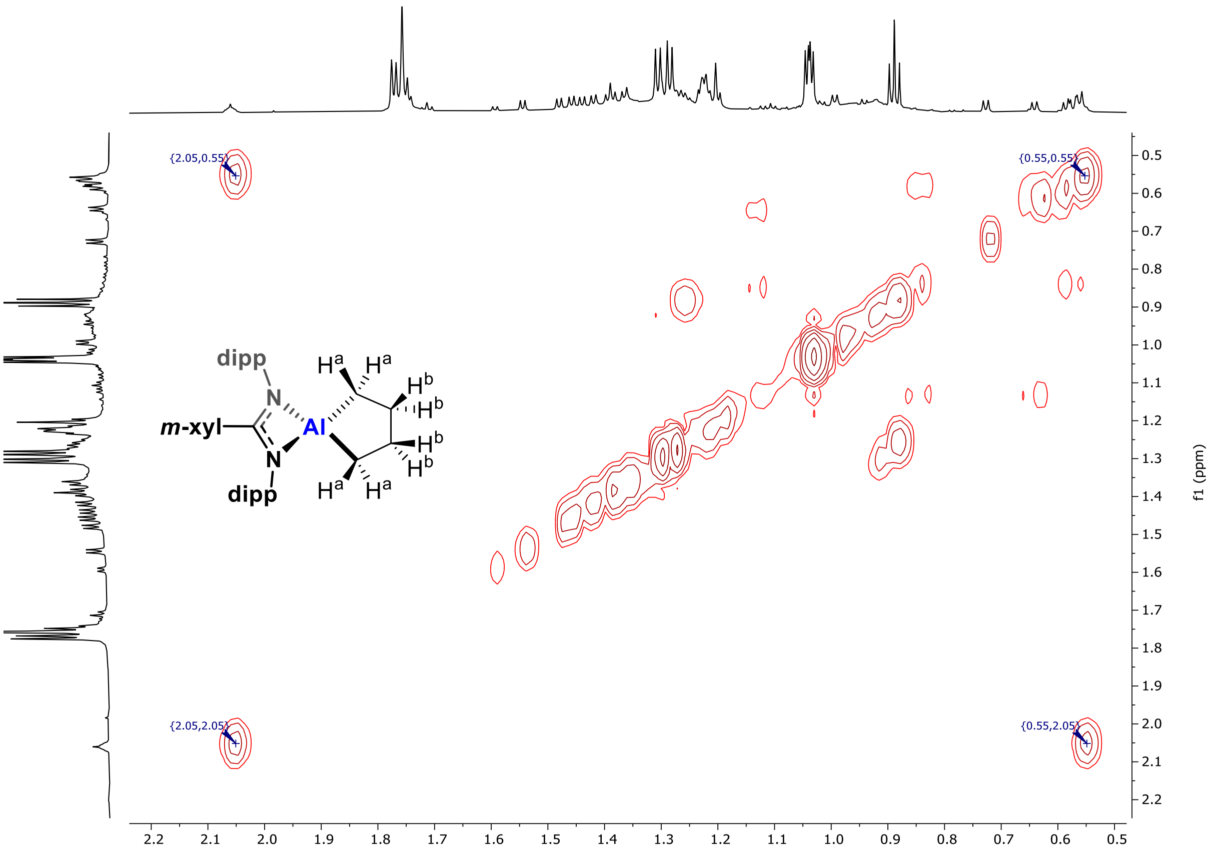

*Figure S 28: ^{1}H-^{1}H COSY NMR (400 MHz, 298 K, benzene-d$_6$) spectrum showing crosspeaks indicating coupling between resonances at 0.55 and 2.05 ppm, corresponding to the Al**C**H$_2$CH$_2$ and AlCH$_2$**C**H$_2$ protons, respectively, from a mixture of **10**, **11** and **12**$^{m\text{-}xyl}$.*

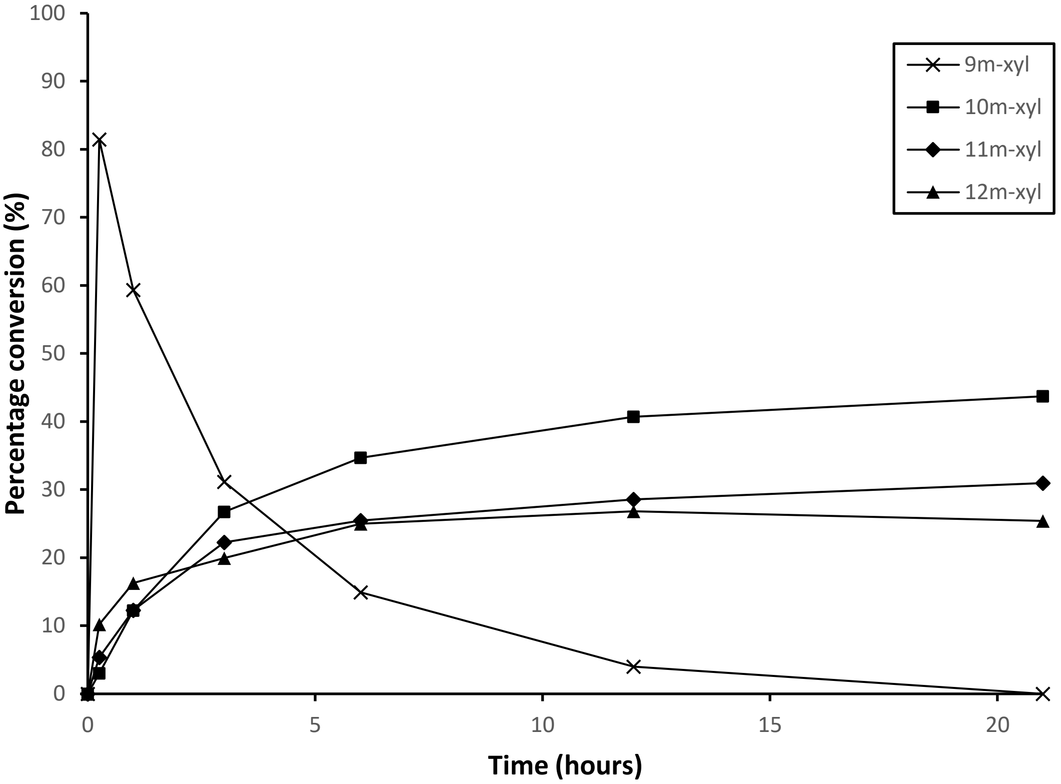

Figure S 29: Chart showing the percentage conversion of ***2**$^{m\text{-}xyl}$* *to* ***9**$^{m\text{-}xyl}$*, ***10**$^{m\text{-}xyl}$*, ***11**$^{m\text{-}xyl}$* *and* ***12**$^{m\text{-}xyl}$* *over a period of 21 hours*

4. X-ray crystallographic data

All experiments were performed at 150 K, 120 K or 100 K using a Cu $K\alpha$ radiation source (λ = 1.54184 Å) or at 100 K using Mo $K\alpha$ (λ = 0.7107 Å) radiation. Measurements on **$2^{m\text{-xyl}}$-1**, **$9^{m\text{-xyl}}$** and **$10^{m\text{-xyl}}$** were obtained using a dual-source Agilent Oxford SuperNova diffractometer with an Atlas CCD detector. Measurements on **$2^{p\text{-tol}}$** were obtained using a dual-source Oxford Diffraction Xcaliubur Gemini diffractometer with a Sapphire 3 CCD plate. Measurements on **$1^{p\text{-tol}}$**, **$2^{p\text{-tol}}$-1**, **$5^{p\text{-tol}}$**, **$5^{m\text{-xyl}}$**, **$2^{m\text{-xyl}}$**, **$8^{m\text{-xyl}}$** and **$11^{m\text{-xyl}}$** were obtained using a dual-souce Synergy-S diffractometer equipped with a Dectris Eiger2 1M detector. Measurements on **$11^{p\text{-tol}}$** were obtained using a dual-source XtaLAB Synergy DW system diffractometer with a HyPix-Arc 100 detector.

Cell refinement, data collection and data reduction for all experiments were performed using Rigaku CrysAlisPro.[11] All structures were solved with ShelXT[12] and ShelXL[13], both programs implemented within the Olex2[14] suite. All atoms, except hydrogen, had atomic coordinates and anisotropic thermal parameters refined to convergence using least-square methods on F^2. Only hydrogen atoms bound to carbons are present, and they were placed in geometric positions and refined with riding modes.

Single crystal X-ray data for $1^{p\text{-tol}}$

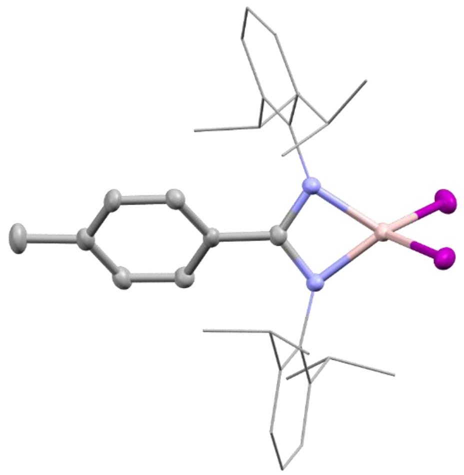

*Figure S 30: The solid-state structure of **$1^{p\text{-tol}}$**. Hydrogen atoms omitted for clarity, key atoms shown as thermal ellipsoids at 50% probability.*

Single crystals of **$1^{p\text{-tol}}$** were grown from slow evaporation of a saturated toluene solution. **$1^{p\text{-tol}}$** was found to crystallise in the $P2_1/c$ space group. The unit cell contained four molecules of toluene per asymmetric unit.

$C_{39}H_{49}AlI_2N_2$ (M =826.58 g/mol): monoclinic, space group $P2_1/c$ (no. 14), a = 13.4874(5) Å, b = 18.0877(5) Å, c = 16.4750(4) Å, β = 104.851(3)°, V = 3884.9(2) Å^3, Z = 4, T = 100.0(5) K, μ(Mo Kα) = 1.669 mm^{-1}, D_{calc} = 1.413 g/cm^3, 28221 reflections measured (4.504° ≤ 2Θ ≤ 61.866°), 9424 unique (R_{int} = 0.0479, R_{sigma} = 0.0521) which were used in all calculations. The final R_1 was 0.0371 (I > 2σ(I)) and wR_2 was 0.0981 (all data). CCDC 2469911.

*Table S 1: Selected bond lengths (Å) for **1**$^{p\text{-}tol}$*

**Al(1)-I(1)**	**Al(1)-I(2)**	**Al(1)-N(1)**	**Al1-N2**
2.4934(8)	2.4903(8)	1.890(2)	1.898(2)

*Table S 2: Selected angles (°) for **1**$^{p\text{-}tol}$*

**N(1)-Al(1)-N(2)**	**N(1)-C(1)-N(2)**
70.78(9)	35.52(13)

***Single crystal X-ray data for 2**$^{p\text{-}tol}$ – first polymorph*

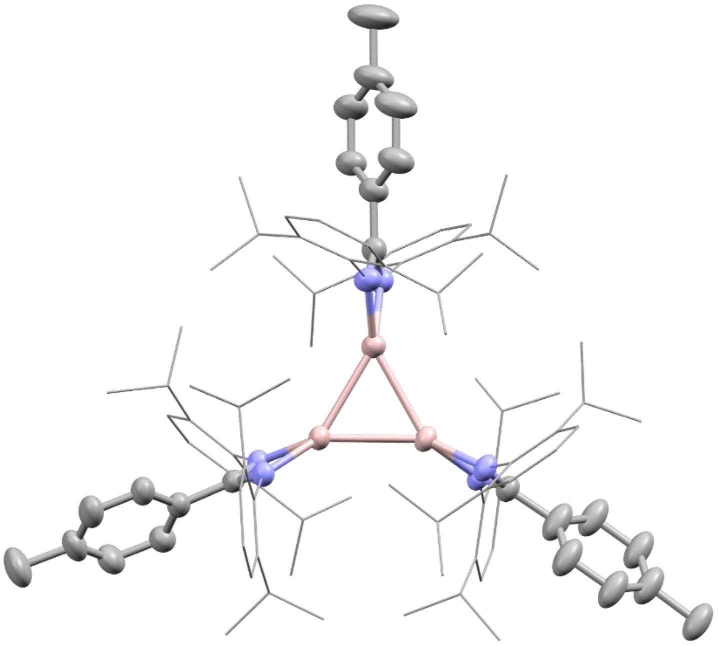

*Figure S 31: The solid-state structure of **2**$^{p\text{-}tol}$**-A**. Hydrogen atoms, second molecule **2**$^{p\text{-}tol}$**-B** and disorder omitted for clarity, key atoms shown as thermal ellipsoids at 50% probability.*

Single crystals of **2**$^{p\text{-}tol}$ were grown by cooling a saturated hexane solution. **2**$^{p\text{-}tol}$ was found to crystallise in the $P\bar{1}$ space group, with two molecules in the unit cell (**2**$^{p\text{-}tol}$**-A** and **2**$^{p\text{-}tol}$**-B**). A solvent mask (SQUEEZE) was applied to remove the electron density of 4 hexanes per asymmetric unit. There was disorder across 4 diisopropyl groups, each of which were modelled across two positions.

There is residual electron density located off the Al-Al bonds in both molecules, corresponding to an average weight of 0.48 for **2**$^{p\text{-}tol}$**-A** and 0.42 for **2**$^{p\text{-}tol}$**-B**. This density is also seen when the solvent mask (SQUEEZE) is removed. While modelling this density as a hydride can lead to a stable refinement, the density is better explained by the electron rich Al–Al bonding interactions (see Section 5).

$C_{216}H_{302}Al_6N_{12}$ (M =3228.56 g/mol): triclinic, space group $P\bar{1}$ (no. 2), a = 14.7364(4) Å, b = 24.9378(6) Å, c = 30.9657(8) Å, α = 70.319(2)°, β = 76.958(2)°, γ = 76.507(2)°, V = 10283.8(5) Å^3, Z = 2, T = 150.00(10) K, μ(Cu Kα) = 0.681 mm^{-1}, $Dcalc$ = 1.043 g/cm^3, 154881 reflections measured (6.506° ≤ 2Θ ≤ 144.986°), 39700 unique (R_{int} = 0.0603, R_{sigma} = 0.0602) which were used in all calculations. The final R_1 was 0.0706 (I > 2σ(I)) and wR_2 was 0.2160 (all data). CCDC 2469912.

Table S 3: Selected bond lengths (Å) for ***2$^{p\text{-}tol}$-A***

**Al(1)-Al(2)**	**Al(2)-Al(3)**	**Al(3)-Al(1)**			
2.6575(10)	2.6530(12)	2.6308(11)			
**Al(1)-N(1)**	**Al(1)-N(2)**	**Al(2)-N(3)**	**Al(2)-N(4)**	**Al(3)-N(5)**	**Al(3)-N(6)**
1.983(3)	1.960(2)	1.969(2)	1.974(2)	1.977(2)	1.970(2)

Table S 4: Selected bond lengths (Å) for ***2$^{p\text{-}tol}$-B***

**Al(4)-Al(5)**	**Al(5)-Al(6)**	**Al(6)-Al(4)**			
2.6641(12)	2.6553(11)	2.6604(10)			
**Al(4)-N(7)**	**Al(4)-N(8)**	**Al(5)-N(9)**	**Al(5)-N(10)**	**Al(6)-N(11)**	**Al(6)-N(12)**
1.992(3)	1.975(2)	1.983(2)	1.980(1)	1.980(2)	1.975(2)

Table S 5: Selected angles (°) for ***2$^{p\text{-}tol}$-A***

**Al(1)-Al(2)-Al(3)**	**Al(2)-Al(3)-Al(1)**	**Al(3)-Al(1)-Al(2)**
59.39(3)	60.39(3)	60.22(3)
**N(1)-Al(1)-N(2)**	**N(3)-Al(2)-N(4)**	**N(5)-Al(3)-N(6)**
67.51(10)	67.51(10)	67.35(10)

Table S 6: Selected angles (°) for ***2$^{p\text{-}tol}$-B***

**Al(4)-Al(6)-Al(5)**	**Al(4)-Al(5)-Al(6)**	**Al(5)-Al(4)-Al(6)**
60.16(3)	60.02(3)	59.83(3)
**N(7)-Al(4)-N(8)**	**N(9)-Al(5)-N(10)**	**N(11)-Al(6)-N(12)**
67.27(10)	67.12(9)	67.35(10)

Single crystal X-ray data for 2$^{p\text{-}tol}$-1 *– second polymorph*

A second polymorph of **2$^{p\text{-}tol}$** was observed when growing crystals by fractionally crystallising away from the products of route 2 in hexane (see Figure S 12). **2$^{p\text{-}tol}$-1** crystallised in the $P\bar{1}$ space group, with two molecules in the asymmetric unit. The structure contained disordered hexane, and an attempt was made to apply a solvent mask (SQUEEZE) to remove this, with two voids containing 1.8 and 1.6 molecules of hexane per asymmetric unit. However, due to an issue with the data collection there were 11 missing reflections below Theta(min), which may affect how reliable the mask is. Therefore, the data is included for completeness but is not discussed in detail in the main manuscript.

$C_{212.4}H_{293.6}Al_6N_{12}$ (M =3176.86 g/mol): triclinic, space group $P\bar{1}$ (no. 2), a = 14.6503(2) Å, b = 27.1548(4) Å, c = 28.0602(4) Å, α = 111.6180(10)°, β = 103.1750(10)°, γ = 92.5320(10)°, V = 10002.9(3) Å^3, Z = 2, T = 150.0(4) K, μ(Cu Kα) = 0.693 mm^{-1}, D_{calc} = 1.055 g/cm^3, 126761 reflections measured (7.074° ≤ 2Θ ≤ 157.278°), 40462 unique (R_{int} = 0.0467, R_{sigma} = 0.0516) which were used in all calculations. The final R_1 was 0.0788 (I > 2σ(I)) and wR_2 was 0.1833 (all data). CCDC 2469920.

Single crystal X-ray data for 2$^{m\text{-}xyl}$ *– first polymorph*

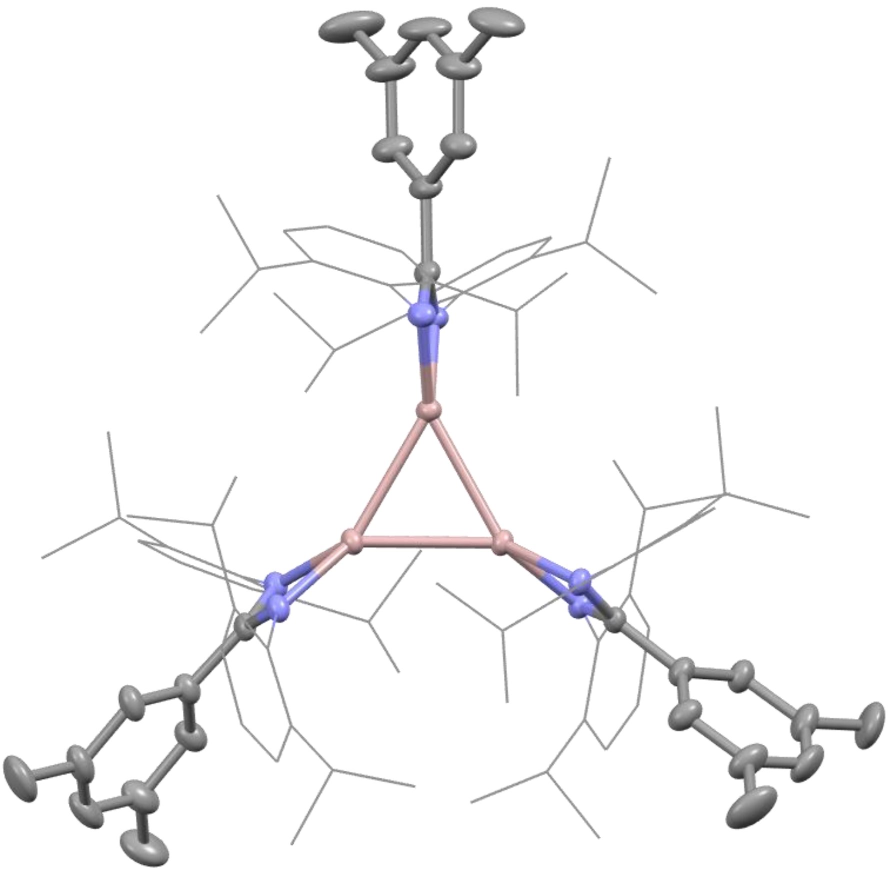

*Figure S 32: The solid-state structure of **2$^{m\text{-}xyl}$** (Solvent molecules and hydrogen atoms omitted, dipp groups wireframe for clarity, thermal ellipsoids 50% probability).*

Single crystals of **2$^{m\text{-}xyl}$** were grown from cooling of a saturated toluene solution. **2$^{m\text{-}xyl}$** was found to crystallise in the $P2_1$/n space group. The unit cell contained two molecules of toluene per asymmetric unit.

$C_{113}H_{145}Al_3N_6$ (M =1668.28 g/mol): monoclinic, space group $P2_1$/n (no. 14), a = 15.42790(10) Å, b = 23.6186(2) Å, c = 28.0831(3) Å, β = 96.7170(10)°, V = 10162.83(15) Å^3, Z = 4, T = 100.0(4) K, μ(Mo Kα) = 0.086 mm^{-1}, D_{calc} = 1.090 g/cm^3, 187504 reflections measured (5.376° ≤ 2Θ ≤ 54.204°), 22378 unique (R_{int} = 0.0869, R_{sigma} = 0.0387) which were used in all calculations. The final R_1 was 0.0668 (I > 2σ(I)) and wR_2 was 0.1895 (all data). CCDC 2469913.

*Table S 7: Selected bond lengths (Å) for **$2^{m\text{-}xyl}$***

Al(1)-Al(2)	Al(2)-Al(3)	Al(1)-Al(3)			
2.6404(8)	2.6184(8)	2.6502(8)			
**Al(1)-N(1)**	**Al(1)-N(2)**	**Al(2)-N(3)**	**Al(2)-N(4)**	**Al(3)-N(5)**	**Al(3)-N(6)**
1.9744(16)	1.9729(16)	1.9767(16)	1.9605(16)	1.9670(17)	1.9767(16)

*Table S 8: Selected angles (°) for **$2^{m\text{-}xyl}$***

Al(1)-Al(2)-Al(3)	Al(2)-Al(3)-Al(1)	Al(3)-Al(1)-Al(2)
60.52(2)	60.15(2)	59.33(2)
**N(1)-Al(1)-N(2)**	**N(3)-Al(2)-N(4)**	**N(5)-Al(3)-N(6)**
67.56(7)	67.70(7)	67.46(6)

***Single crystal X-ray data for $2^{m\text{-}xyl}$-1** – second polymorph*

Single crystals of **$2^{m\text{-}xyl}$-1** were grown from the addition of pentane to a saturated toluene solution of **$2^{m\text{-}xyl}$** and subsequent storage at room temperature overnight. **$2^{m\text{-}xyl}$-1** crystallised in the $P2_1/n$ space group with 2 molecules of pentane in the asymmetric unit, which were modelled using a solvent mask (SQUEEZE). There was disorder over three isopropyl groups, which were modelled over two positions, and one *meta*-xylyl group, which was modelled over two positions.
$C_{109}H_{153}Al_3N_6$ (*M* =1628.30 g/mol): monoclinic, space group $P2_1/n$ (no. 14), a = 15.5037(3) Å, b = 23.5791(4) Å, c = 27.8912(8) Å, β = 96.740(2)°, V = 10125.5(4) Å^3, Z = 4, T = 120.00(10) K, μ(Cu Kα) = 0.695 mm^{-1}, *Dcalc* = 1.068 g/cm^3, 92142 reflections measured (6.856° ≤ 2Θ ≤ 158.454°), 20800 unique (R_{int} = 0.1174, R_{sigma} = 0.1067) which were used in all calculations. The final R_1 was 0.0918 (I > 2σ(I)) and wR_2 was 0.2686 (all data). CCDC 2469921.

*Table S 9: Selected bond lengths (Å) for **$2^{m\text{-}xyl}$-1***

Al(1)-Al(2)	Al(2)-Al(3)	Al(1)-Al(3)			
2.5963(15)	2.6413(17)	2.6046(17)			
**Al(1)-N(1)**	**Al(1)-N(2)**	**Al(2)-N(3)**	**Al(2)-N(4)**	**Al(3)-N(5)**	**Al(3)-N(6)**
1.976(3)	1.955(3)	1.964(3)	1.980(3)	1.964(3)	1.973(3)

*Table S 10: Selected angles (°) for **$2^{m\text{-}xyl}$-1***

Al(1)-Al(2)-Al(3)	Al(2)-Al(3)-Al(1)	Al(3)-Al(1)-Al(2)
59.63(4)	59.32(4)	61.04(4)
**N(1)-Al(1)-N(2)**	**N(3)-Al(2)-N(4)**	**N(5)-Al(3)-N(6)**
67.94(13)	67.40(12)	67.32(14)

Single crystal X-ray data for 5$^{p\text{-}tol}$

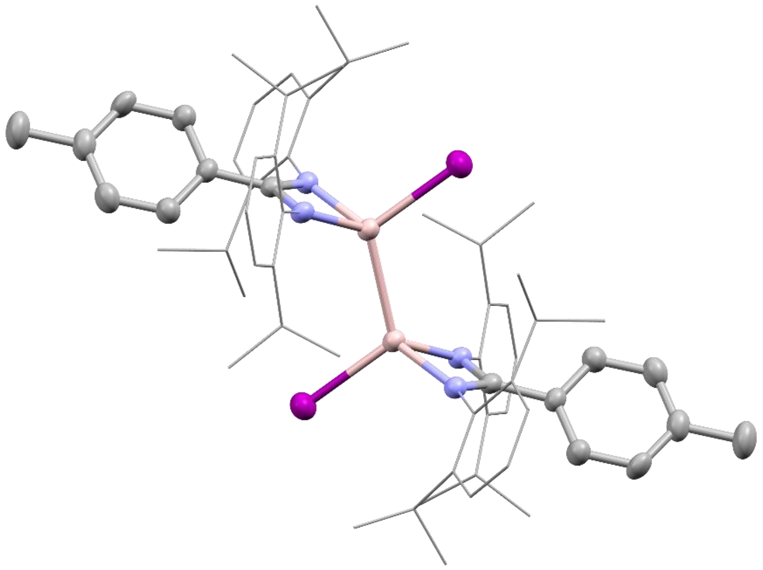

*Figure S 33: The solid-state structure of **5**$^{p\text{-}tol}$. Hydrogen atoms and solvent omitted for clarity, key atoms shown as thermal ellipsoids at 50% probability.*

Single crystals of **5**$^{p\text{-}tol}$ were grown from cooling of a saturated toluene solution. **5**$^{p\text{-}tol}$ was found to crystallise in the $P2_1$/n space group. The unit cell contained 4 molecules of toluene per asymmetric unit.

$C_{78}H_{98}N_4Al_2I_2$ (M =1399.36 g/mol): monoclinic, space group $P2_1$/n (no. 14), a = 10.49250(10) Å, b = 18.2327(2) Å, c = 19.1795(2) Å, β = 98.4090(10)°, V = 3629.72(7) Å^3, Z = 2, T = 151(2) K, μ(Cu Kα) = 7.362 mm^{-1}, D_{calc} = 1.280 g/cm^3, 71485 reflections measured (6.724° ≤ 2Θ ≤ 157.962°), 7519 unique (R_{int} = 0.0948, R_{sigma} = 0.0484) which were used in all calculations. The final R_1 was 0.0581 (I > 2σ(I)) and wR_2 was 0.1674 (all data). CCDC 2469914.

*Table S 11: Selected bond lengths (Å) and angles (°) for **5**$^{p\text{-}tol}$*

**Al(1)-Al(1')**	**Al(1)-N(1)**	**Al(1)-N(2)**	**Al(1)-I(1)**
2.604(2)	1.915(3)	1.952(3)	2.5483(10)

*Table S 12: Selected bond angles (°) for **5**$^{p\text{-}tol}$*

**N(1)-Al(1)-N(2)**	**Al(1')-Al(1)-I(1)**
69.15(13)	114.95(6)

Single crystal X-ray data for 5$^{m\text{-}xyl}$

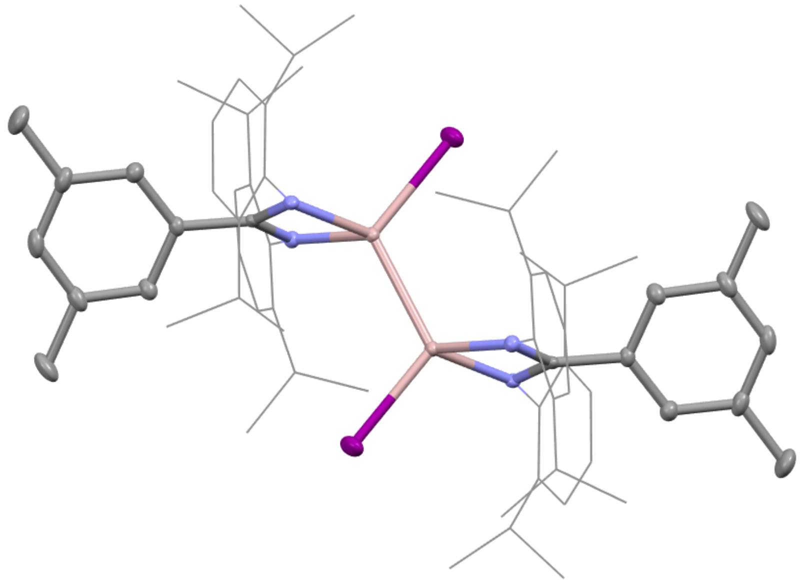

Figure S 34: The solid-state of 5m-xyl (Solvent molecules and hydrogen atoms omitted, dipp groups wireframe for clarity, thermal ellipsoids 50% probability).

Single crystals of **5**$^{m\text{-}xyl}$ were grown from a benzene solution. **5**$^{m\text{-}xyl}$ was found to crystallise in the $P\bar{1}$ space group, with half a molecule in the asymmetric unit. The unit cell contained 1 benzene molecule per asymmetric unit.

$C_{72}H_{92}N_4Al_2I_2$ (M =1321.25 g/mol): triclinic, space group $P\bar{1}$ (no. 2), a = 10.69630(10) Å, b = 12.3546(2) Å, c = 14.0145(2) Å, α = 67.7240(10)°, β = 77.7910(10)°, γ = 87.4630(10)°, V = 1673.70(4) Å^3, Z = 1, T = 100.0(5) K, μ(Mo Kα) = 1.009 mm^{-1}, D_{calc} = 1.311 g/cm^3, 27224 reflections measured (4.5° ≤ 2Θ ≤ 61.906°), 8463 unique (R_{int} = 0.0511, R_{sigma} = 0.0409) which were used in all calculations. The final R_1 was 0.0335 (I > 2σ(I)) and wR_2 was 0.0928 (all data). CCDC 2469915.

Table S 13: Selected bond lengths (Å) and angles (°) for ***5**$^{m\text{-}xyl}$*

Al(1)-Al(1')	Al(1)-N(1)	Al(1)-N(2)	Al(1)-I(1)
2.5832(12)	1.9540(17)	1.9272(18)	2.5353(6)

Table S 14: Selected bond angles (°) for ***5**$^{m\text{-}xyl}$*

N(1)-Al(1)-N(2)	Al(1')-Al(1)-I(1)
68.82(7)	115.15(3)

Single crystal X-ray data for 6$^{m\text{-}xyl}$

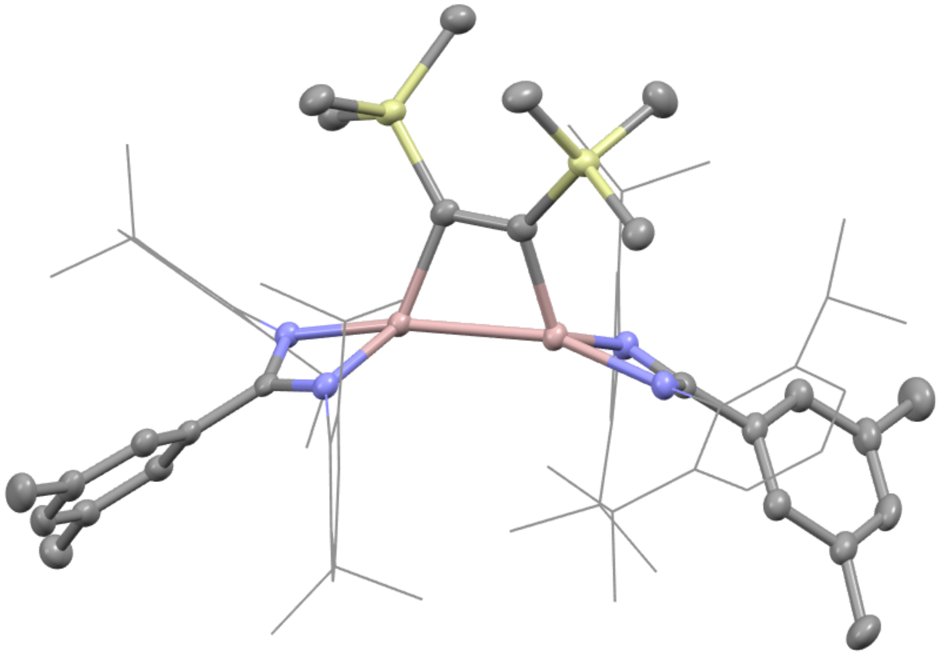

*Figure S 35: The solid-state structure of **6$^{m\text{-}xyl}$** (Solvent molecules and hydrogen atoms omitted, dipp groups wireframe for clarity, thermal ellipsoids 50% probability).*

Single crystals of **6$^{m\text{-}xyl}$** were grown from slow evaporation of a pentane solution. **6$^{m\text{-}xyl}$** was found to crystallise in the $P\bar{1}$ space group. The unit cell contained 3 pentane molecules per asymmetric unit, 1 of which was modelled using a solvent mask (SQUEEZE).

$C_{81.5}H_{122}Al_2N_4Si_2$ (M =1231.89 g/mol): triclinic, space group $P\bar{1}$ (no. 2), a = 12.1557(3) Å, b = 12.6838(3) Å, c = 26.0278(7) Å, α = 96.274(2)°, β = 96.202(2)°, γ = 92.214(2)°, V = 3960.43(17) Å^3, Z = 2, T = 100.00(10) K, μ(Mo Kα) = 0.108 mm^{-1}, D_{calc} = 1.033 g/cm^3, 49617 reflections measured (5.436° ≤ 2Θ ≤ 61.998°), 19497 unique (R_{int} = 0.0590, R_{sigma} = 0.0950) which were used in all calculations. The final R_1 was 0.0635 (I > 2σ(I)) and wR_2 was 0.1435 (all data). CCDC 2469916.

Table S 15: Selected bond lengths (Å) for ***6****$^{m\text{-}xyl}$*

**Al(1)-Al(2)**			
2.5462(9)			
**Al(1)-N(1)**	**Al(1)-N(2)**	**Al(2)-N(3)**	**Al(2)-N(4)**
1.9594(17))	1.9866(18)	1.9795(17)	1.9651(18)
**Al(1)-C(64)**	**Al(2)-C(65)**		
2.003(2)	2.002(2)		
**Si(1)-C(64)**	**Si(2)-C(65)**	**C(64)-C(65)**	
1.872(2)	1.871(2)	1.378(3)	

Table S 16: Selected bond angles and torsions (°) for ***6****$^{m\text{-}xyl}$*

**N(1)-Al(1)-N(2)**	**N(3)-Al(2)-N(4)**	
67.55(7)	67.27(7)	
**Al(1)-C(64)-C(65)**	**Al(2)-C(65)-C(64)**	**Al(1)-C(64)-C(65)-Al(2)**
104.8(1)	105.0(1)	24.6(2)

Single crystal X-ray data for 9*$^{m\text{-}xyl}$*

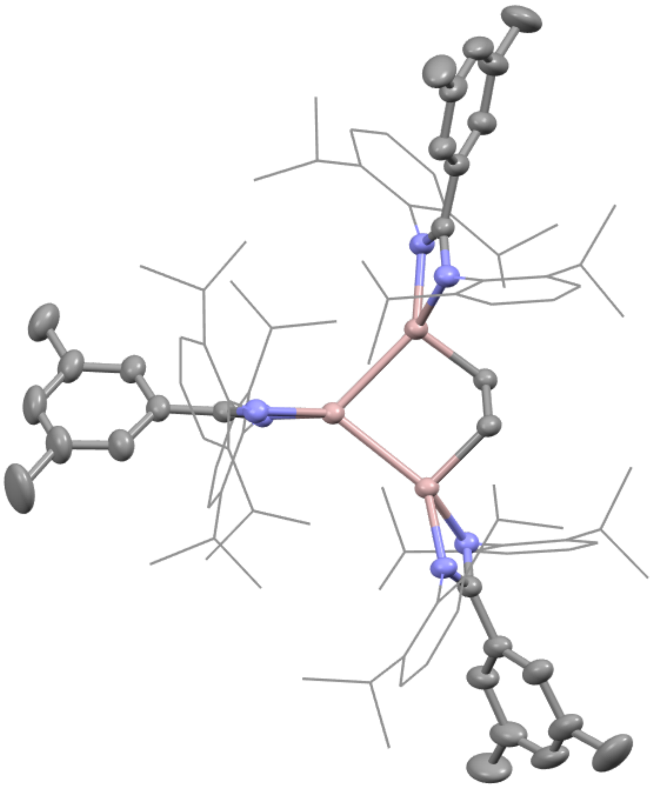

Figure S 36: The solid-state structure of ***9****$^{m\text{-}xyl}$ (Solvent molecules and hydrogen atoms omitted, dipp groups wireframe for clarity, thermal ellipsoids 50% probability).*

Single crystals of **9**$^{m\text{-}xyl}$ were grown from slow evaporation of a heptane solution. **9**$^{m\text{-}xyl}$ was found to crystallise in the $P2_1$/n space group. The unit cell contained 4 heptane molecules per asymmetric unit, which were modelled using a solvent mask (SQUEEZE).

$C_{108}H_{149}Al_3N_6$ (M =1612.26 g/mol): monoclinic, space group $P2_1$/n (no. 14), a = 15.05770(10) Å, b = 44.0378(3) Å, c = 15.49930(10) Å, β = 98.5230(10)°, V = 10164.21(12) Å^3, Z = 4, T = 150.03(10) K, μ(Cu Kα) = 0.689 mm^{-1}, D_{calc} = 1.054 g/cm^3, 276913 reflections measured (7.026° ≤ 2Θ ≤ 159.416°), 21278 unique (R_{int} = 0.0742, R_{sigma} = 0.0275) which were used in all calculations. The final R_1 was 0.0497 (I > 2σ(I)) and wR_2 was 0.1252 (all data). CCDC 2469917.

Table S 17: Selected bond lengths and distances (Å) for ***9**$^{m\text{-}xyl}$*

**Al(1)-Al(2)**	**Al(1)-Al(3)**	**Al(2)-Al(3)**			
2.6651(9)	2.6269(8)	3.5358(6)			
**Al(1)-N(1)**	**Al(1)-N(2)**	**Al(2)-N(3)**	**Al(2)-N(4)**	**Al(3)-N(5)**	**Al(3)-N(6)**
1.955(1)	1.983(2)	1.984(1)	1.976(1)	1.967(1)	1.971(1)
**Al(2)-C(100)**	**Al(3)-C(101)**	**C(100)-C(101)**			
1.984(2)	1.979(2)	1.557(2)			

Table S 18: Selected bond angles and torsions (°) for ***9**$^{m\text{-}xyl}$*

**Al(2)-Al(1)-Al(3)**	**N(1)-Al(1)-N(2)**	**N(3)-Al(2)-N(4)**	**N(5)-Al(3)-N(6)**
83.84(3)	67.65(6)	67.51(5)	67.63(6)
**Al(2)-C(100)-C(101)**	**Al(3)-C(101)-C(100)**	**Al(2)-C(100)-C(101)-Al(3)**	
105.49(9)	101.72(9)	81.43(9)	

Single crystal X-ray data for 10$^{m\text{-}xyl}$

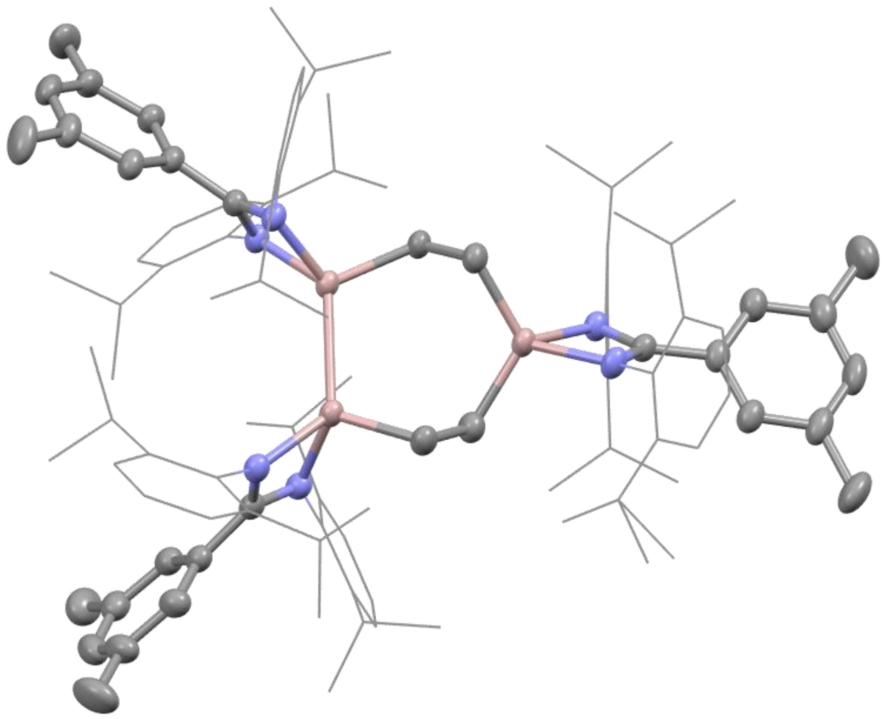

Figure S 37: The solid-state structure of ***10$^{m\text{-}xyl}$*** *(Solvent molecules and hydrogen atoms omitted, dipp groups wireframe for clarity, thermal ellipsoids 50% probability).*

Single crystals of **10$^{m\text{-}xyl}$** were grown from slow evaporation of a hexane solution. **10$^{m\text{-}xyl}$** was found to crystallise in the $P\bar{1}$ space group. The unit cell contained 3 hexane molecules per asymmetric unit, which were modelled using a solvent mask (SQUEEZE). C31-C33 and C31-C32 distances were restrained with sigma of 0.02.

$C_{112}H_{158}Al_3N_6$ (M =1669.37 g/mol): triclinic, space group $P\bar{1}$ (no. 2), a = 12.41140(10) Å, b = 20.3291(2) Å, c = 21.3439(3) Å, α = 82.6960(10)°, β = 77.6110(10)°, γ = 88.0250(10)°, V = 5217.10(10) Å^3, Z = 2, T = 150.00(10) K, μ(Cu Kα) = 0.685 mm^{-1}, D_{calc} = 1.063 g/cm^3, 145252 reflections measured (6.486° ≤ 2Θ ≤ 157.376°), 21690 unique (R_{int} = 0.0835, R_{sigma} = 0.0505) which were used in all calculations. The final R_1 was 0.0459 (I > 2σ(I)) and wR_2 was 0.136 (all data). CCDC 2469918.

Table S 19: Selected bond lengths (Å) and distances for ***10**$^{m\text{-}xyl}$*

**Al(1)-Al(2)**	**Al(1)-Al(3)**	**Al(2)-Al(3)**			
3.9982(6)	4.0162(6)	2.6070(5)			
**Al(1)-N(1)**	Al(1)-N(2)	**Al(2)-N(3)**	**Al(2)-N(4)**	**Al(3)-N(5)**	**Al(3)-N(6)**
1.950(1)	**1.956(1)**	1.987(1)	1.957(1)	1.955(1)	1.978(1)
**Al(1)-C(100)**	**Al(1)-C(102)**	**Al(2)-C(101)**	**Al(3)-C(103)**		
1.959(1)	1.960(2)	1.977(2)	1.972(2)		
**C(101)-C(100)**	**C(102)-C(103)**				
1.558(2)	1.556(2)				

Table S 20: Selected bond angles and torsions(°) for ***10**$^{m\text{-}xyl}$*

**N(1)-Al(1)-N(2)**	**N(3)-Al(2)-N(4)**	**N(5)-Al(3)-N(6)**	**C(100)-Al(1)-C(102)**
68.33(5)	67.77(5)	67.77(5)	119.51(6)
**C(101)-Al(2)-Al(3)**	**C(103)-Al(3)-Al(2)**	**Al(1)-C(100)-C(101)-Al(2)**	**Al(3)-C(102)-C(103)-Al(4)**
113.40(5)	111.06(5)	90.5(1)	102.40(9)

Single crystal X-ray data for 11$^{p\text{-}tol}$

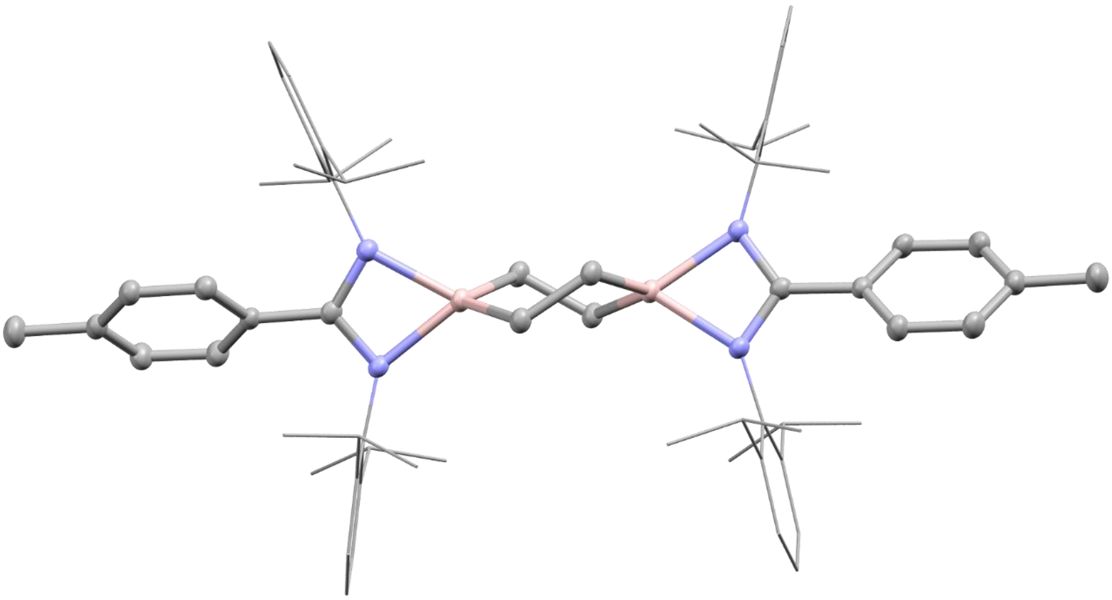

*Figure S 38: The solid-state structure of **11$^{p\text{-}tol}$** (Hydrogen atoms omitted, dipp groups wireframe for clarity, thermal ellipsoids 50% probability).*

Single crystals of **11$^{p\text{-}tol}$** were grown from slow evaporation of a saturated hexane solution. **11$^{p\text{-}tol}$** was found to crystallise in the $P2_1/c$ space group. The unit cell contained 1 molecule of hexane per asymmetric unit.

$C_{74}H_{104}Al_2N_4$ (M =1103.57 g/mol): monoclinic, space group $P2_1/c$ (no. 14), a = 18.5661(3) Å, b = 18.1347(4) Å, c = 20.7132(4) Å, β = 105.034(2)°, V = 6735.2(2) Å^3, Z = 4, T = 100.00(10) K, μ(Cu Kα) = 0.704 mm^{-1}, $Dcalc$ = 1.088 g/cm^3, 65885 reflections measured (4.928° ≤ 2Θ ≤ 152.624°), 13499 unique (R_{int} = 0.0921, R_{sigma} = 0.0651) which were used in all calculations. The final R_1 was 0.0727 (I > 2σ(I)) and wR_2 was 0.2116 (all data). CCDC 2503435.

*Table S 21: Selected bond lengths and distances (Å) for **11$^{p\text{-}tol}$***

**Al(1)-Al(2)**	**C1-C2**	**C3-C4**	
3.471(1)	1.549(3)	1.553(4)	
**Al(1)-N(1)**	**Al(1)-N(2)**	**Al(2)-N(3)**	**Al(2)-N(4)**
1.954(2)	1.936(2)	1.947(2)	1.945(2)

*Table S 22: Selected bond angles and torsions (°) for **11$^{p\text{-}tol}$***

**N(1)-Al(1)-N(2)**	**N(3)-Al(1)-N(4)**	**C(1)-Al(1)-C(3)**	**C(2)-Al(2)-C(4)**
68.66(8)	68.61(8)	114.0(1)	113.7(1)
**Al(1)-C(1)-C(2)-Al(2)**	**Al(1)-C(3)-C(4)-Al(2)**		
-59.8(2)	-58.5(2)		

Single crystal X-ray data for 11$^{m\text{-}xyl}$

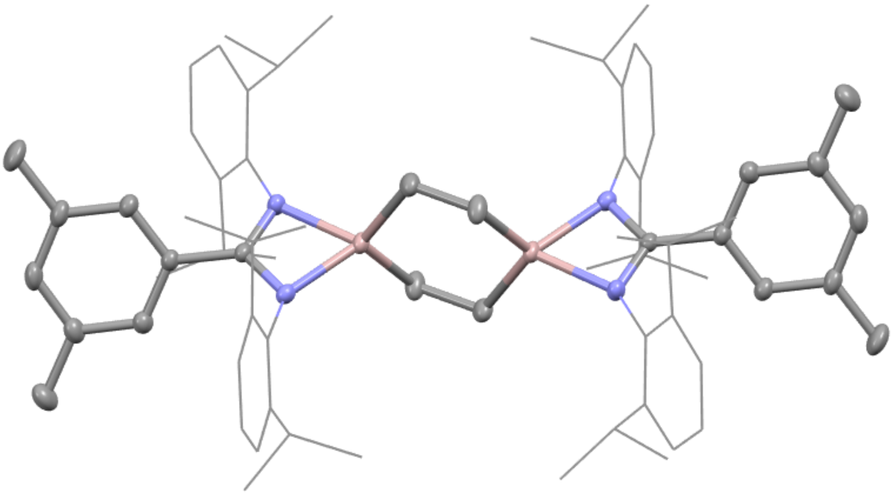

*Figure S 39: The solid-state structure of **11**$^{m\text{-}xyl}$ (Hydrogen atoms omitted, dipp groups wireframe for clarity, thermal ellipsoids 50% probability).*

Single crystals of **11**$^{m\text{-}xyl}$ were grown from the slow cooling of a saturated pentane solution. **11**$^{m\text{-}xyl}$ was found to crystallise in the $P\overline{1}$ space group, with half a molecule in the asymmetric unit. The unit cell contained 1.33 pentane molecules per asymmetric unit, which were modelled using a solvent mask (SQUEEZE).

$C_{76.6}H_{109.84}Al_2N_4$ (M =1140.68 g/mol): triclinic, space group $P\overline{1}$ (no. 2), a = 9.8050(3) Å, b = 12.5676(2) Å, c = 16.3553(3) Å, α = 99.862(2)°, β = 104.271(2)°, γ = 112.074(2)°, V = 1730.11(7) Å^3, Z = 1, T = 100.0(4) K, μ(Mo Kα) = 0.086 mm^{-1}, D_{calc} = 1.095 g/cm^3, 77013 reflections measured (4.566° ≤ 2Θ ≤ 70.202°), 15334 unique (R_{int} = 0.0534, R_{sigma} = 0.0422) which were used in all calculations. The final R_1 was 0.0676 (I > 2σ(I)) and wR_2 was 0.2060 (all data). CCDC 2469919.

*Table S 23: Selected bond lengths and distances (Å) for **11**$^{m\text{-}xyl}$*

**Al(1)-Al(1')**	**Al(1)-N(1)**	**Al(1)-N(2)**
3.4711(4)	1.954(1)	1.9501(8)
**Al(1)-C(35)**	**Al(1)-C(34)**	**C(34)-C(35)**
1.967(2)	1.961(2)	1.548(1)

*Table S 24: Selected bond angles and torsions (°) for **11**$^{m\text{-}xyl}$*

**N(1)-Al(1)-N(2)**	**C(34)-Al(1)-C(35)**	**Al(1)-C(35)-C(34)-Al(1')**
68.44(4)	115.01(6)	-42.2(1)

Preliminary single crystal X-ray data for 11$^{m\text{-}xyl}$-2

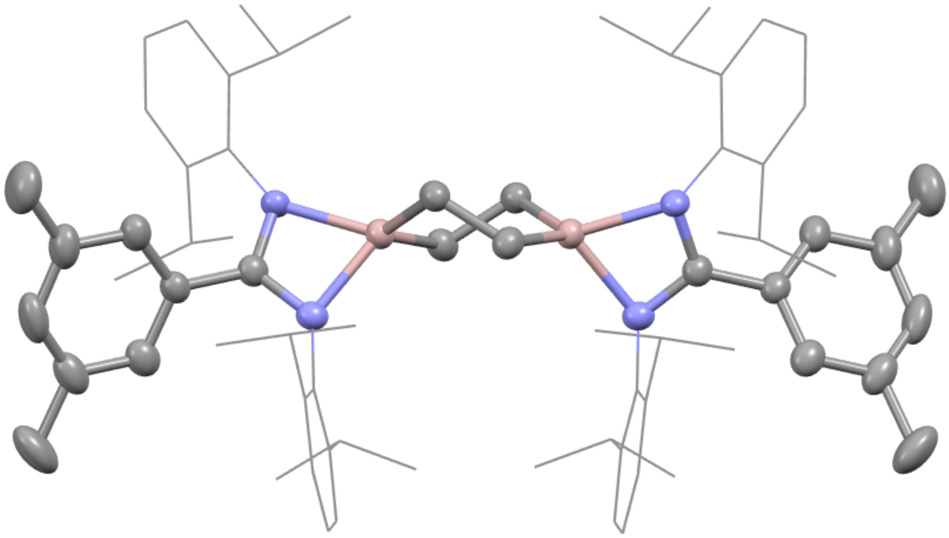

*Figure S 40: The solid-state structure of **11$^{m\text{-}xyl}$-2** (Hydrogen atoms omitted, dipp groups wireframe for clarity, thermal ellipsoids 50% probability).*

Single crystals of **11$^{m\text{-}xyl}$-2** were obtained from the slow evaporation of a pentane solution. This is preliminary data, where only connectivity was obtained.
$C_{35}H_{47}AlN_2$ (M =522.72 g/mol): monoclinic, space group $C2/c$ (no. 15), a = 15.8101(5) Å, b = 12.9113(4) Å, c = 32.4070(10) Å, β = 96.689(3)°, V = 6570.2(4) Å^3, Z = 8, T = 149.99(10) K, μ(Cu Kα) = 0.699 mm^{-1}, D_{calc} = 1.057 g/cm^3, 6491 reflections measured (13.62° ≤ 2Θ ≤ 113.854°), 3848 unique (R_{int} = 0.0377, R_{sigma} = 0.0644) which were used in all calculations. The final R_1 was 0.0475 (I > 2σ(I)) and wR_2 was 0.1268 (all data).

Reply for B-level Check CIF alerts for each compound

Complex 2^p-tol^-1 – second polymorph

PLAT910_ALERT_3_B Missing FCF Reflection(s) Below Theta(Min)[Deg]= 3.54 Note
Response: This is a good quality experimental dataset from a crystal structure collected with detector distance of 40 mm. A solvent mask (SQUEEZE) was used to omit the electron density of 1.9 and 1.6 hexanes in two voids per unit cell, but this does not seem to be the source of the problem as the reflections were still missing when we refined the structure without the solvent mask. The missing reflections don't affect the quality of the final crystal structure model.

Complex 2^m-xyl^ *– first polymorph*

PLAT910_ALERT_3_B Missing FCF Reflection(s) Below Theta(Min)[Deg]= 2.69 Note
Response: This is a good quality experimental dataset from a crystal structure collected with detector distance of 38 mm. The only solvent present in the structure (toluene) was modelled and a solvent mask was not used. Only two reflections were omitted deliberately as they had |Error/esd| higher than 10. The missing reflections don't affect the quality of the final crystal structure model.

Complex 6^m-xyl^

PLAT910_ALERT_3_B Missing FCF Reflection(s) Below Theta(Min)[Deg]= 2.72 Note
Response: This is a good quality experimental dataset from a crystal structure collected with detector distance of 38 mm. A solvent mask (SQUEEZE) was used to omit the electron density of 1.5 molecules of pentane in one void per asymmetric unit, but this does not seem to be the source of the problem as the reflections were still missing when we refined the structure without the solvent mask. The missing reflections don't affect the quality of the final crystal structure model.

5. Computational analysis

All DFT calculations were performed using the Gaussian 9 (Revision E.01), 16 (Revision C.01) and ORCA (versions 6.0.1 and 6.1.0)[15] program packages as compiled on King's College London's CREATE and the Meluxina High Performance Computing (HPC) clusters.[16,17] Initial coordinates were obtained from the relevant SC-XRD structures. All geometries were optimised without symmetry constraints at the M06-2X-D3 level of theory, including Grimme's atom-pairwise correction to account for dispersion effects. For geometry optimisations of the full system, Ahlrichs' def2-TZVP basis set was used for all metal centres and donor atoms directly bound to metal centres in conjunction with the def2-SVP basis set placed on the remaining centres (C, H). Geometry optimisations of stationary points (intermediates and transition states) along the reaction coordinate were carried out with a def2-SVP basis set on all atoms. An ultrafine integration grid corresponding to a pruned grid of 99 radial shells and 590 angular points per shell was employed to ensure highly accurate numerical integration of the electron density. The same procedure was used for literature compounds. In all cases, ground state and transition state geometries were confirmed by way of frequency calculation at the same level of theory to indicate the absence or presence of one negative eigenvalues in the Hessian matrix, respectively. All subsequent single point corrections and analyses were performed at the SMD-M06-2X-D3/def2-TZVP level of theory, using the SMD solvation model by Truhlar, Cramer and Marenich, with solvent parameters corresponding to those of cyclohexane (ε=2.0165).[18] For mechanistic calculations, the theory level was M06-2X/def2-svp, employing the above solvation model for single point corrections.

Time dependent (TD) DFT calculations were carried out at the SMD-M06-2X-D3/def2-TZVP (cyclohexane, ε=2.0165) level of theory, employing the Tamm-Dancoff approximation (TDA). Natural Bond Orbital analysis was conducted using the NBO program (Version 3.1).[19] Quantum Theory of Atoms in Molecules (QTAIM) analysis was conducted using the AIMAll package, from the Gaussian obtained-wavefunction file.[20] Isosurface mapping of the Electron Localization Function (ELF) was performed using the Multiwfn package, employing the high quality grid preset – covering the whole system, about 1,728,000 points in total.[21,22] Orbital localisations employed the Intrinsic Atomic Orbitals and Intrinsic Bond Orbitals (IAO-IBO) algorithm as implemented in ORCA.[23] The calculations utilised the M06-2X-D3 functional in conjunction with the def2-TZVP basis set. The RIJCOSX approximation for Coulomb and HF exchange integrals was utilised to speed-up the calculations together with corresponding auxiliary basis sets (*autoaux* keyword).[24] Chemical bonding was further analysed within the EDA-NOCV framework,[25] combining the Extended Transition State (ETS) energy decomposition method with the Natural Orbitals for Chemical Valence (NOCV).[26] Figures of all structures and isosurface plots were generated with ChemCraft (Version 1.8) and UCSF ChimeraX (Version 1.61). [27,28]

Functional benchmarking

Table S 25: Functional data table for the parametrisation process of $\mathbf{2^{p\text{-}tol}}$

	Al_1-Al_2 (Å)	Al_1-Al_3 (Å)	Al_2-Al_3 (Å)	ϑAl_1 (°)	ϑAl_2 (°)	ϑAl_3 (°)	UV-Vis (nm)
	2.6640(12)	2.6603(10)	2.6553(11)	59.83(3)	60.02(3)	60.15(3)	316, 450
$2^{p\text{-}tol}$	2.6573(10)	2.6309(11)	2.6528(12)	60.22(3)	59.40(3)	60.38(3)	
	2.6621(11)	2.6612(10)	2.6477(10)	59.75(3)	59.83(3)	60.42(3)	
	2.6594(10)	2.6416(11)	2.6436(10)	60.16(3)	59.65(3)	60.19(3)	
B3LYP	2.73576	2.73107	2.73046	59.928	59.95	60.121	532
	2.59856	2.65093	2.60747	59.555	61.221	59.224	570
M06L	2.60192	2.6447	2.59912	59.384	61.127	59.489	656
	2.5901	2.63721	2.59109	59.422	61.194	59.385	660
M062X	2.64889	2.64098	2.62925	59.608	60.047	60.346	332, 428
	2.64029	2.6356	2.62059	59.565	60.128	60.307	335, 429
ωB97X	2.64383	2.64956	2.64781	60.02774	60.09345	59.87881	293, 333
ωB97XD	2.58613	2.63767	2.5958	59.583	61.196	59.221	

= Dispersion corrected (GD3, unless integrated)

Given the complexity of the electronic structure, ensuring functional best fit was a priority before conducting in depth analysis. The fitting of four common functionals: B3LYP, ωB97XD, M06-L and M06-2x was tested against experimental geometry values obtained from two separate single crystal structures (four units) and UV-Vis spectroscopic parameters.

The M06-2x-D3 functional was selected for the remainder of the analysis work based on its excellent adherence to the crystallographically determined geometries and mapping of the key TD-DFT transition bands seen *via* UV-Vis spectroscopy. All other functionals failed to accurately determine reasonable transition wavelengths and resolve both experimentally observed bands.

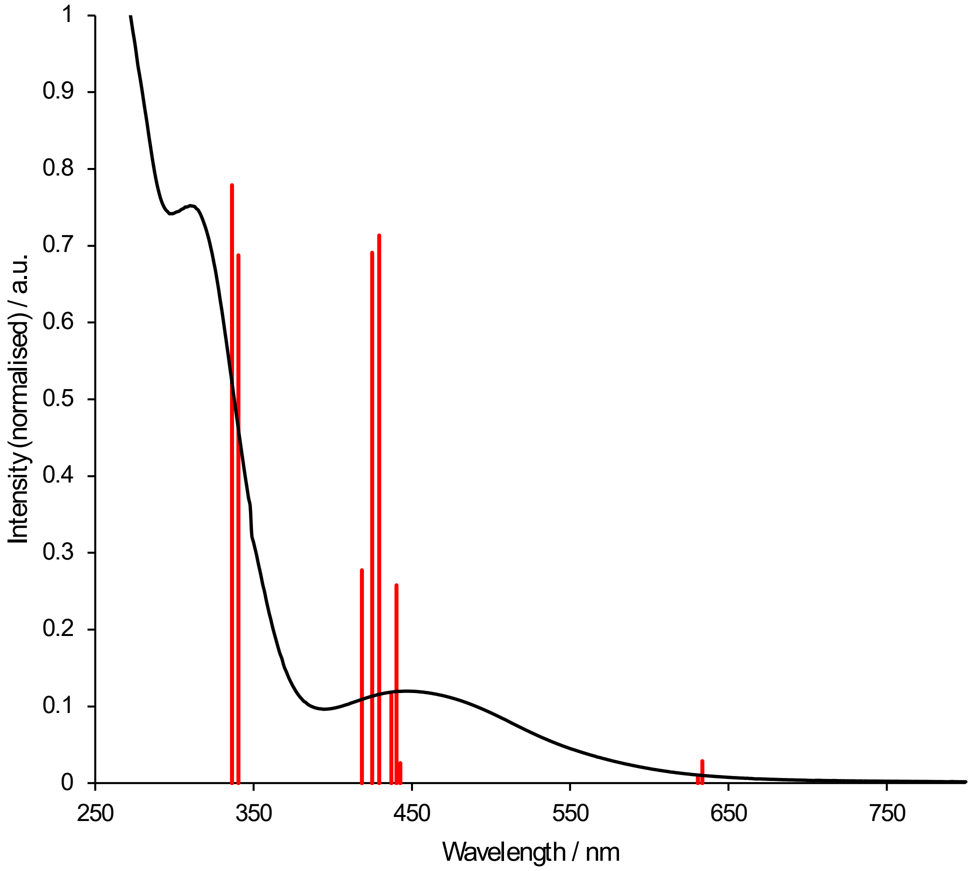

*Figure S 41: Simulated M06-2X TD-DFT transition bands of **2**$^{p\text{-}tol}$ overlaid with experimental UV-vis of **2**$^{p\text{-}tol}$ in cyclohexane*

Time dependent-DFT – SMD(cyclohex)-M06-2X-GD3|def2-tzvp||M06-2X-GD3|def2-tzvp(Al,N)/def2-svp(C,H)

Table S 26: Key M06-2X TD-DFT transition bands of **2**$^{p\text{-}tol}$*, transitions with f < 0.2 have been excluded.*

Key TD-DFT Bands					
**Excited State 6:**					
**Ground**	**Excited**	**E (eV)**	**λ (nm)**	**f**	**S^2**
HOMO-1	LUMO	2.8875	429.39	0.2854	0.000
	LUMO+1				
	LUMO+2				
	LUMO+3				
HOMO	LUMO+1				
	LUMO+2				
**Excited State 7:**					
HOMO-1	LUMO +2	2.9171	425.03	0.2763	0.000
	LUMO +3				
HOMO	LUMO				
	LUMO +1				
	LUMO +2				
	LUMO +3				
**Excited State 9:**					
HOMO	LUMO+6	3.6415	340.48	0.2750	0.000
	LUMO+7				
	LUMO+8				
	LUMO+19				
**Excited State 10:**					
HOMO-1	LUMO+6	3.6861	336.35	0.3116	0.000
	LUMO+7				
	LUMO+8				
	LUMO+18				
	LUMO+19				

Table S 27: Key M06-2X TD-DFT transition bands of ***2***$^{m\text{-}xyl}$*, transitions with f < 0.2 have been excluded.*

Key TD-DFT Bands					
**Excited State 6:**					
**Ground**	**Excited**	**E (eV)**	**λ (nm)**	**f**	**S^2**
HOMO-1	LUMO	2.8936	428.47	0.2724	0.000
	LUMO+1				
	LUMO+2				
	LUMO+3				
HOMO	LUMO+1				
	LUMO+2				
**Excited State 7:**					
HOMO-1	LUMO +1	2.9241	424.01	0.2639	0.000
	LUMO +2				
	LUMO +3				
HOMO	LUMO				
	LUMO +1				
	LUMO +2				
	LUMO +3				
**Excited State 9:**					
HOMO	LUMO+4	3.6415	340.47	0.2800	0.000
	LUMO+5				
	LUMO+7				
	LUMO+8				
	LUMO+14				
	LUMO+19				
**Excited State 10:**					
HOMO-1	LUMO+4	3.6866	336.31	0.3127	0.000
	LUMO+5				
	LUMO+7				
	LUMO+8				
	LUMO+19				

Electron localisation function - SMD(cyclohex)-M06-2X-GD3|def2-tzvp||M06-2X-GD3|def2-tzvp(Al,N)/def2-svp(C,H)

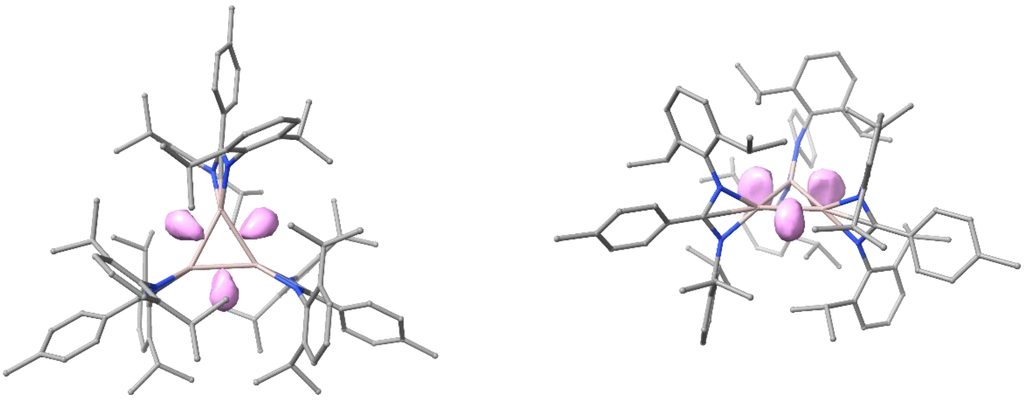

Figure S 42: Isosurfaces for the ELF of **2**$^{p\text{-}tol}$ *(Isovalue: 0.92), highlighting the localisation of the Al-Al bonding pairs to lie outside the interatomic plane.*

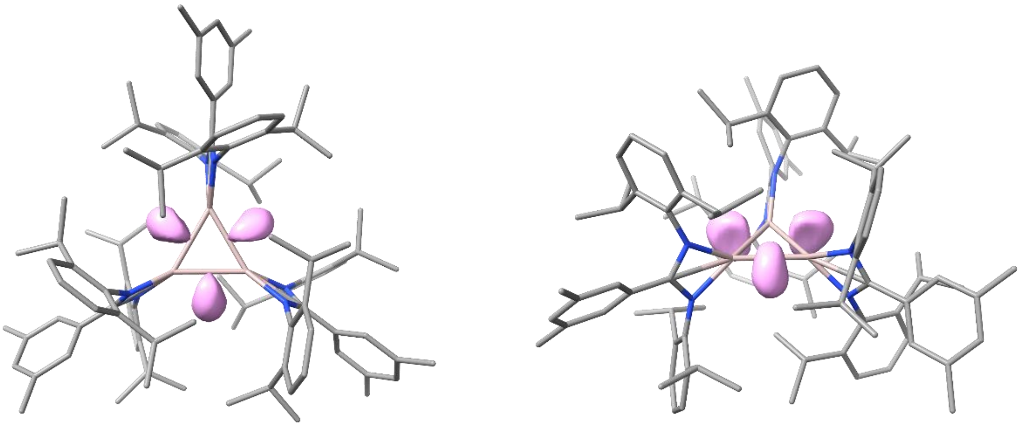

Figure S 43: Isosurfaces for the ELF of **2**$^{m\text{-}xyl}$ *(Isovalue: 0.92), highlighting the localisation of the Al-Al bonding pairs to lie outside the interatomic plane.*

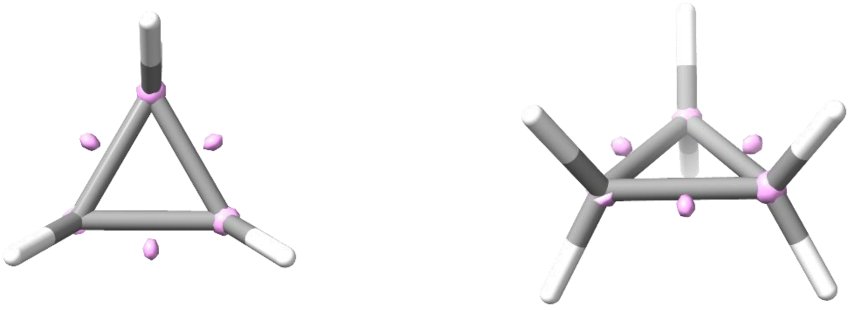

Figure S 44: Isosurfaces for the ELF of cyclopropane (Isovalue: 0.92), indicating much smaller areas of electron localisation outside the C-C plane compared to **2**$^{m\text{-}xyl/p\text{-}tol}$.

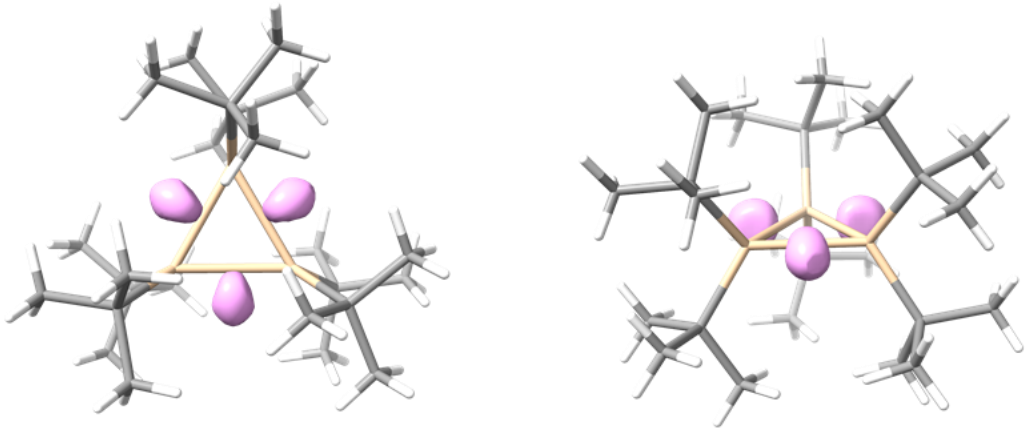

Figure S 45: Isosurfaces for the ELF of for cyclotrisilane (Isovalue: 0.92), indicating the localisation of C-C bonding pairs to also lie outside the C-C plane similar to $\mathbf{2}^{m\text{-}xyl/p\text{-}tol}$.

Energy Decomposition Analysis / Natural Orbitals for Chemical Valence (ETS-NOCV)

Energy Decomposition Analysis (Table S 28) reveals an overall bond energy of ΔE_{int} = –87.0 kcal mol^{-1} between the AlL and Al_2L_2 fragments. Orbital (ΔE_{orb} = –246.3 kcal mol^{-1}) and electrostatic terms ($\Delta E_{\mathrm{elstat}}$ = –399.8 kcal mol^{-1}) make major contributions to the total bonding interaction, largely overcompensating the repulsive Pauli term ($\Delta E_{\mathrm{Pauli}}$ = 568.5 kcal mol, Table S28). The dispersion term is comparatively small yet noticeable (ΔE_{disp} = –9 kcal mol^{-1}), indicating that the present ligand supplies less dispersion energy than, for example, the 2,4,5-triscyclopentylphenyl ligand used to stabilise the cyclotristannane reported by Power and co-workers. According to ETS-NOCV analysis, the principal electron flow occurs from each Al(I) centre towards the Al–Al σ-bonds, with hyperconjugation from opposite Al_2 fragments further enhancing the interaction with the Al centre (Figs. S45-46). In the truncated C_3-symmetric model ($\mathbf{2}^{ph}$, Ar = phenyl), the interaction energy is expectedly reduced in magnitude (ΔE_{int} = –69.4 kcal mol^{-1}). Nonetheless, the relative contributions from the orbital (ΔE_{orb} = –229.2 kcal mol^{-1}) and electrostatic terms ($\Delta E_{\mathrm{elstat}}$ = –348.3 kcal mol^{-1}) remain unchanged compared to $\mathbf{2}^{p\text{-}tol}$. In the absence of the alkyl substituents on the aromatic rings, the dispersion contribution is diminished to ΔE_{disp} = –2.6 kcal mol^{-1}.

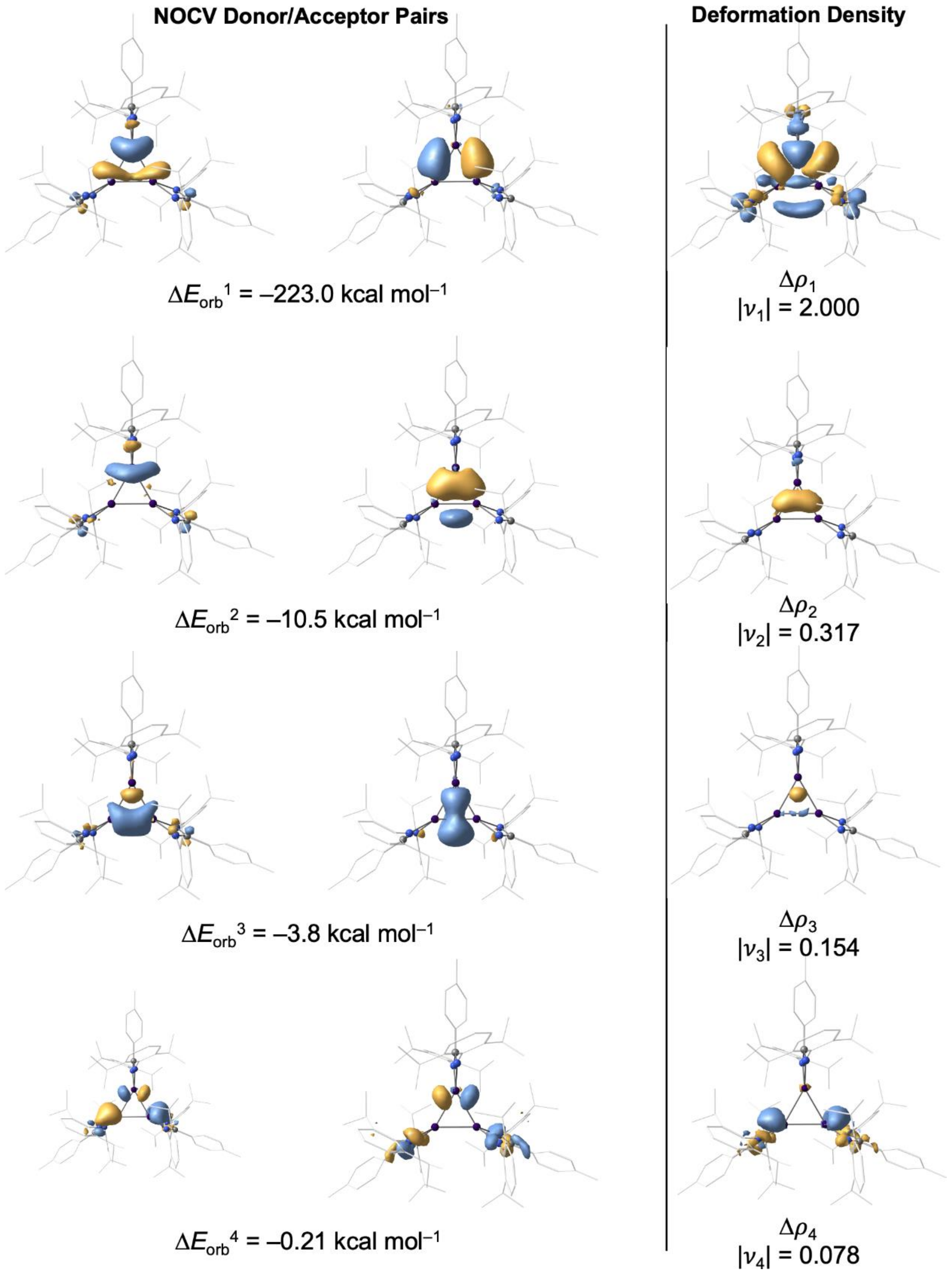

Figure S 46*: First four ETS-NOCV donor/acceptor orbital pairs and associated deformation densities for* ***2**$^{p\text{-}tol}$. Blue and gold deformation density surfaces indicate regions of charge depletion and accumulation, respectively.*

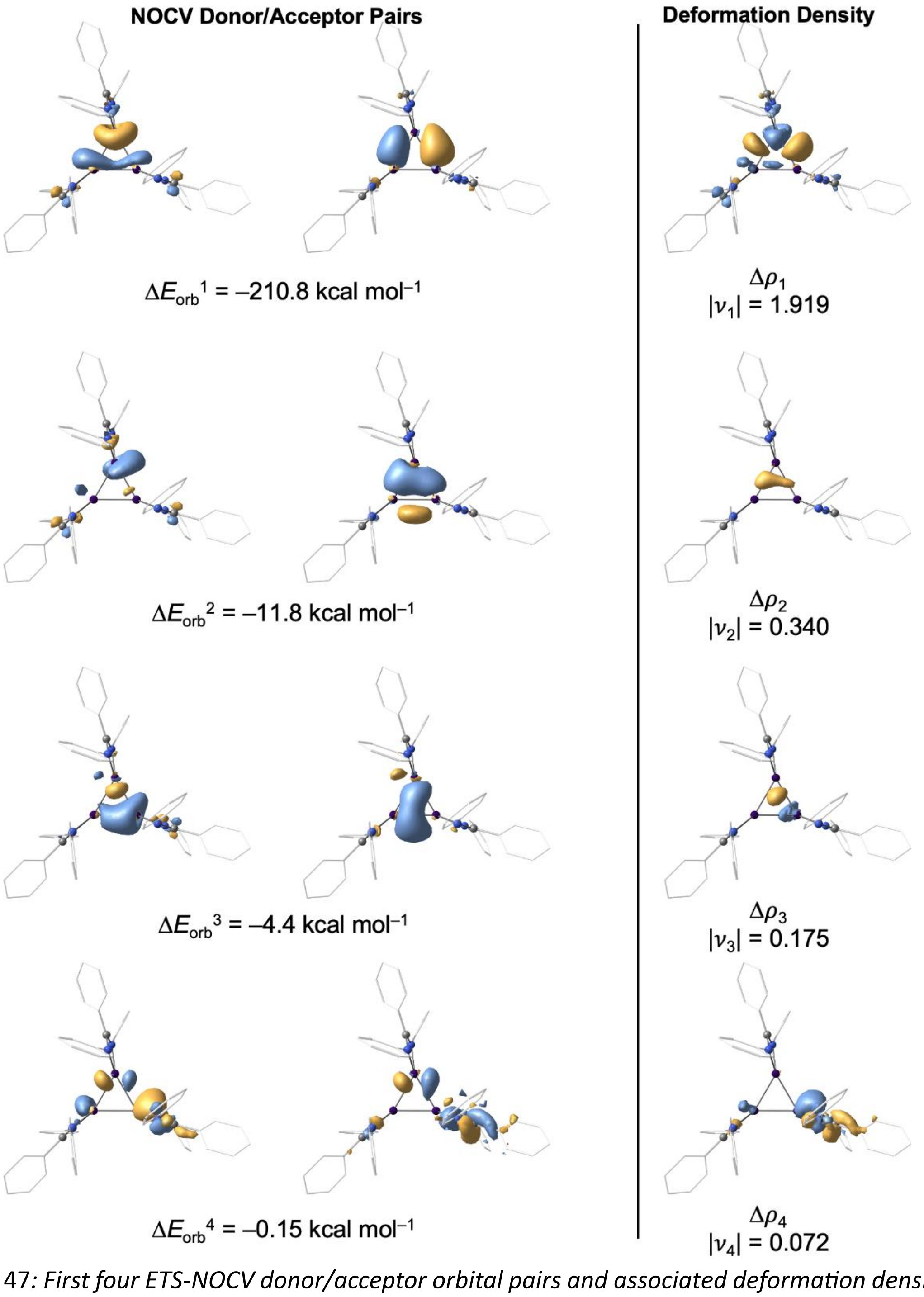

*Figure S 47: First four ETS-NOCV donor/acceptor orbital pairs and associated deformation densities for truncated C_3-symmetric model of **2**$^{p\text{-}tol}$. Blue and gold deformation density surfaces indicate regions of charge depletion and accumulation, respectively.*

Table S 28: Summary of ETS analysis for ***2****$^{p\text{-}tol}$.and a truncated model with C_3-symmetry. ^{a}Total bonding energy = E_{orb} + E_{elstat} + E_{disp}.*

	**2**$^{p\text{-}tol}$	**2**^{ph}**, Ar = phenyl (*C_3*)**
ΔE_{int} (interaction energy)	–87.01	–69.37
ΔE_{orb} (orbital energy)	–246.27 (38%)	–229.17 (39%)
ΔE_{Pauli} (Pauli repulsion)	+568.52	+510.92
ΔE_{elstat} (electrostatic energy)	–399.80 (61%)	–348.28 (60%)
ΔE_{disp} (dispersion energy)	–9.10 (1%)	–2.63 (1%)
Total bonding energy[a]	–655.17 (100%)	–580.08 (100%)

Quantum theory of atoms in molecules - SMD(cyclohex)-M06-2X-GD3|def2-tzvp||M06-2X-GD3|def2-tzvp(Al,N)/def2-svp(C,H)

The associated parameters at the Al–Al bcps are close to one another, reflecting only slight deviation from ideal local C_3 symmetry in the Al_3 bonding motif (ρ_{bcp} [Al_1-Al_2]: 0.0492 e/a_0^3; ρ_{bcp}[Al_1-Al_3]: 0.0495 e/a_0^3, ρ_{bcp}[Al_2-Al_3]: 0.0504 e/a_0^3). The values for the Laplacian of ρ ($\nabla^2\rho_{bcp}$) all lie between 0.0592 e/a_0^5 and 0.0559 e/a_0^5. The equivalent values for cyclopropane are approximately 0.241 e/a_0^3 and –0.461 e/a_0^5 for ρ_{bcp} and $\nabla^2\rho_{bcp}$, respectively, indicating a far lesser degree of covalency shared between the Al_3 core compared to the more classical system (Fig. S50). In each case, the bcps are not equidistant along the Al–Al bond paths, but rather slightly displaced from the geometric bond centre in a concentric arrangement through the three Al–Al bonds. The ring paths between the rcp and bcps are symmetrically convex, consistent with the small irregularity in the electron density distribution of the Al_3 core. The system also reveals significant ring strain, as evidenced by the curved bond paths (bp) between the Al nuclear attractors (deviation of the bp lengths from the geometric Al–Al distance ~0.066 a_0, cf. cyclopropane 0.0066 a_0). This data contrasts with that of the related molecules, where in both cases the bcps are centred on the midpoint of the bond and the rcp-to-bcp ring paths are straight. The system also reveals significant ring strain, as evidenced by the curved bond paths (bp) between the Al nuclear attractors (deviation of the bp lengths from the geometric Al–Al distance ~0.066 a_0, cf. cyclopropane 0.0066 a_0). Again, this data contrasts with that of the related molecules, where in both cases the bcps are centred on the midpoint of the bond and the rcp-to-bcp ring paths are straight.

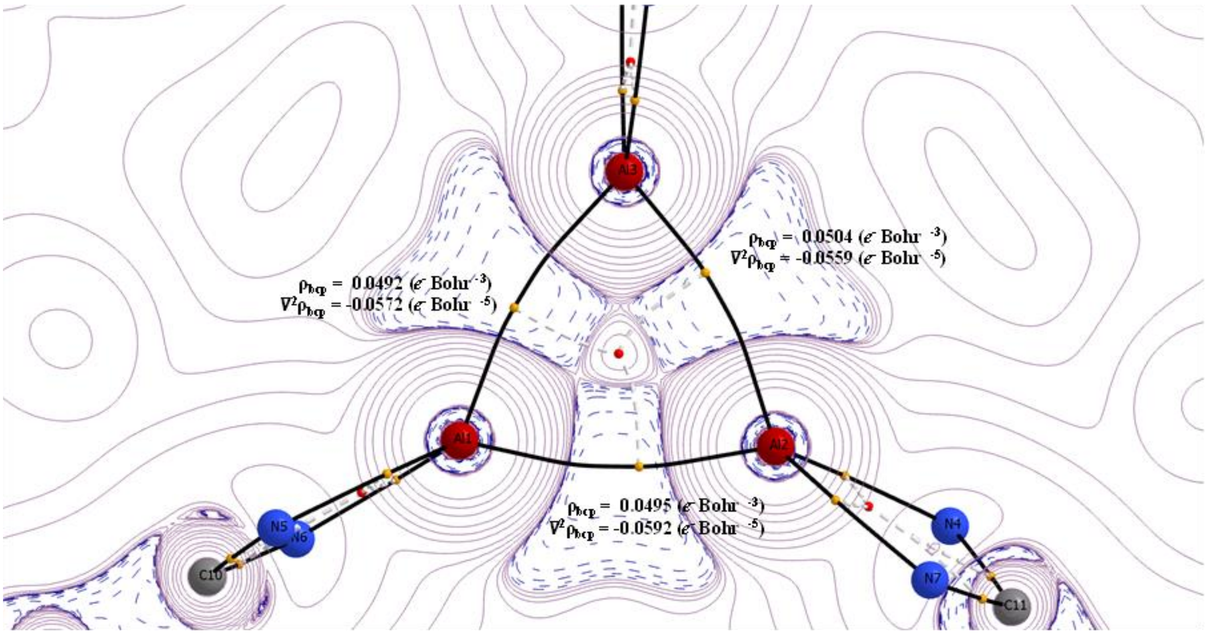

*Figure S 48: QTAIM topology plot for the Laplacian of the electron density ($\nabla^2\rho$) of **2**$^{p\text{-}tol}$, highlighting areas of charge concentration (blue) and depletion (pink). The values of electron density (ρ) and $\nabla^2\rho$ are indicated at each bcp.*

*Table S 29: Data table for the QTAIM analysis of **2**$^{p\text{-}tol}$, indicating the electronic parameters found at the bcps as well as strain indicators (BPL-GBL_I) and rcp properties. Cremer and Kraka energy density parameters (V,G,K,H) are also presented.*

	Al_1-Al_2	Al_1-Al_3	Al_2-Al_3
**ρ_{bcp} (e^- Bohr^{-3})**	0.0492	0.0495	0.0504
**$\nabla^2\rho_{bcp}$ (e^- Bohr^{-5})**	-0.0572	-0.0592	-0.0559
**BPL**	5.0561	5.0477	5.0183
**GBL_I**	4.9899	4.9806	4.9521
**BPL-GBL_I**	0.0662	0.0671	0.0662
**V**	-0.0242	-0.0234	-0.0273
**G**	0.0050	0.0043	0.0067
**K**	0.0192	0.0191	0.0206
**H**	-0.0192	-0.0191	-0.0206
**ρ_{rcp} (e^- Bohr^{-3})**		0.0352	
**$\nabla^2\rho_{rcp}$ (e^- Bohr^{-5})**		0.0108	

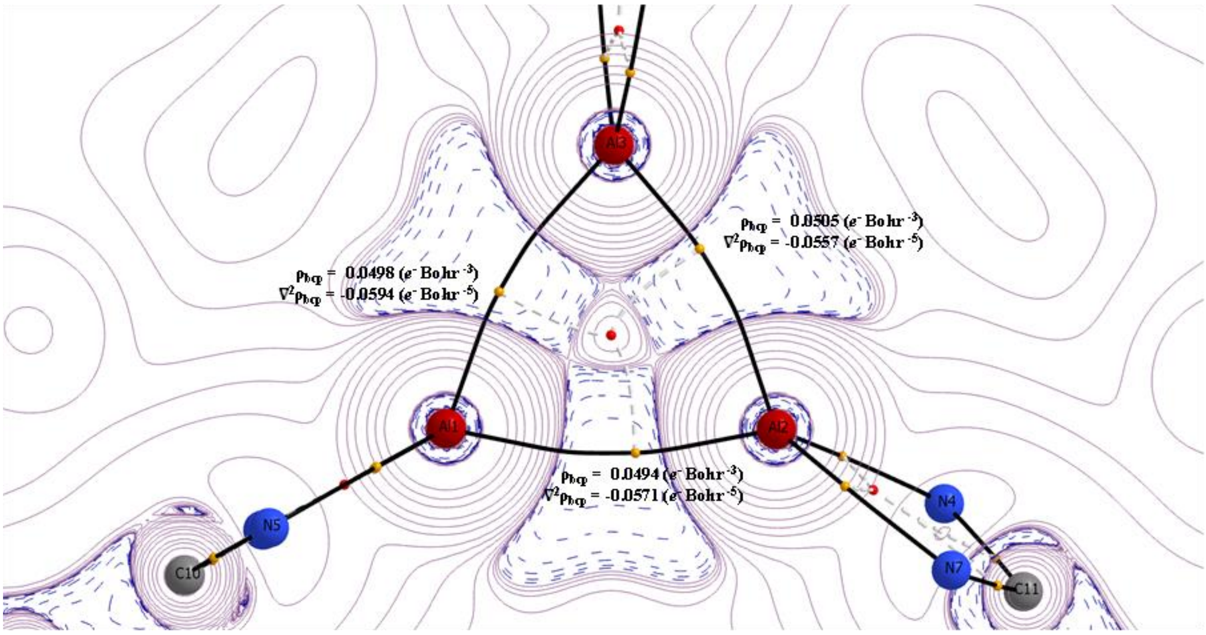

Figure S 49: QTAIM topology plot for the Laplacian of the electron density ($\nabla^2\rho$) of ***2****$^{m\text{-}xyl}$, highlighting areas of charge concentration (blue) and depletion (pink). The values of electron density (ρ) and $\nabla^2\rho$ are indicated at each bcp.*

Table S 30: Data table for the QTAIM analysis of ***2****$^{m\text{-}xyl}$, indicating the electronic parameters found at the bcps as well as strain indicators (BPL-GBL_I) and rcp properties. Cremer and Kraka energy density parameters (V,G,K,H) are also presented.*

	**Al_1-Al_2**	**Al_1-Al_3**	**Al_2-Al_3**
**ρ_{bcp} (e^- Bohr^{-3})**	0.0494	0.0498	0.0505
**$\nabla^2\rho_{bcp}$ (e^- Bohr^{-5})**	-0.0571	-0.0594	-0.0557
**BPL**	5.0492	5.0388	5.0127
**GBL_I**	4.9832	4.9719	4.9465
**BPL-GBL_I**	0.0661	0.0669	0.0662
**V**	-0.0246	-0.0241	-0.0276
**G**	0.0052	0.0046	0.0068
**K**	0.0194	0.0195	0.0208
**H**	-0.0194	-0.0195	-0.0208
**ρ_{rcp} (e^- Bohr^{-3})**		0.0354	
**$\nabla^2\rho_{rcp}$ (e^- Bohr^{-5})**		0.0105	

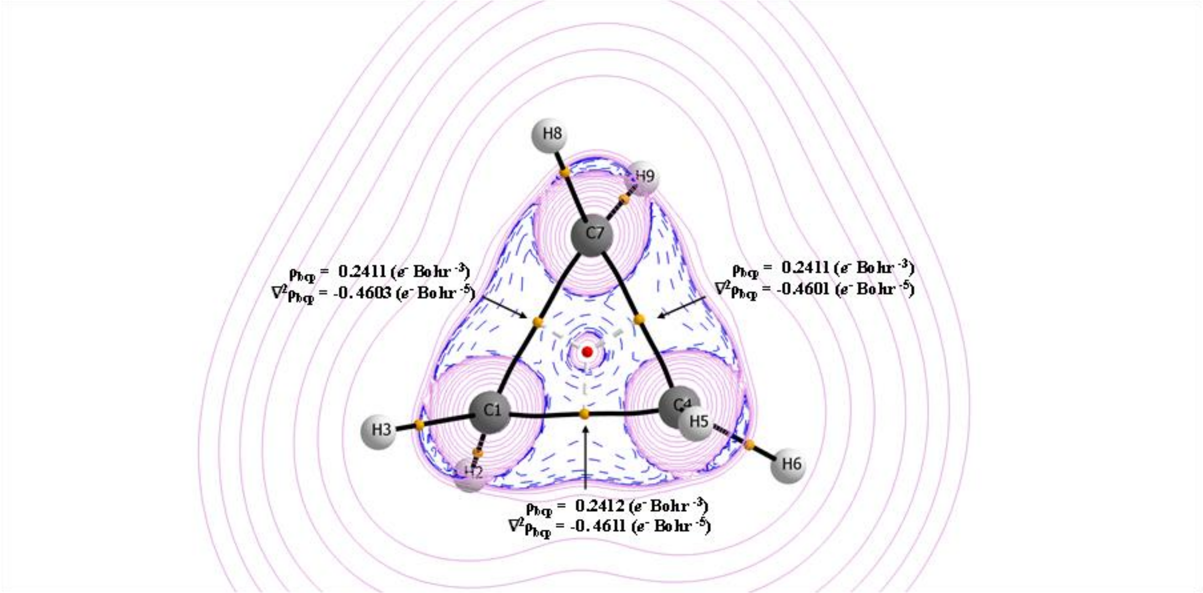

Figure S 50: QTAIM topology plot for the Laplacian of the electron density ($\nabla^2\rho$) of cyclopropane, highlighting areas of charge concentration (blue) and depletion (pink). The values of electron density (ρ) and $\nabla^2\rho$ are indicated at each bcp.

Table S 31: Data table for the QTAIM analysis of cyclopropane, indicating the electronic parameters found at the bcps as well as strain indicators (BPL-GBL_I) and rcp properties. Cremer and Kraka energy density parameters (V,G,K,H) are also presented.

	C_1-C_4	C_1-C_7	C_4-C_7
**ρ_{bcp} (e^- Bohr^{-3})**	0.2412	0.2411	0.2411
**$\nabla^2\rho_{bcp}$ (e^- Bohr^{-5})**	-0.4611	-0.4603	-0.4601
**BPL**	2.8373	2.8378	2.8379
**GBL_I**	2.8307	2.8312	2.8313
**BPL-GBL_I**	0.0066	0.0066	0.0066
**V**	-0.3081	-0.3079	-0.3078
**G**	0.0964	0.0964	0.0964
**K**	0.2117	0.2115	0.2114
**H**	-0.2117	-0.2115	-0.2114
**ρ_{rcp} (e^- Bohr^{-3})**		0.2044	
**$\nabla^2\rho_{rcp}$ (e^- Bohr^{-5})**		0.0703	

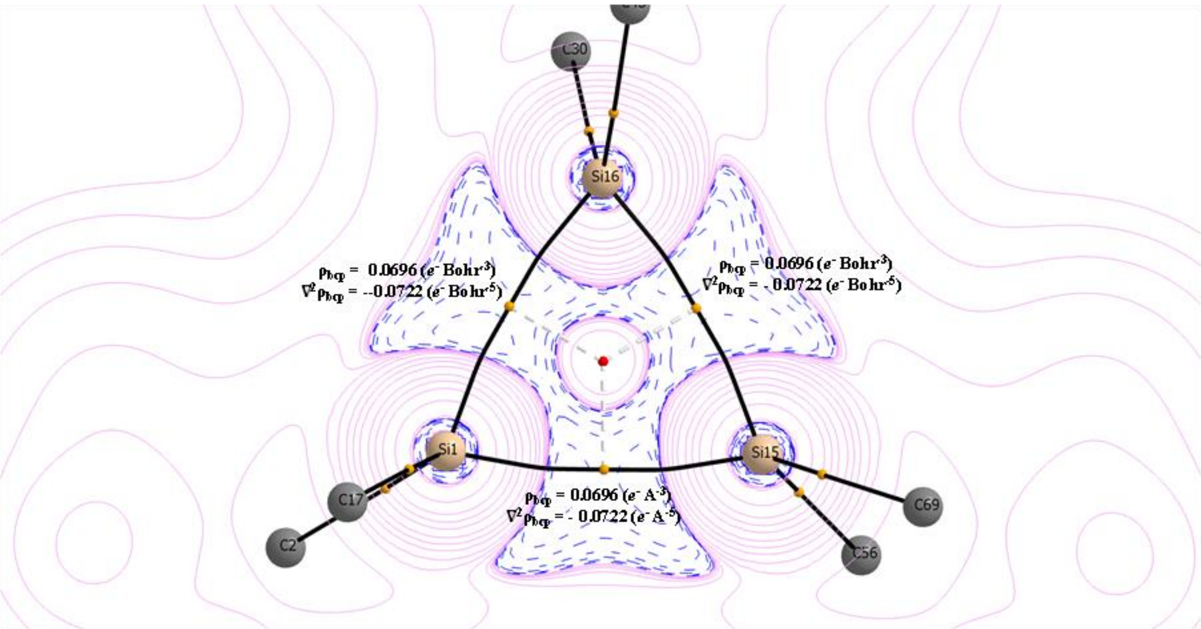

Figure S 51: QTAIM topology plot for the Laplacian of the electron density ($\nabla^2\rho$) of cyclotrisilane, highlighting areas of charge concentration (blue) and depletion (pink). The values of electron density (ρ) and $\nabla^2\rho$ are indicated at each bcp.

Table S 32: Data table for the QTAIM analysis of cyclotrisilane, indicating the electronic parameters found at the bcps as well as strain indicators (BPL-GBL_I) and rcp properties. Cremer and Kraka energy density parameters (V,G,K,H) are also presented.

	Si_1-Si_{15}	Si_1-Si_{16}	Si_{15}-Si_{16}
**ρ_{bcp} (e^- Bohr^{-3})**	0.0696	0.0696	0.0696
**$\nabla^2\rho_{bcp}$ (e^- Bohr^{-5})**	-0.0722	-0.0722	-0.0723
**BPL**	4.7829	4.7829	4.7828
**GBL_I**	4.7444	4.7444	4.7444
**BPL-GBL_I**	0.0385	0.0385	0.0385
**V**	-0.0349	-0.0349	-0.0349
**G**	0.0084	0.0084	0.0084
**K**	0.0265	0.0265	0.0265
**H**	-0.0265	-0.0265	-0.0265
**ρ_{rcp} (e^- Bohr^{-3})**		0.0476	
**$\nabla^2\rho_{rcp}$ (e^- Bohr^{-5})**		0.0378	

Canonical molecular orbitals - SMD(cyclohex)-M06-2X-GD3|def2-tzvp||M06-2X-GD3|def2-tzvp(Al,N)/def2-svp(C,H)

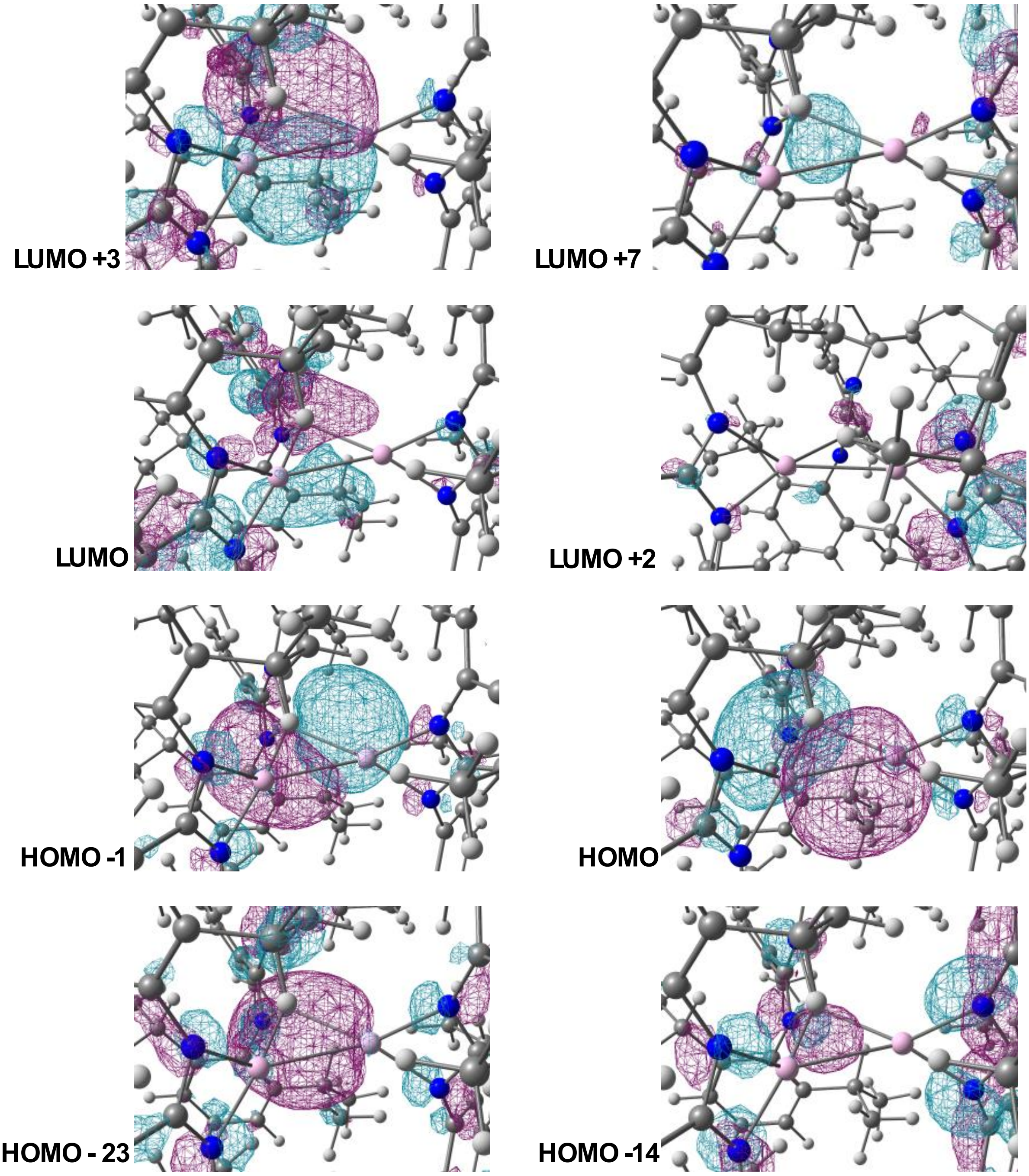

Figure S 52: DFT calculated canonical molecular orbitals of ***2****$^{p\text{-}tol}$. Specifically, orbitals involving areas of density localised around the Al_3 core, highlighting the presence of a low-lying radial sigma bonding combination (HOMO-23) as well as two effectively degenerate HOMO orbitals contributing to the overall sigma bonding skeleton of the cyclotrialumane.*

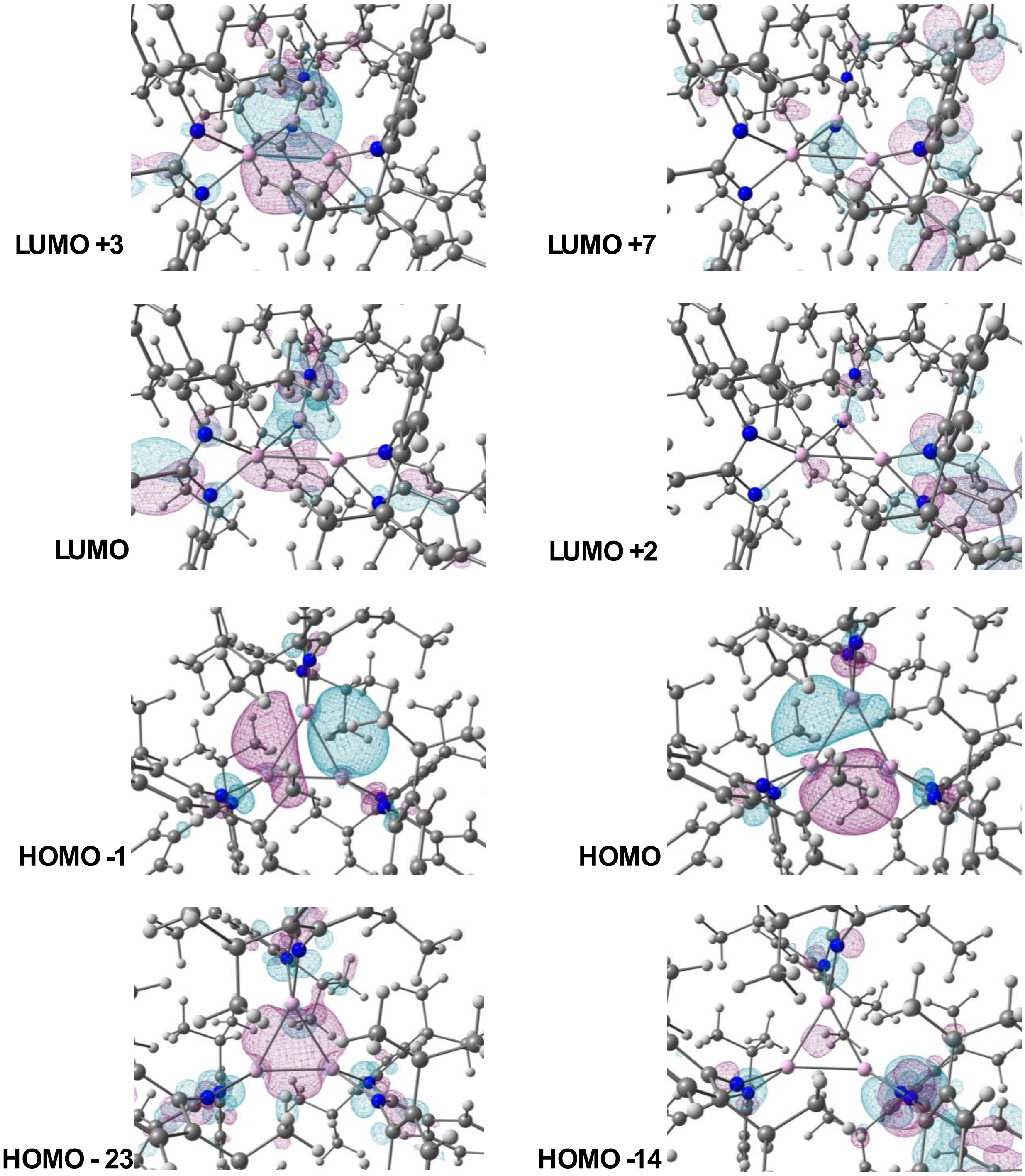

Figure S 53: DFT calculated canonical molecular orbitals of **2**$^{m\text{-}xyl}$. *Specifically, orbitals involving areas of density localised around the* Al_3 *core, highlighting the presence of a low-lying radial sigma bonding combination (HOMO-23) as well as two effectively degenerate HOMO orbitals (HOMO, HOMO -1) contributing to the overall sigma bonding skeleton of the cyclotrialumane.*

Natural bonding orbitals - SMD(cyclohex)-M06-2X-GD3|def2-tzvp||M06-2X-GD3|def2-tzvp(Al,N)/def2-svp(C,H)

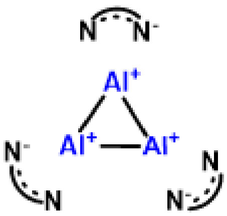

Figure S 54: Simplified schematic representation of the Lewis structure adopted by the NBO program

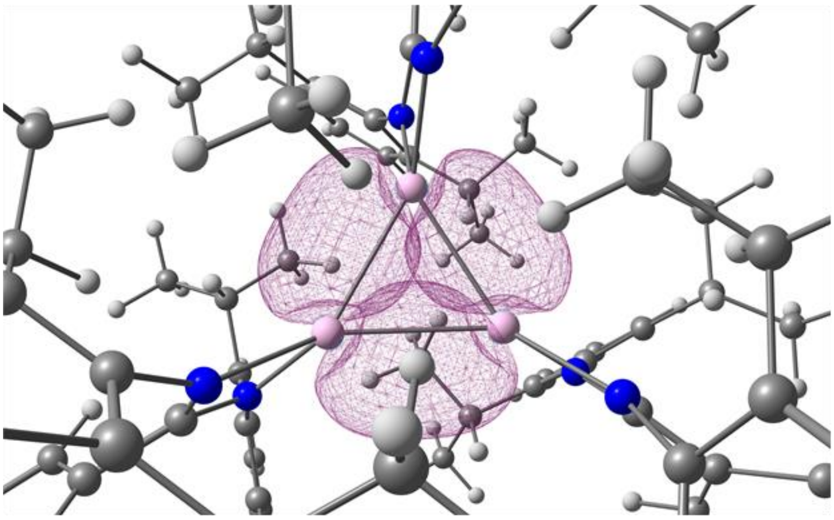

Figure S 55: Natural bonding orbitals of the Al_3 core of ***2***$^{p\text{-}tol}$*.*

Table S 33: The respective calculated occupancies of the natural bonding orbitals of the Al_3 core of ***2***$^{p\text{--}tol}$*.*

Lewis Quality			
97.60%			
**Bond**	**WBI**	**NBO Occupancy**	**Bond Orbital Composition**
**Al_1-Al_2**	0.9658	1.831 (50.69% Al_1)	Al1 [50.69%]: s(38.45%), p(60.87%), d(0.67%); Al2 [49.31%]: s(36.55%), p(62.77%), d(0.66%)
**Al_1-Al_3**	0.9697	1.836 (50.77% Al_3)	Al1 [49.23%]: s(37.34%), p(62.01%), d(0.64%); Al3 [50.77%]: s(37.85%), p(61.45%), d(0.69%)
**Al_2-Al_3**	0.9741	1.839 (50.92% Al_2)	Al2 [50.93%]: s(38.78%), p(60.63%), d(0.57%); Al3 [49.08%%]: s(37.27%), p(62.04%), d(0.68%)

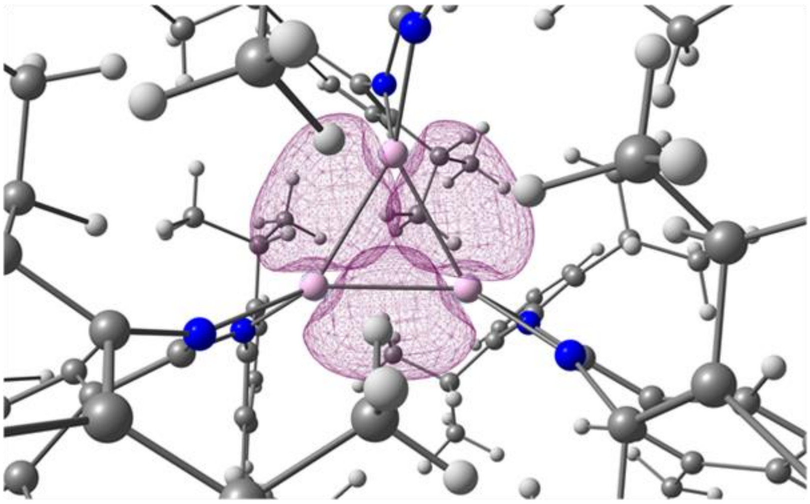

Figure S 56: Natural bonding orbitals of the Al_3 core of ***$2^{m\text{-}xyl}$***.

In line with the observed bond lengths, the NBO calculations suggest equal contributions to each Al–Al σ-bond from the component atoms (e.g. Al_1–Al_2 50.69%:49.31%) where each Al centre contributes a hybrid orbital with approximately 40% s- and 60% p-character (Table S34). A comparable description of the Al–Al σ-bonds emerges when Intrinsic Bond Orbitals (IBOs) are used as the localisation method (Fig. S61).

Table S 34: The respective calculated occupancies of the natural bonding orbitals of the Al_3 core of ***$2^{m\text{-}xyl}$***.

**Lewis Quality**			
97.65%			
**Bond**	**WBI**	**NBO Occupancy**	**Bond Orbital Composition**
**Al_1-Al_2**	0.9663	1.832 (50.64% Al_1)	Al1 [50.64%]: s(38.31%), p(61.01%), d(0.67%); Al2 [49.36%]: s(36.62%), p(62.71%), d(0.65%)
**Al_1-Al_3**	0.9705	1.837 (50.69% Al_3)	Al1 [49.31%]: s(37.38%), p(61.97%), d(0.65%); Al3 [50.69%]: s(37.82%), p(61.48%), d(0.69%)
**Al_2-Al_3**	0.9744	1.839 (50.87% Al_2)	Al2 [50.87%]: s(38.67%), p(60.75%), d(0.57%); Al3 [49.13%%]: s(37.25%), p(62.07%), d(0.67%)

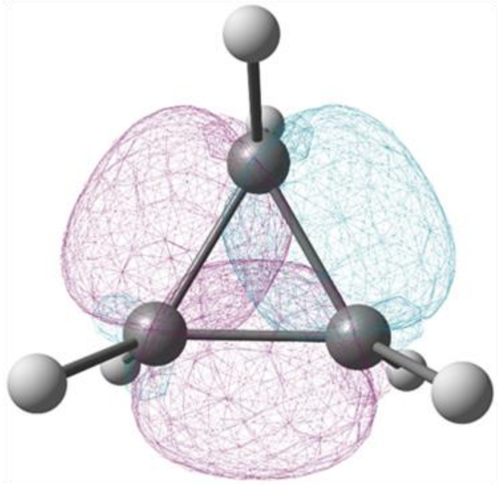

Figure S 57: Natural bonding orbitals of the C_3 core of cyclopropane.

Table S 35: The respective calculated occupancies of the natural bonding orbitals of the C_3 core of cyclopropane.

Lewis Quality			
99.34%			
**Bond**	**WBI**	**NBO Occupancy**	**Bond Orbital Composition**
**C_1-C_2**	1.0000	1.969 (50% C_1-C_2)	C1 [50%]: s(21.96%), p(77.79%), d(0.24%); C2 [50%]: s(21.95%), p(77.80%), d(0.24%)
**C_1-C_3**	0.9999	1.969 (50% C_1-C_3)	C1 [50%]: s(21.92%), p(77.82%), d(0.24%); C3 [50%]: s(21.95%), p(77.80%), d(0.24%)
**C_2-C_3**	0.9999	1.969 (50% C_2-C_3)	C2 [50%]: s(21.93%), p(77.81%), d(0.24%); C3 [50%]: s(21.92%), p(77.82%), d(0.24%)

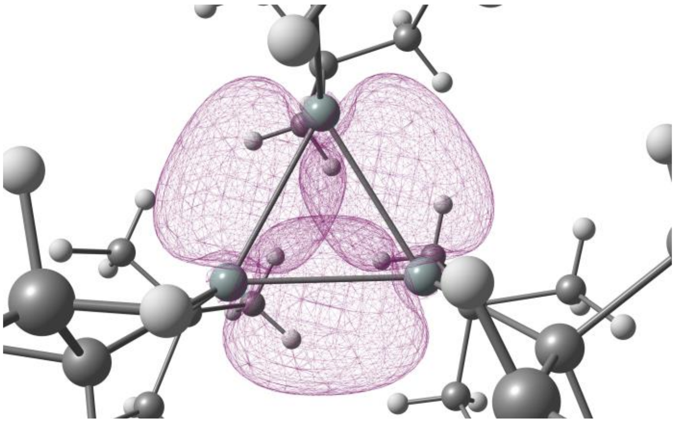

Figure S 58: Natural bonding orbitals of cyclotrisilane.

Table S 36: The respective calculated occupancies of the natural bonding orbitals of cyclotrisilane.

		**Lewis Quality**	
		99.28%	
**Bond**	**WBI**	**NBO Occupancy**	**Bond Orbital Composition**
**Si_1-Si_2**	0.9367	1.90739 (50% Si_1-Si_2)	Si1 [50%]: s(20.29%), p(79.10%), d(0.60%); Si2 [50%]: s(20.28%), p(79.10%), d(0.60%)
**Si_1-Si_3**	0.9367	1.90739 (50% Si_1-Si_3)	Si1 [50%]: s(20.29%), p(79.10%), d(0.60%); Si3 [50%]: s(20.28%), p(79.10%), d(0.60%)
**Si_2-Si_3**	0.9367	1.90738 (50% Si_2-Si_3)	Si2 [50%]: s(20.28%), p(79.10%), d(0.60%); Si3 [50%]: s(20.68%), p(79.10%), d(0.60%)

Second order perturbation theory

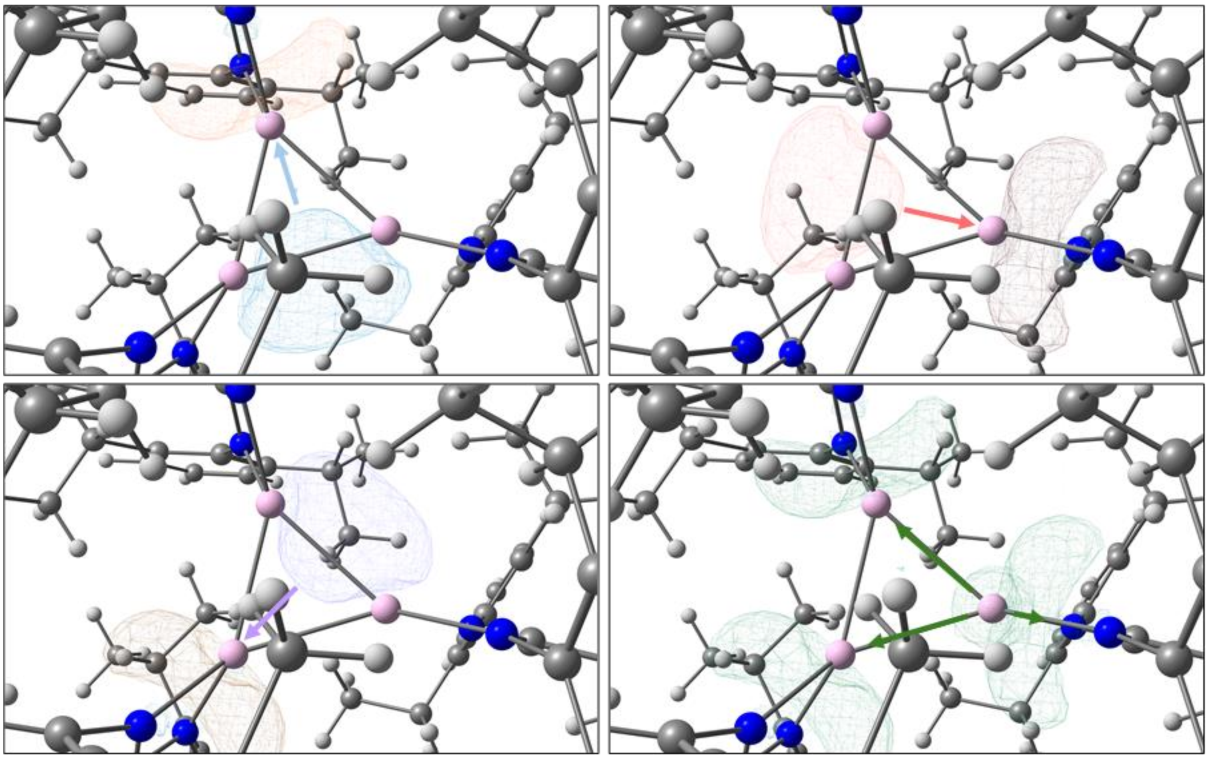

*Figure S 59: Donor-acceptor interactions of **2**$^{p\text{-}tol}$ calculated via second order perturbation theory analysis, for interactions of E(2)> 5.00 kcal/mol. For simplicity, only one of three interactions involving donations directly from an Al centre (shown in green) is highlighted. Two more symmetric interactions from the remaining Al_2 and Al_3 are observed.*

The dominant interactions within the cyclotrialumane result from hyperconjugation between each Al–Al σ-bond and the vacant acceptor p-orbitals on the opposite Al centres (Fig. S59), which second order perturbation theory quantifies as $E^{(2)}$ ~ 37 kcal/mol in each case (Table S37).

*Table S 37: Tabulated donor-acceptor interactions of **2**$^{p\text{-}tol}$ calculated via second order perturbation theory analysis for interactions of E(2)> 5.00 kcal/mol. For simplicity, only one of three interactions involving donations directly from an Al centre (shown in green) is highlighted. Two more symmetric interactions from the remaining Al_2 and Al_3 are observed.*

Donor NBO	Acceptor NBO	E(2) kcal/mol
BD Al_1-Al_2	LP* Al_3	36.75
BD Al_1-Al_3	LP* Al_2	36.74
BD Al_2-Al_3	LP* Al_1	36.23
CR(2) Al_1	LP* Al_1	10.36
CR(2) Al_1	LP* Al_2	5.90
CR(2) Al_1	LP* Al_3	6.25

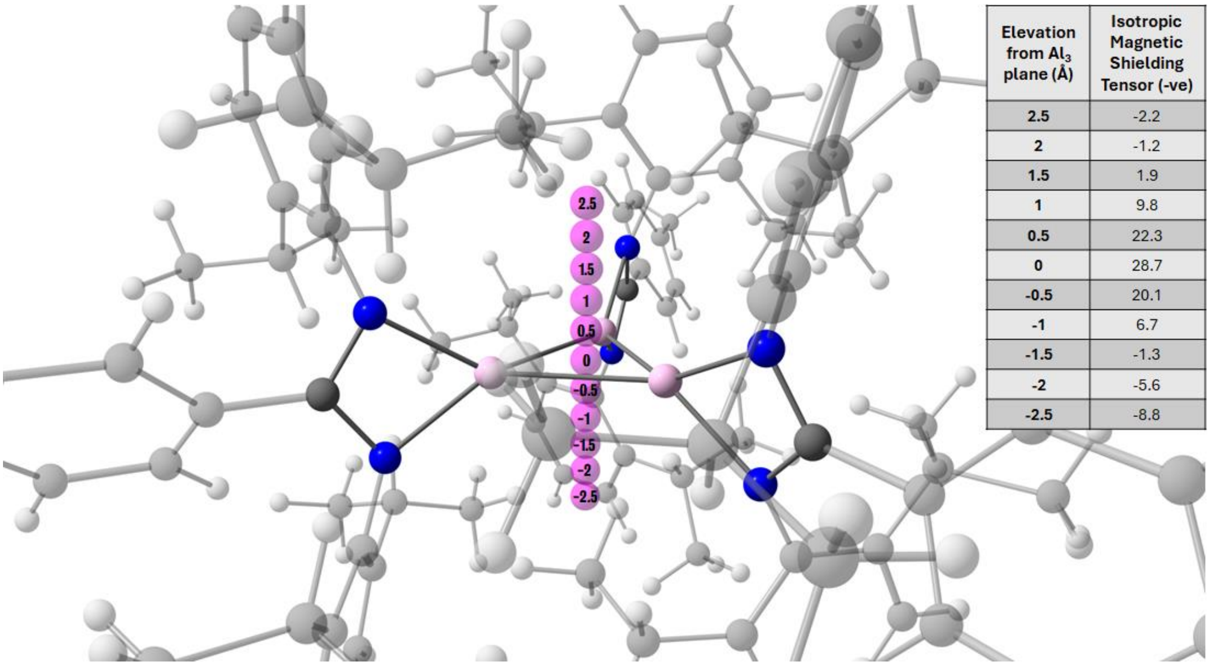

Elevation from Al_3 plane (Å)	Isotropic Magnetic Shielding Tensor (-ve)
2.5	-2.2
2	-1.2
1.5	1.9
1	9.8
0.5	22.3
0	28.7
-0.5	20.1
-1	6.7
-1.5	-1.3
-2	-5.6
-2.5	-8.8

*Figure S 60: Negative isotropic magnetic shielding tensor values obtained from NICS analysis of **2**$^{p\text{-}tol}$.*

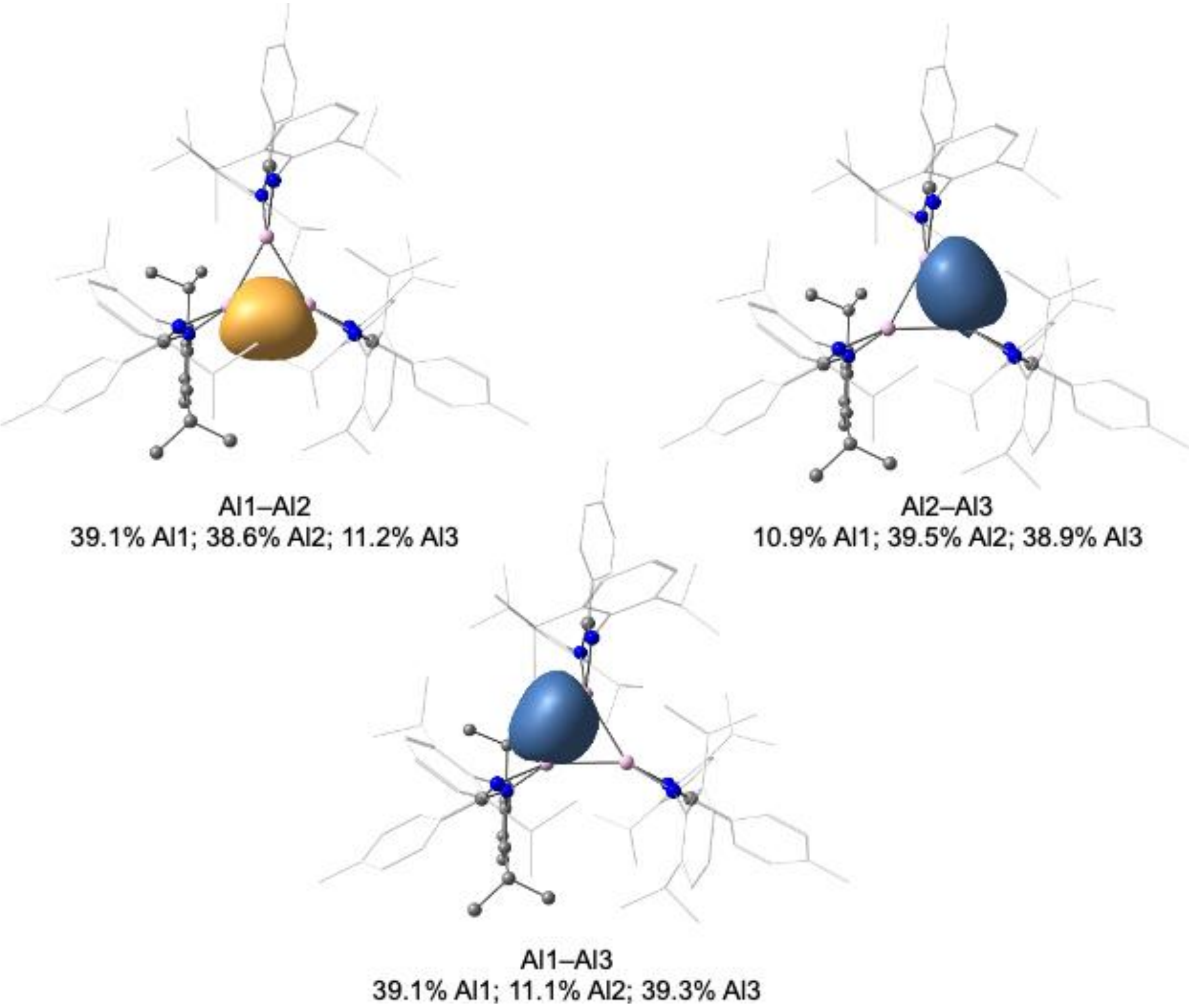

*Figure S 61: Isosurfaces for Intrinsic Bond Orbitals (pau) of **2**$^{p\text{-}tol}$ (isovalue: 0.05 au) calculated at the M06-2X-d3/def2-TZVP level of theory. Contributions from individual centres are provided.*

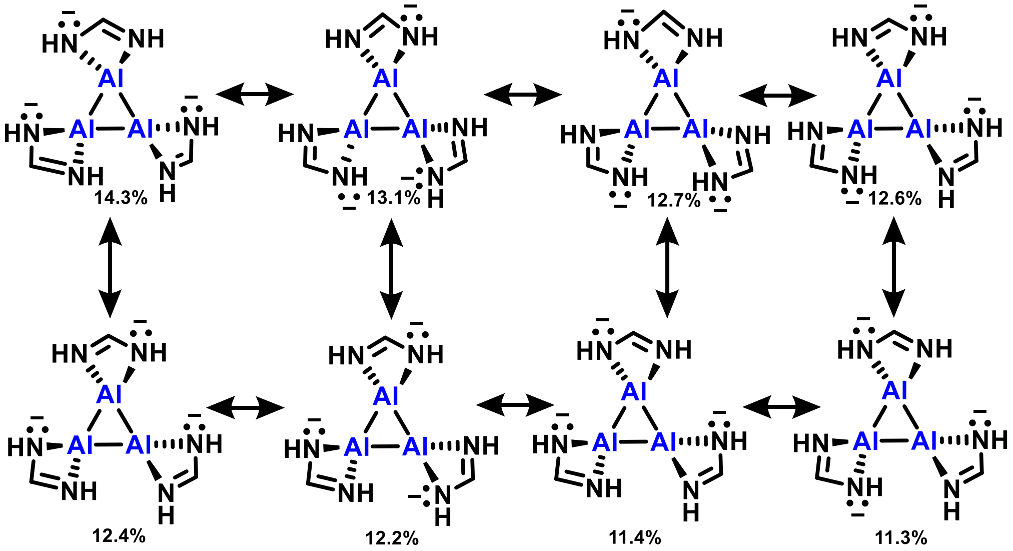

Figure S 62: Resonance forms derived from Natural resonance Theory (NRT) calculations. Al_3 core remains intact throughout.

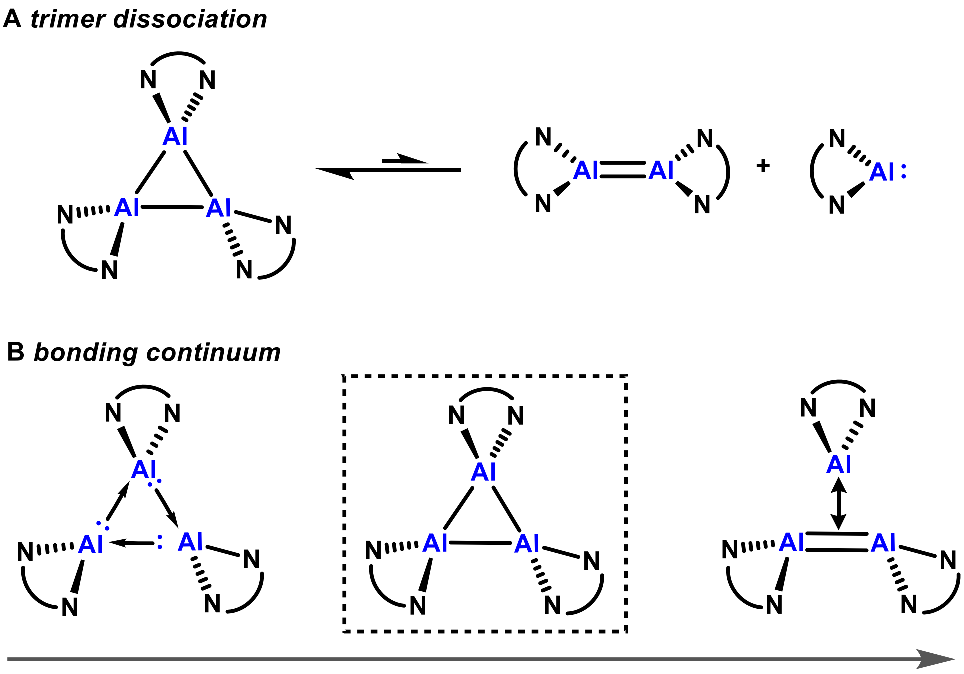

Figure S 63: ***A*** *Proposed trimer dissociation; experimental and computational support the equilibrium lying significantly towards the trimer* ***B*** *Al_3 trimer bonding continuum. All computational evidence points to the 2-centre 2-electron bonding motif (square box) providing the best description of bonding at the Al_3 core.*

Mechanistic Analysis - SMD(benzene) -M06-2X-GD3|def2-svp

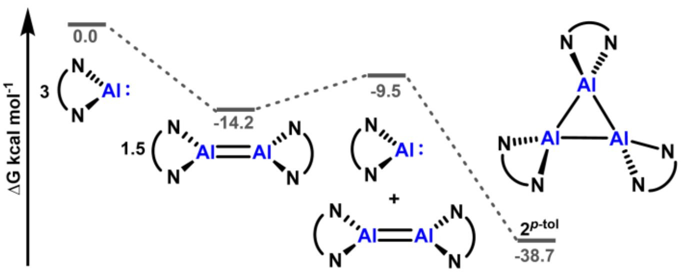

Figure S 64*: Energy surface diagram showing the relative energies of monomeric, dimeric and trimeric para-tolyl amidine-Al(I) fragments.*

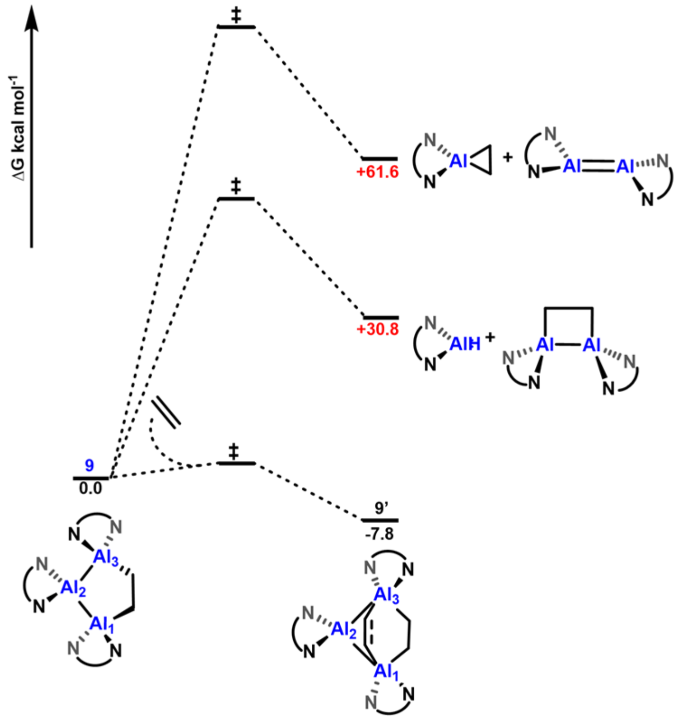

Figure S 65*: Energy surface diagram for the formation of truncated intermediate* ***9'*** *from* ***9****, compared to other possible intramolecular pathways affording alternative precursors to* ***11*** *and* ***12****.*

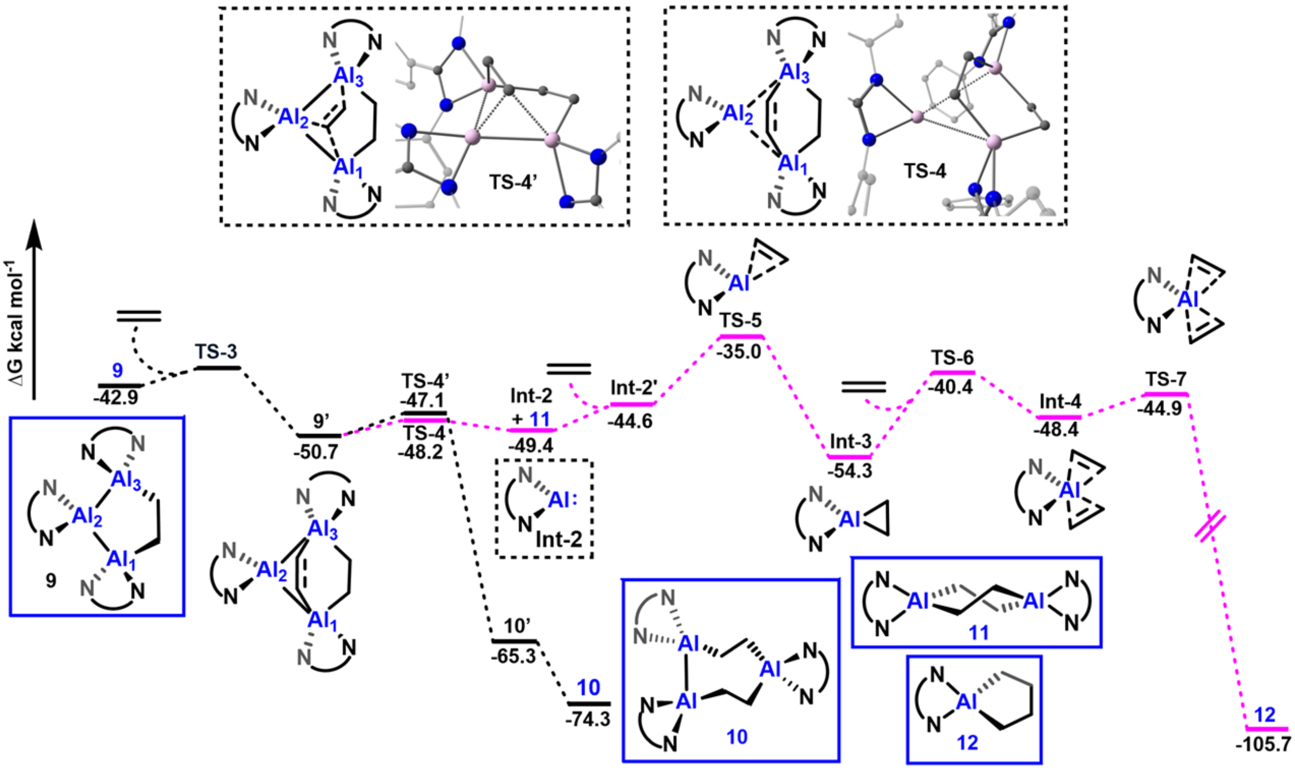

Figure S 66: Full reaction mechanism for the formation of truncated products **10**, **11** and **12** from **9** and the resulting intermediate **9'**.

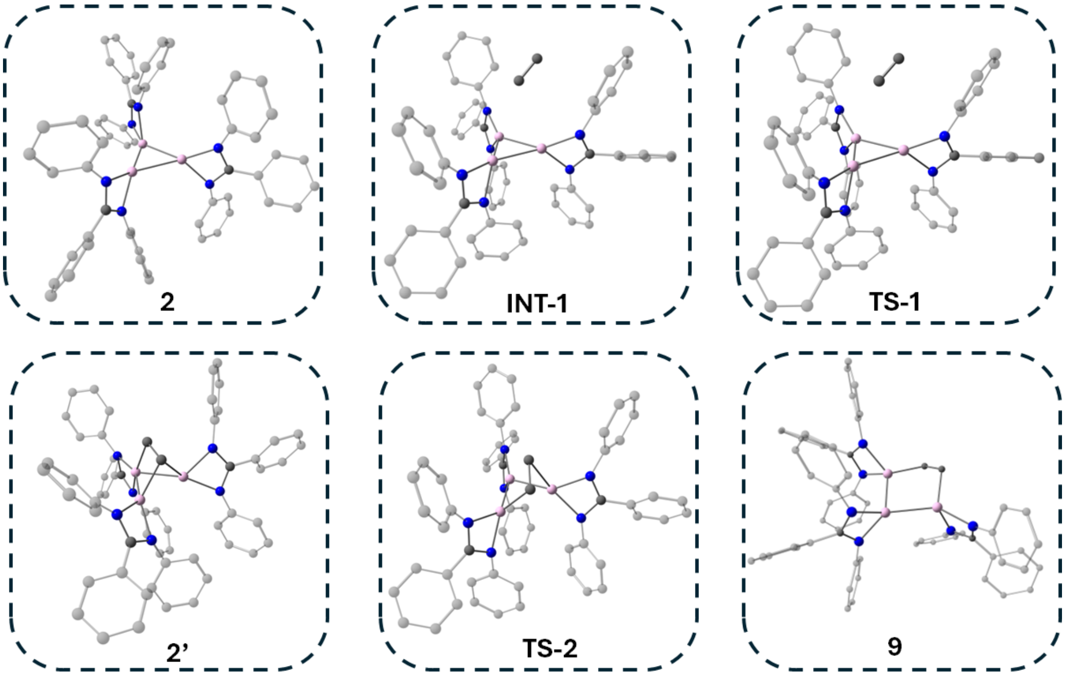

*Figure S 67: Calculated geometries of all transition states and intermediates involved in the formation of truncated **9** from complex truncated **2**.*

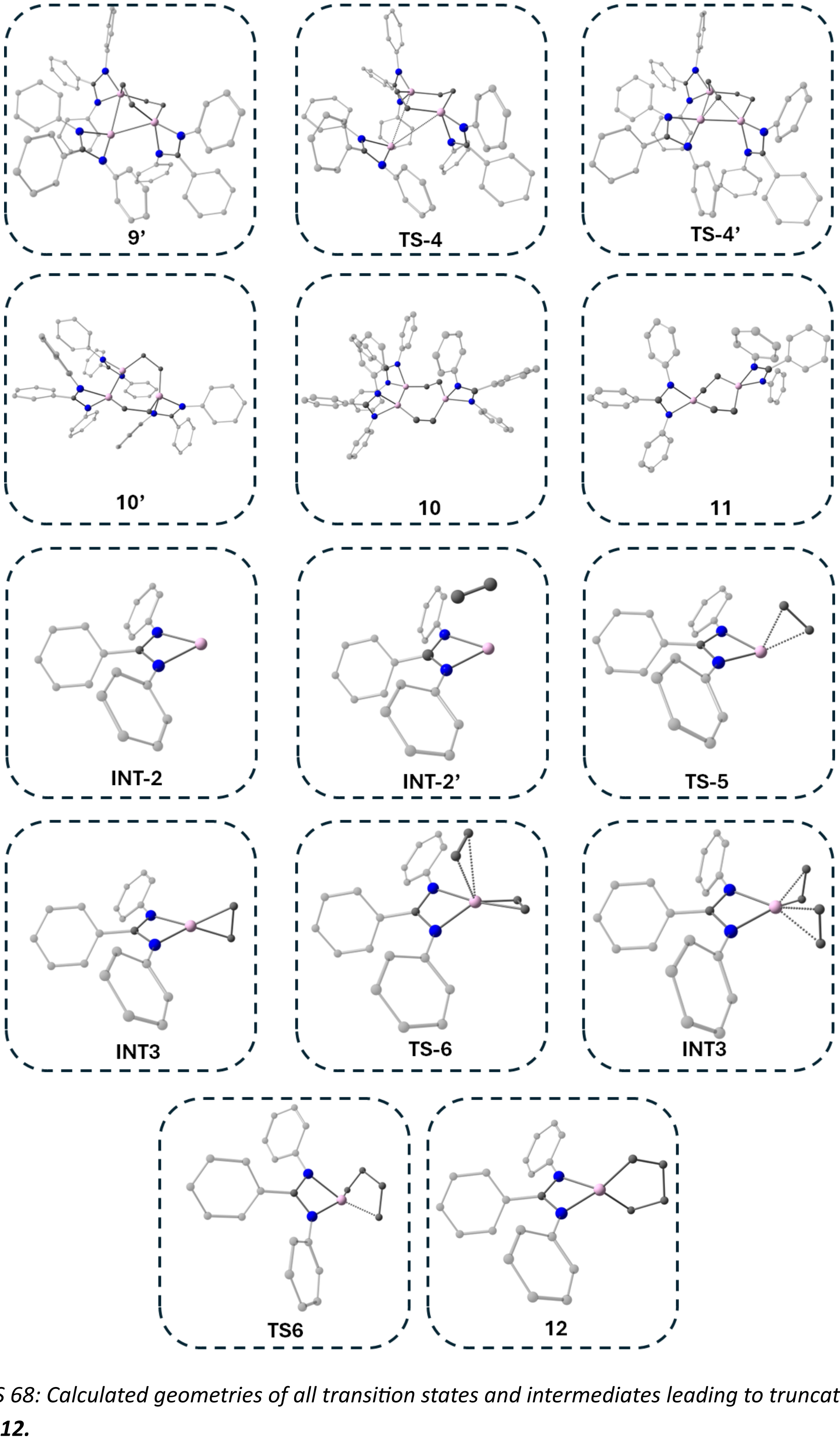

*Figure S 68: Calculated geometries of all transition states and intermediates leading to truncated **10**, **11** and **12**.*

6. Multinuclear NMR data

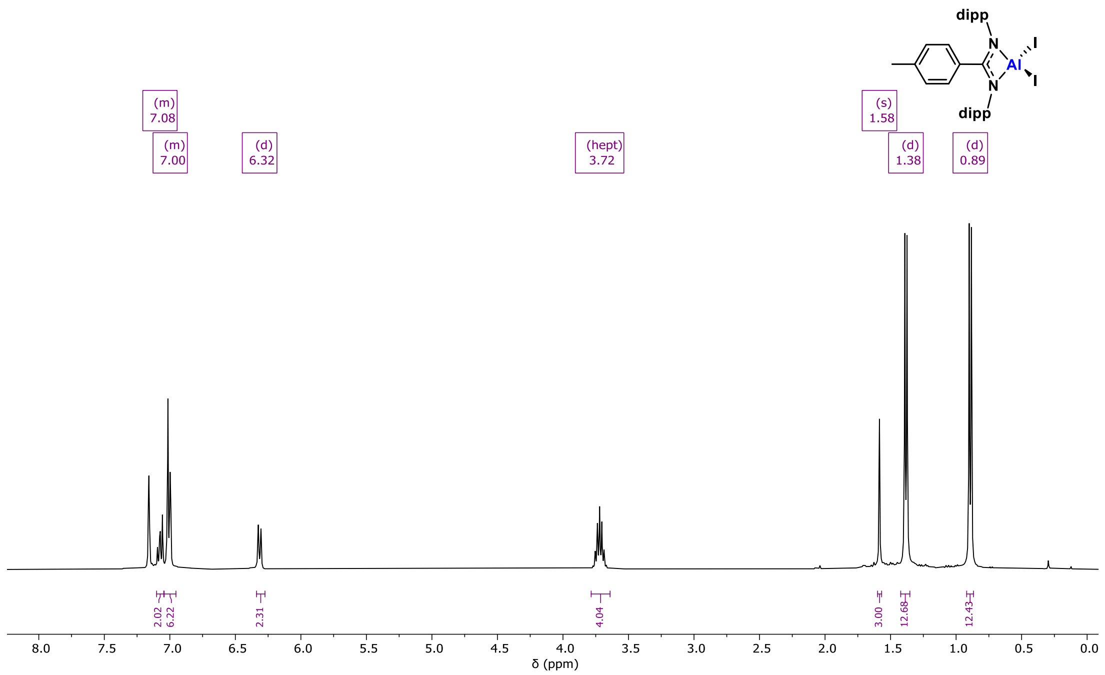

Figure S 69: ^{1}H NMR (400 MHz, 298 K) spectrum of ***1***$^{p\text{-}tol}$ *in benzene-d$_6$*

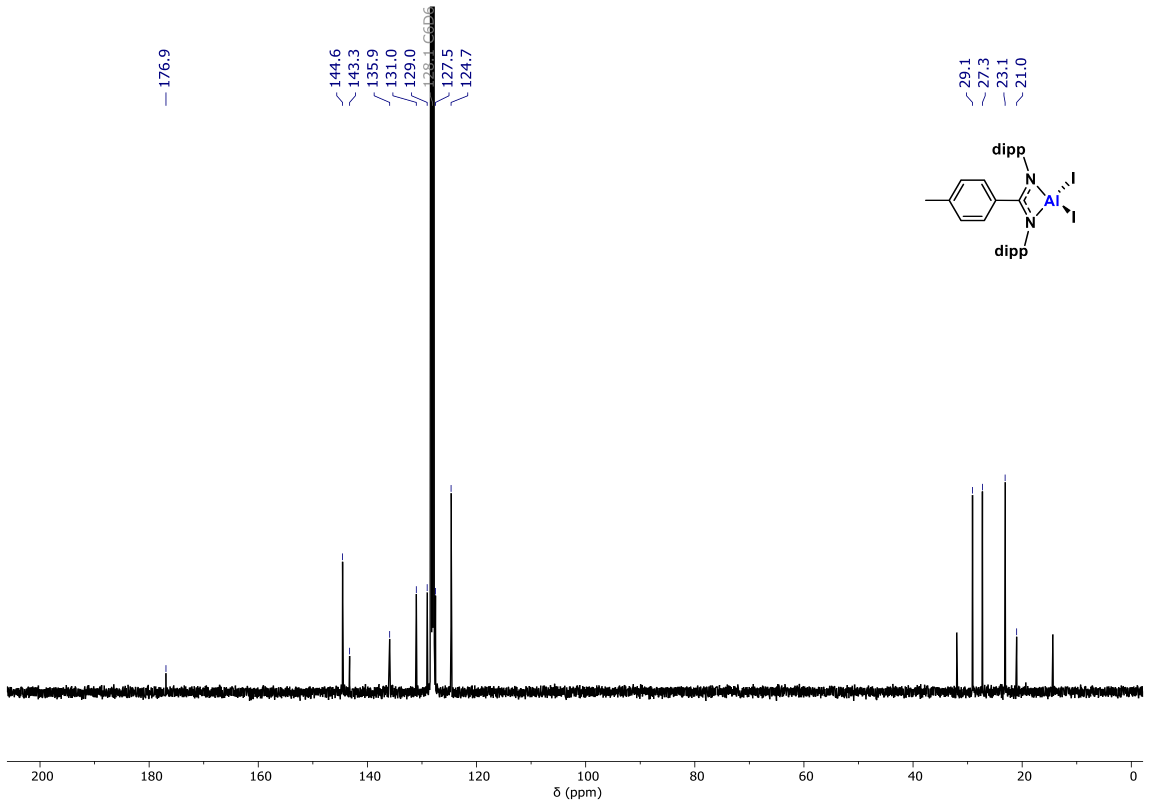

Figure S 70: ^{13}C{^{1}H} NMR (101 MHz, 298 K) spectrum of ***1***$^{p\text{-}tol}$ *in benzene-d$_6$*

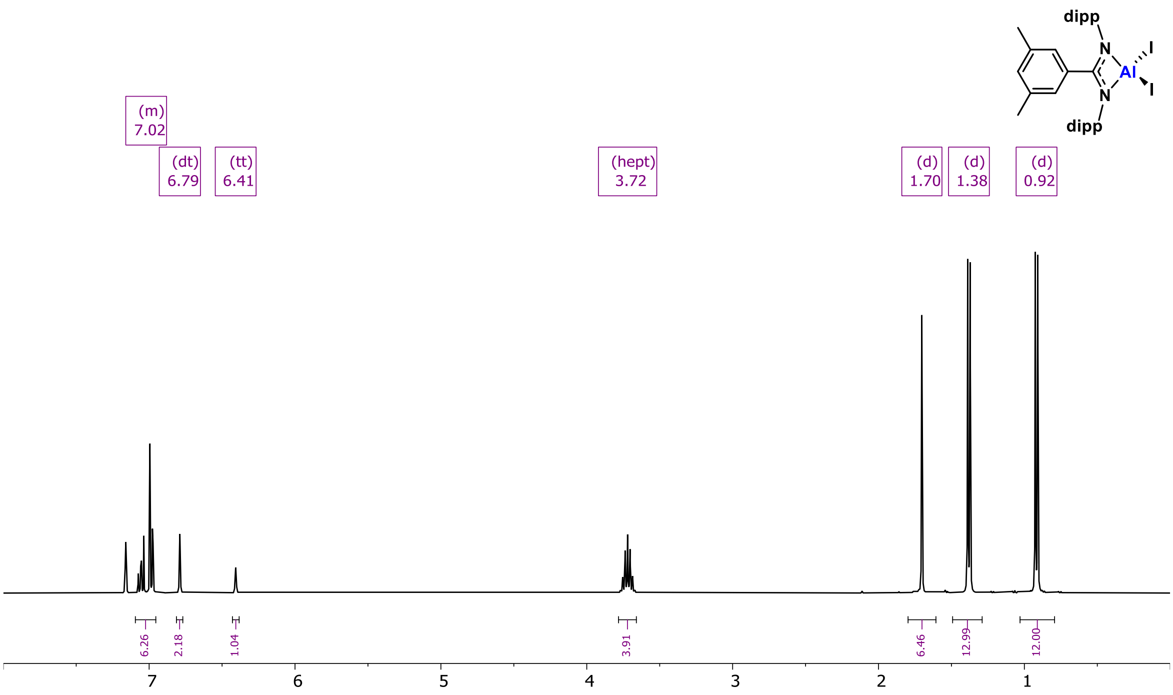

Figure S 71: ^{1}H NMR (400 MHz, 298 K) spectrum of ***1****$^{m\text{-}xyl}$ in benzene-d$_6$*

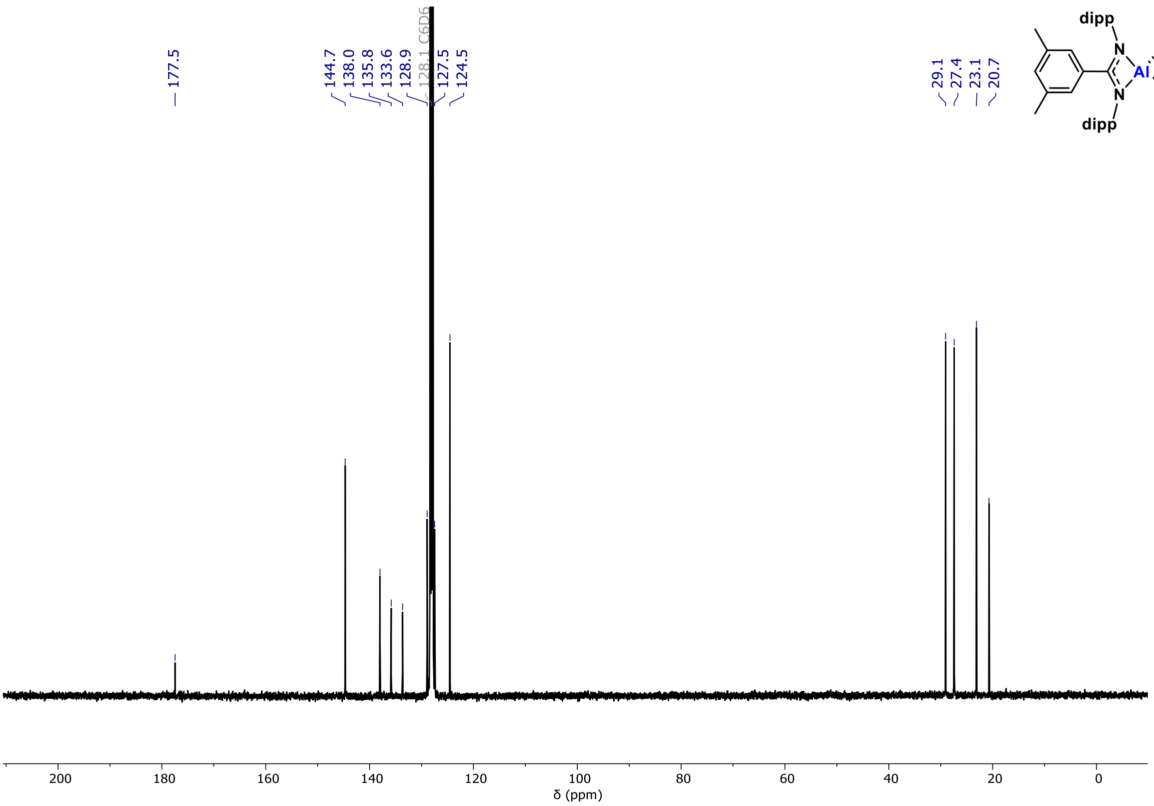

Figure S 72: ^{13}C{^{1}H} NMR (101 MHz, 298 K) spectrum of ***1****$^{m\text{-}xyl}$ in benzene-d$_6$*

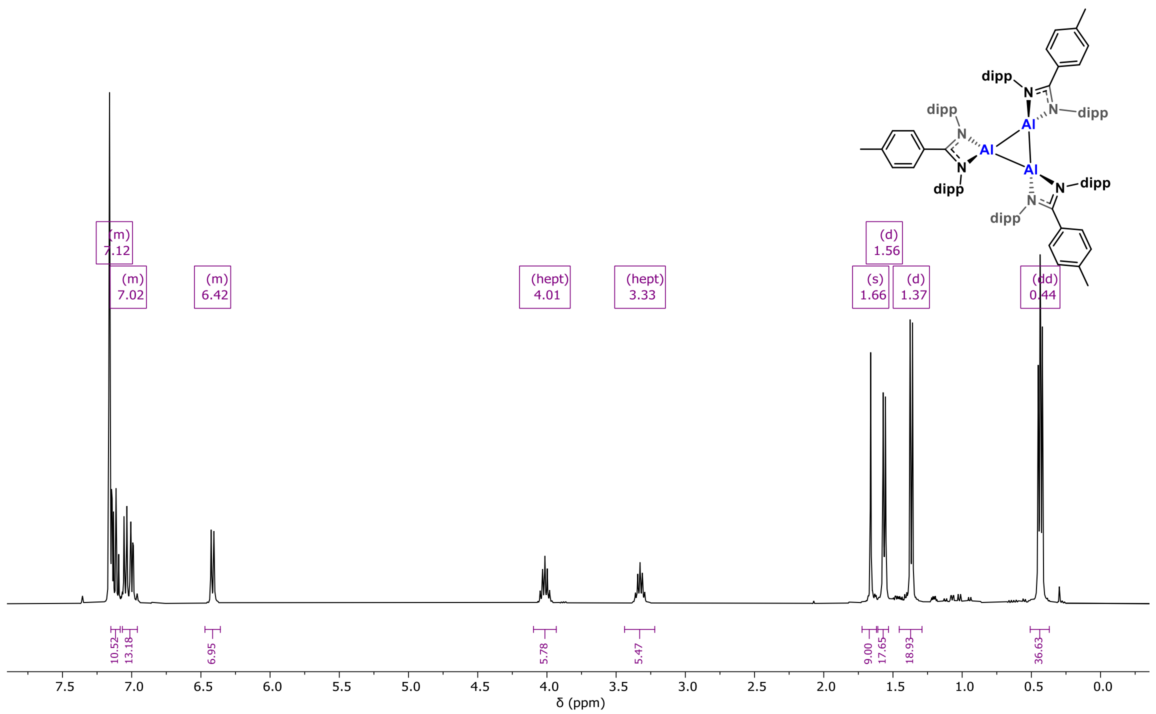

*Figure S 73: ^{1}H NMR (400 MHz, 298 K) spectrum of **2$^{p\text{-}tol}$** in benzene-d_6*

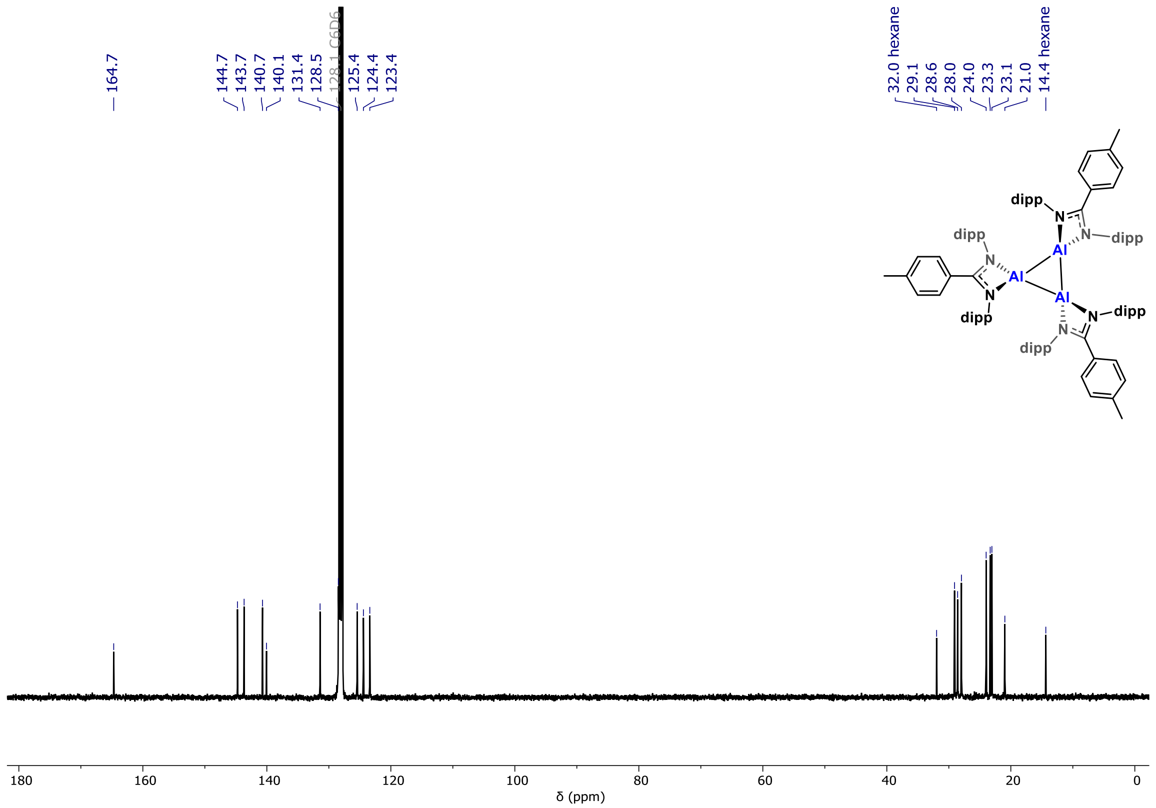

*Figure S 74: ^{13}C{^{1}H} NMR (101 MHz, 298 K) spectrum of **2$^{p\text{-}tol}$** in benzene-d_6*

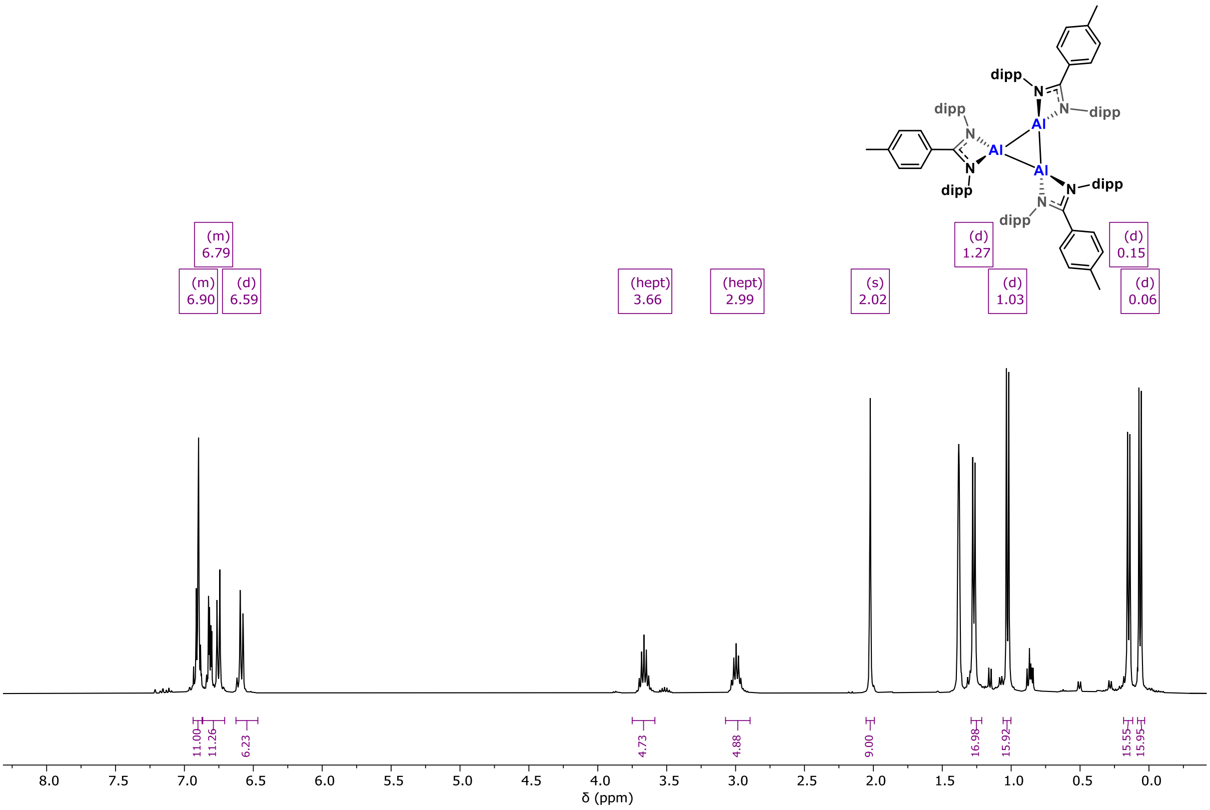

*Figure S 75: ^{1}H NMR (400 MHz, 298 K) spectrum of **2**$^{p\text{-}tol}$ in cyclohexane-d_{12}*

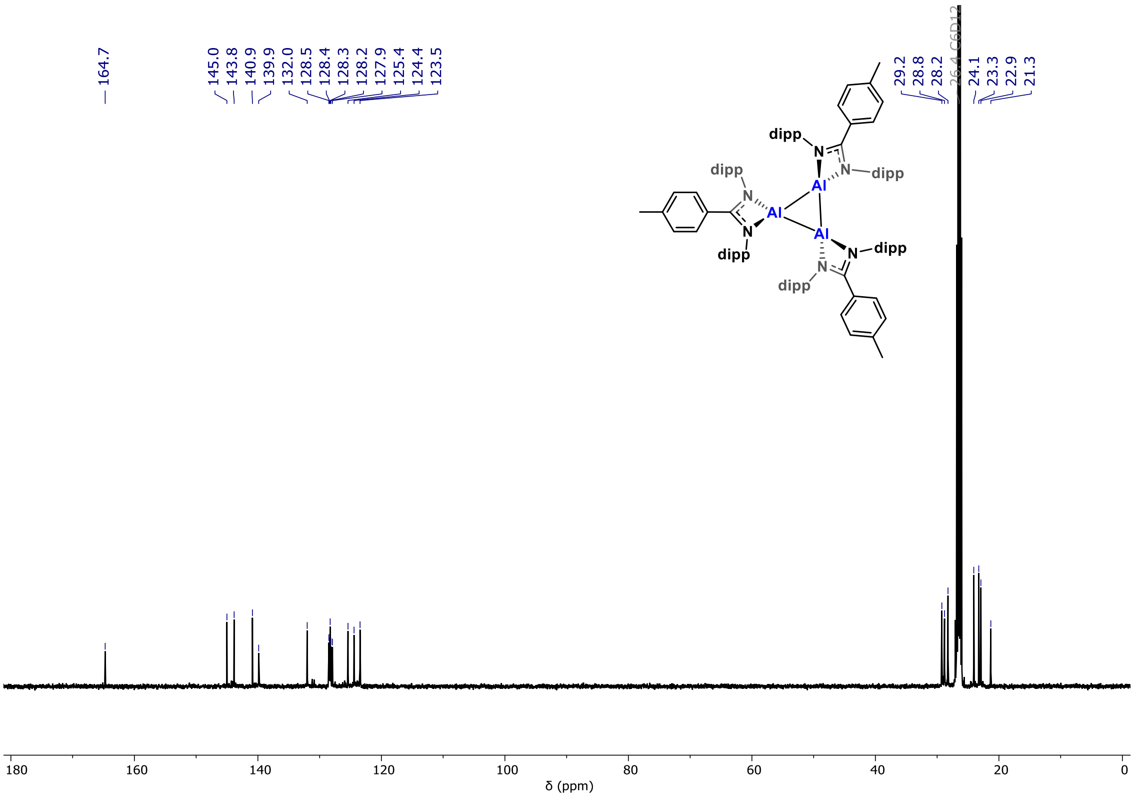

*Figure S 76: $^{13}C\{^{1}H\}$ NMR (101 MHz, 298 K) spectrum of **2**$^{p\text{-}tol}$ in cyclohexane-d_{12}*

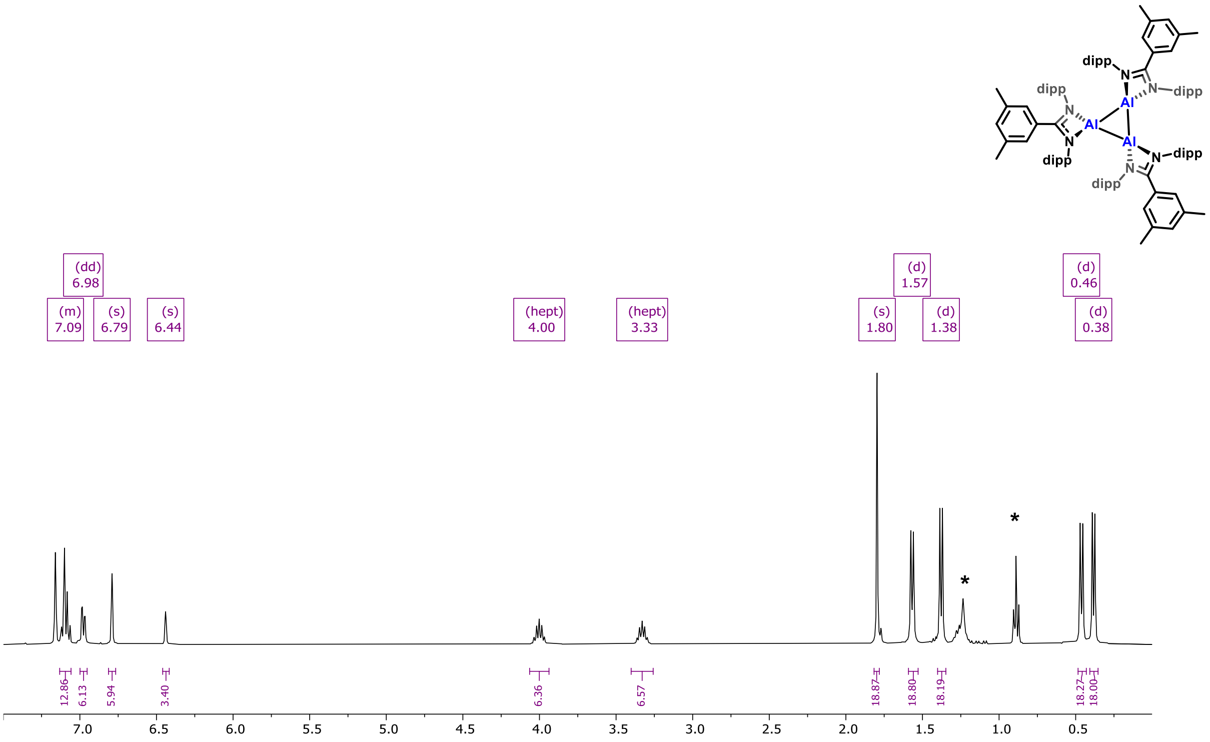

*Figure S 77: ^{1}H NMR (400 MHz, 298 K) spectrum of **2$^{m\text{-}xyl}$** in benzene-d$_6$ (*residual hexane)*

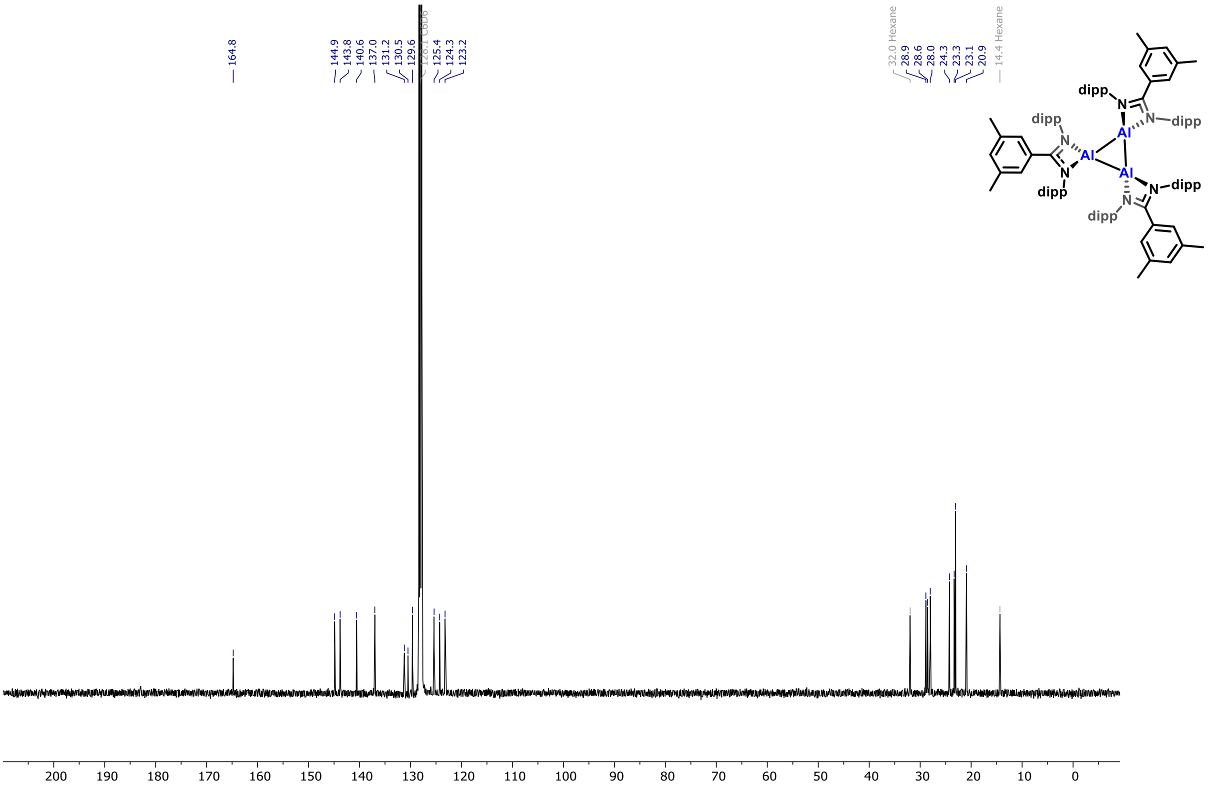

*Figure S 78: ^{13}C{^{1}H} NMR (101 MHz, 298 K) spectrum of **2$^{m\text{-}xyl}$** in benzene-d$_6$*

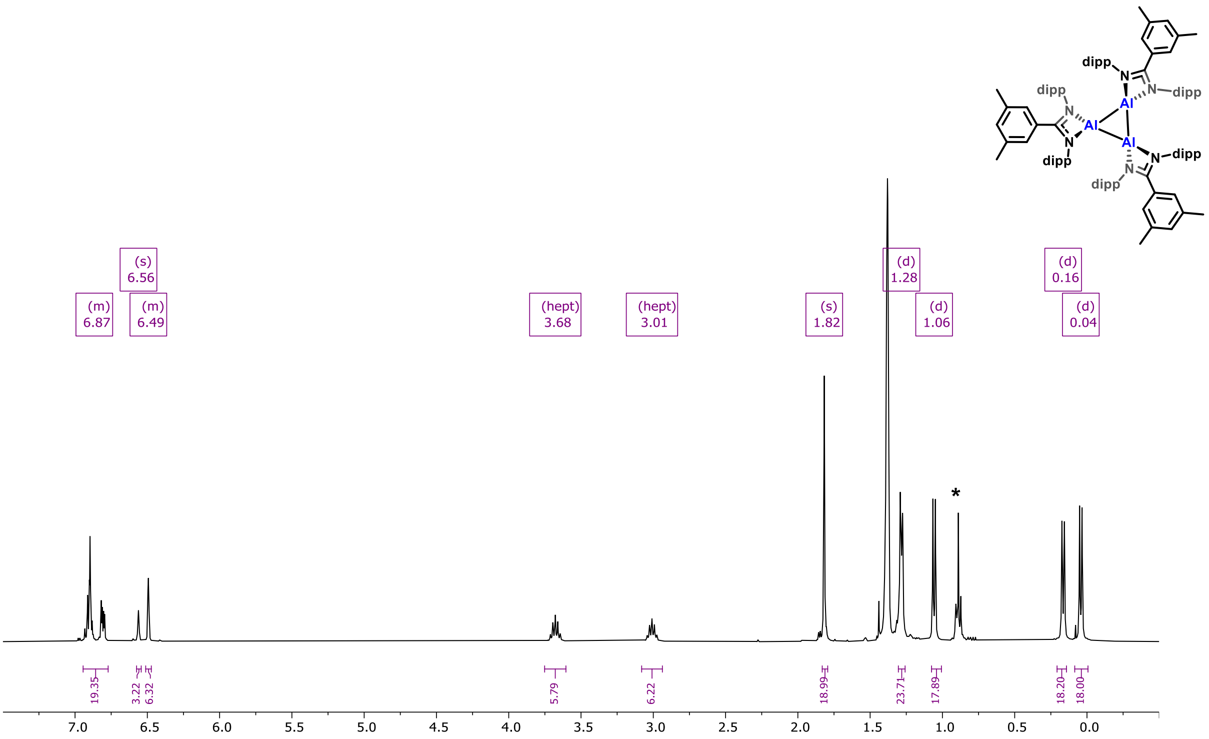

Figure S 79: ^{1}H NMR (400 MHz, 298 K) spectrum of ***2**$^{m\text{-}xyl}$ *in cyclohexane-d$_{12}$ (*residual hexane)*

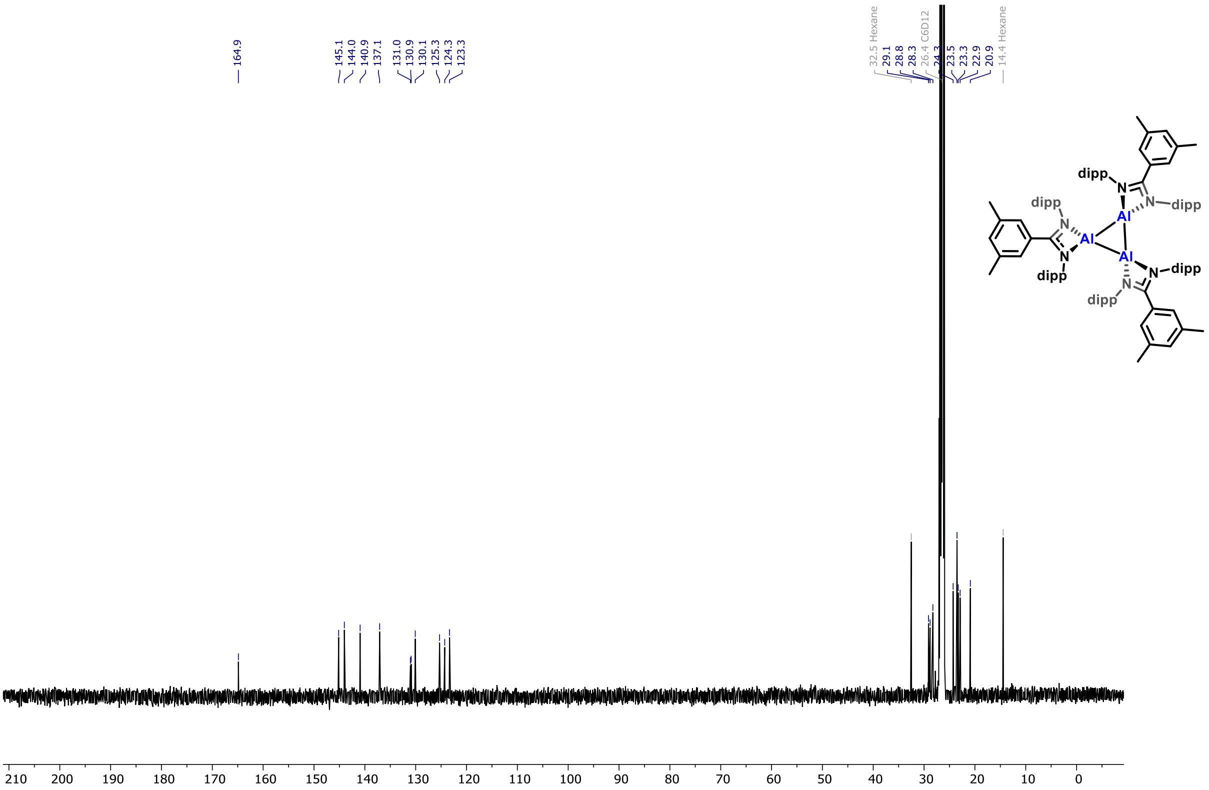

Figure S 80: ^{13}C{^{1}H} NMR (101 MHz, 298 K) spectrum of ***2**$^{m\text{-}xyl}$ *in cyclohexane-d$_{12}$*

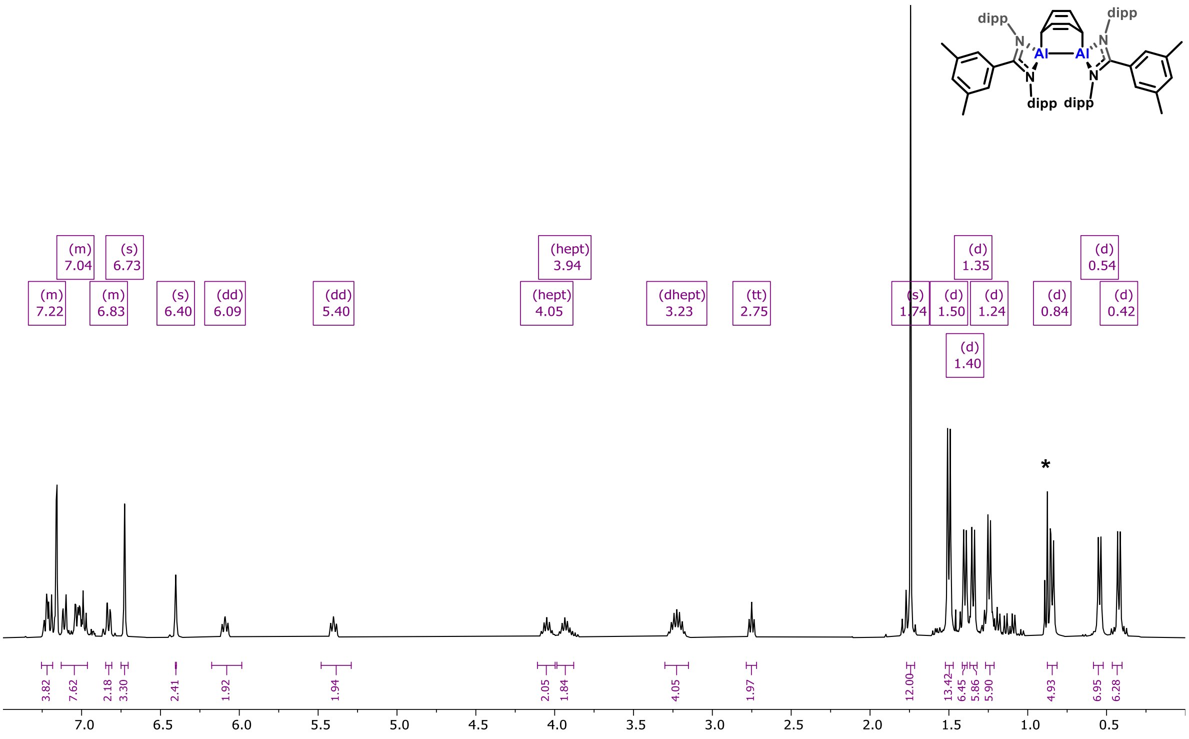

Figure S 81: ^{1}H NMR (400 MHz, 298 K) spectrum of ***3****$^{m\text{-}xyl}$ in benzene-d_6 (* residual hexane)*

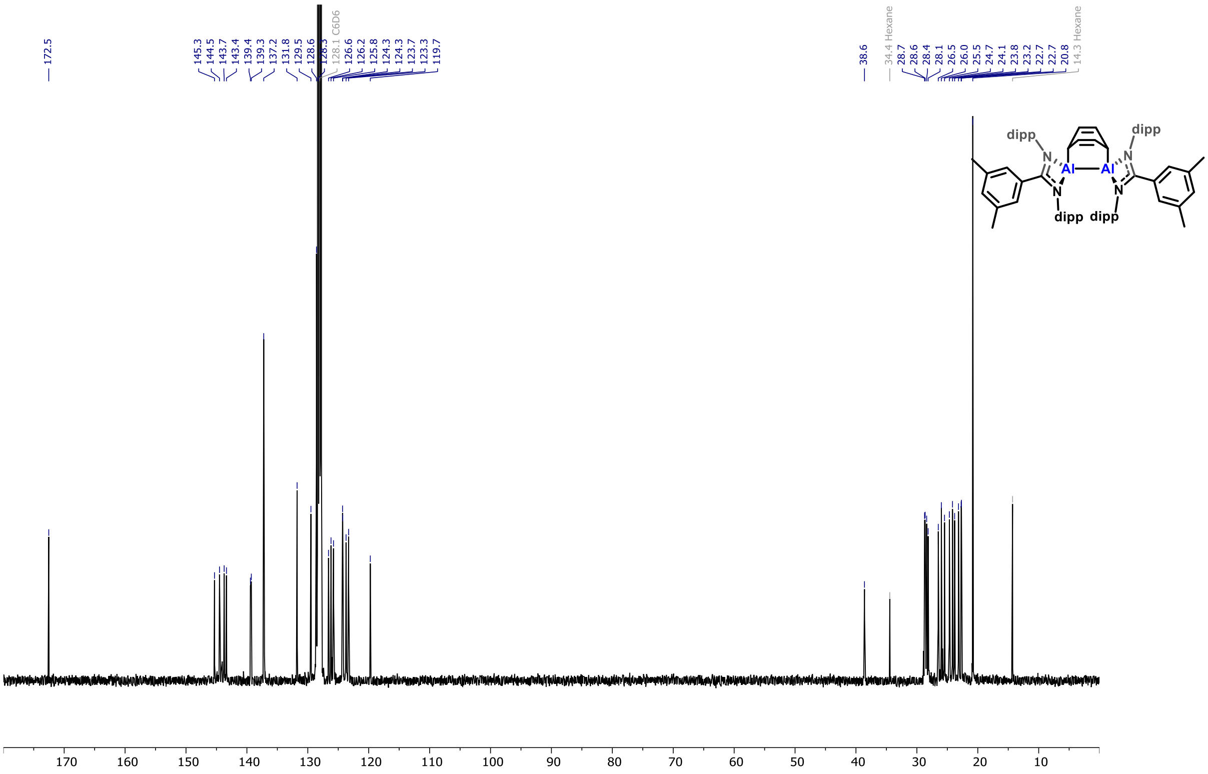

Figure S 82: $^{13}C\{^{1}H\}$ NMR (101 MHz, 298 K) spectrum of ***3****$^{m\text{-}xyl}$ in benzene-d_6*

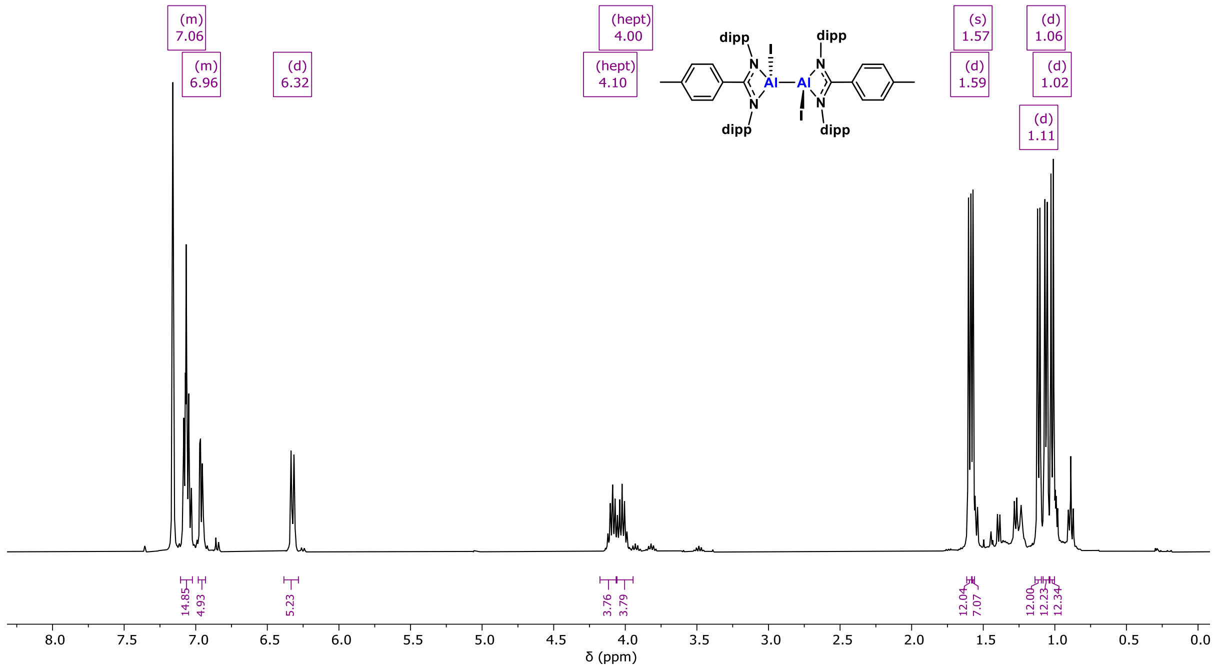

Figure S 83: ^{1}H NMR (400 MHz, 298 K) spectrum of ***5**^p-tol^* *in benzene-d_6*

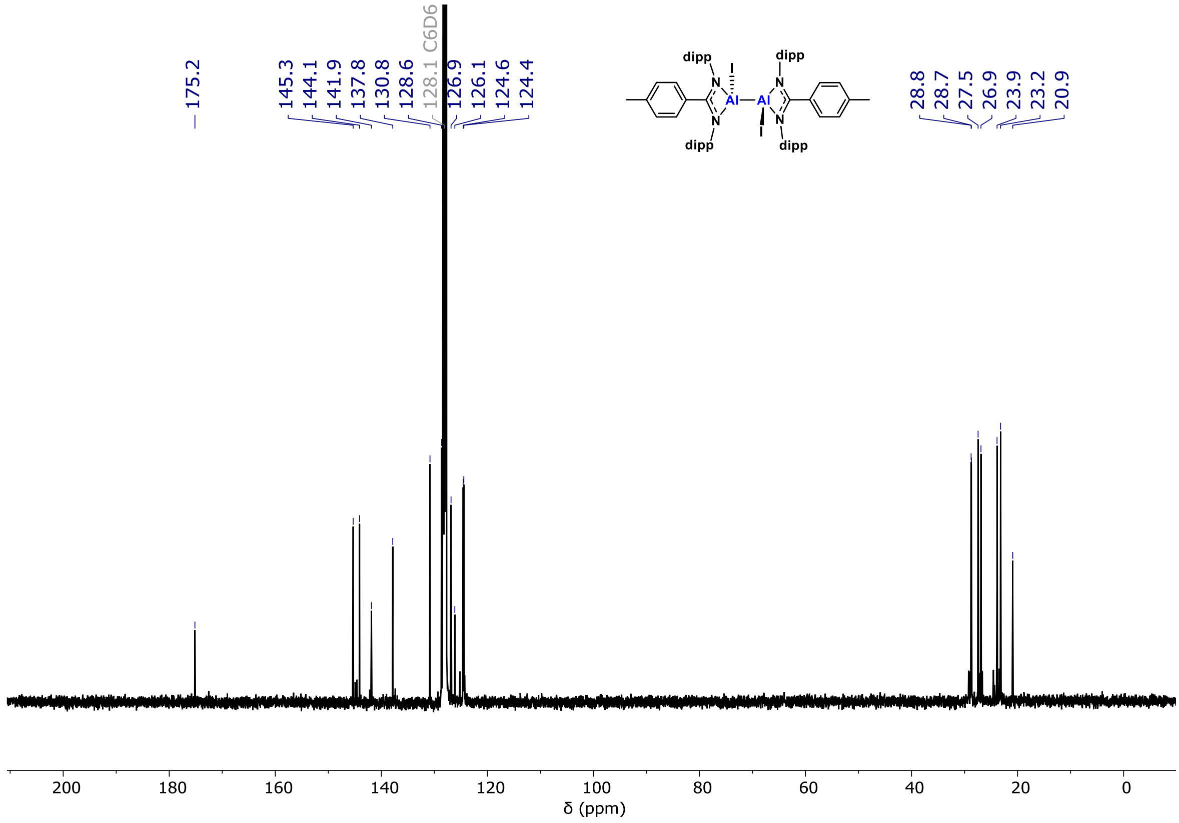

Figure S 84: ^{13}C{^{1}H} NMR (101 MHz, 298 K) spectrum of ***5**^p-tol^* *in benzene-d_6*

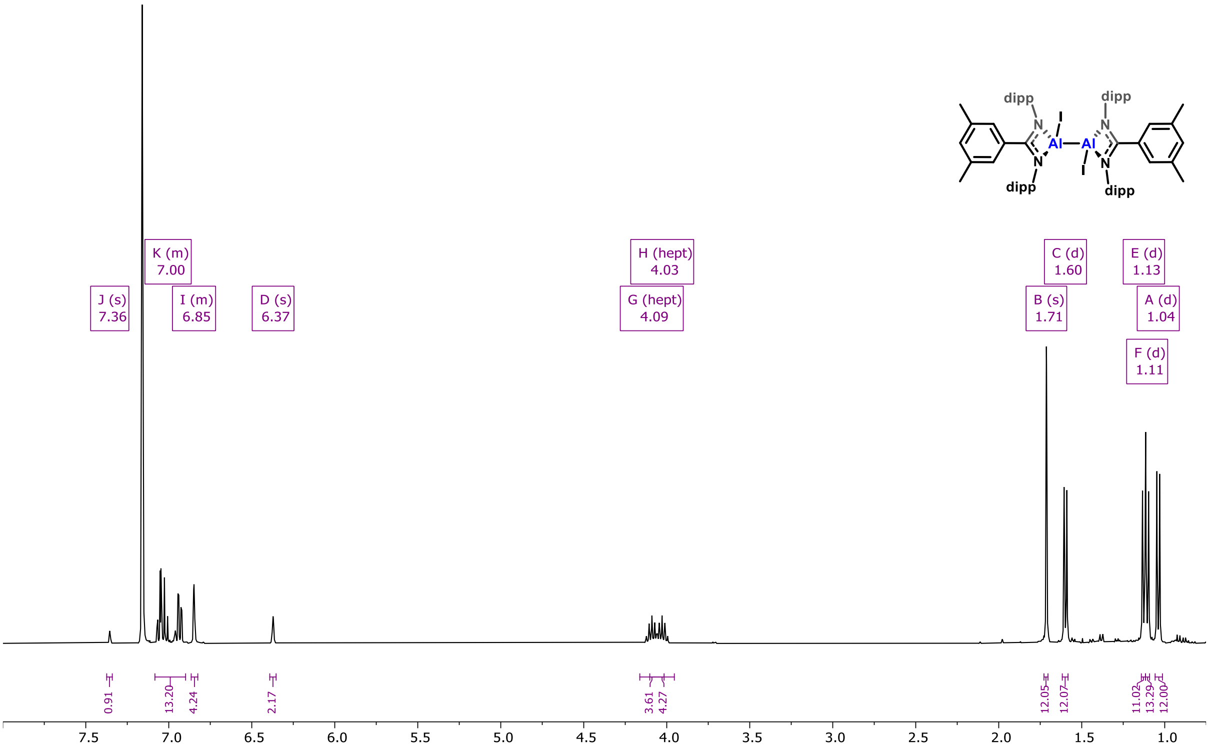

Figure S 85: ^{1}H NMR (400 MHz, 298 K) spectrum of ***5****$^{m\text{-}xyl}$ in benzene-d$_6$*

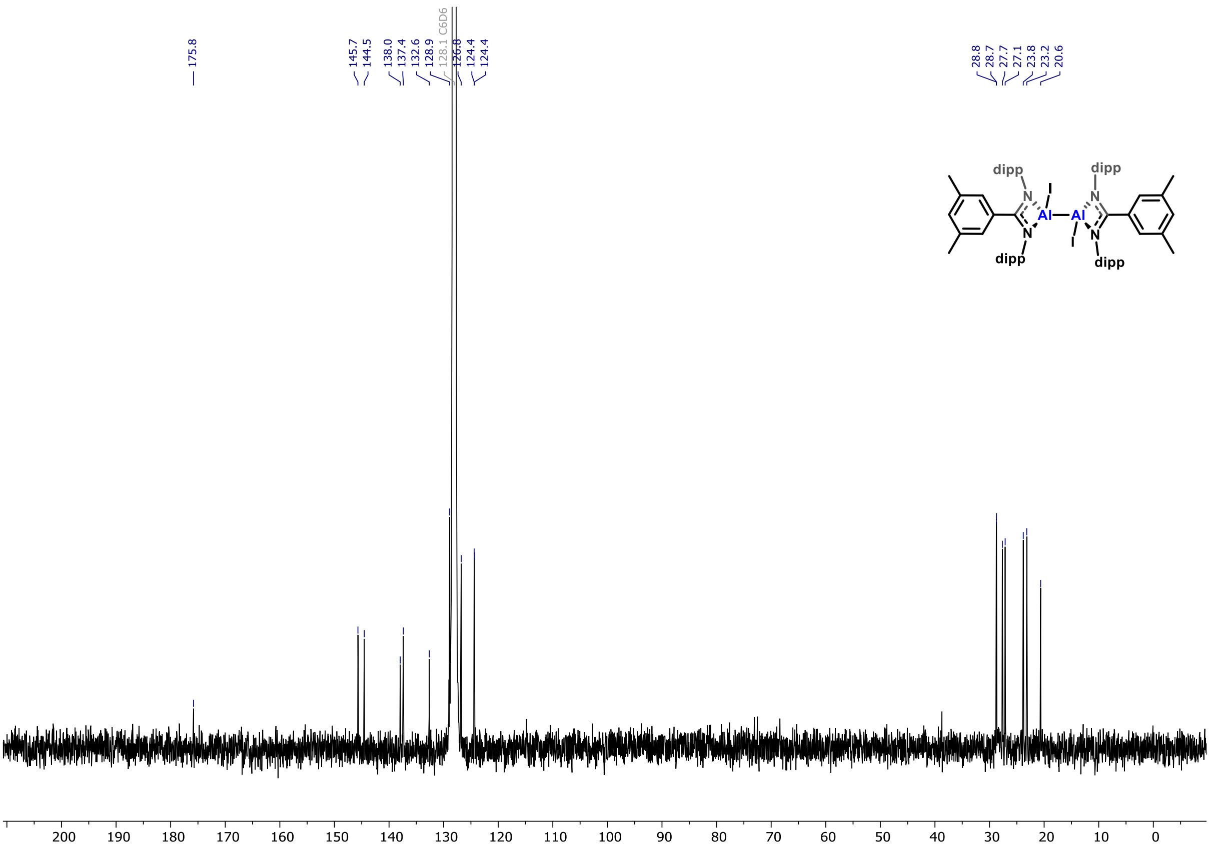

Figure S 86: ^{13}C{^{1}H} NMR (101 MHz, 298 K) spectrum of ***5****$^{m\text{-}xyl}$ in benzene-d$_6$*

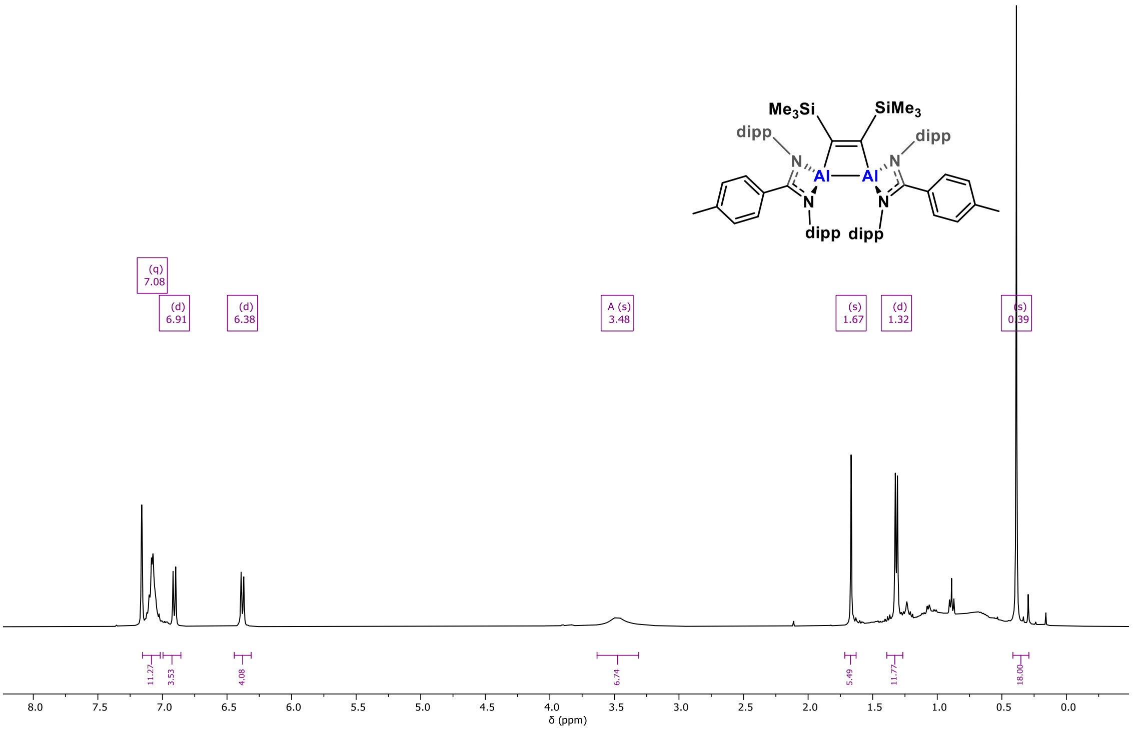

*Figure S 87: ^{1}H NMR (400 MHz, 298 K) spectrum of **6$^{p\text{-}tol}$** in benzene-d$_6$*

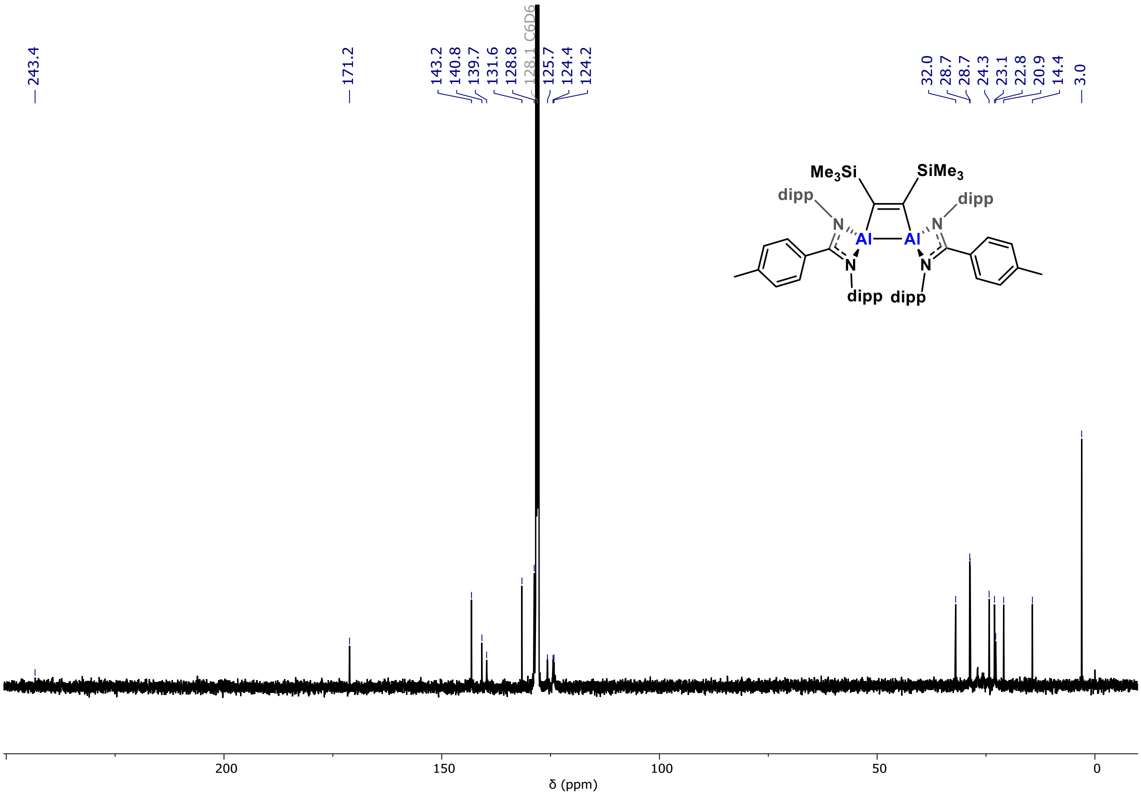

*Figure S 88: ^{13}C{^{1}H} NMR (101 MHz, 298 K) spectrum of **6$^{p\text{-}tol}$** in benzene-d$_6$*

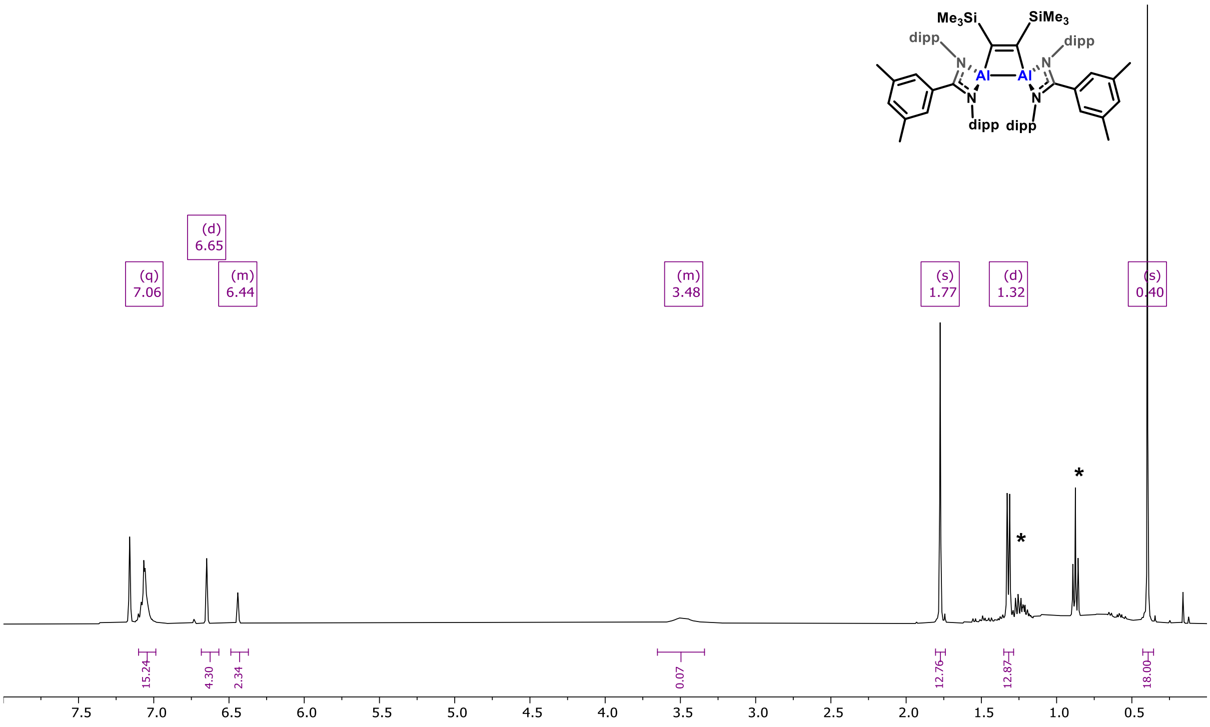

Figure S 89: ^{1}H NMR (400 MHz, 298 K) spectrum of $\mathbf{6}^{m\text{-}xyl}$ in benzene-d_6 (residual pentane)*

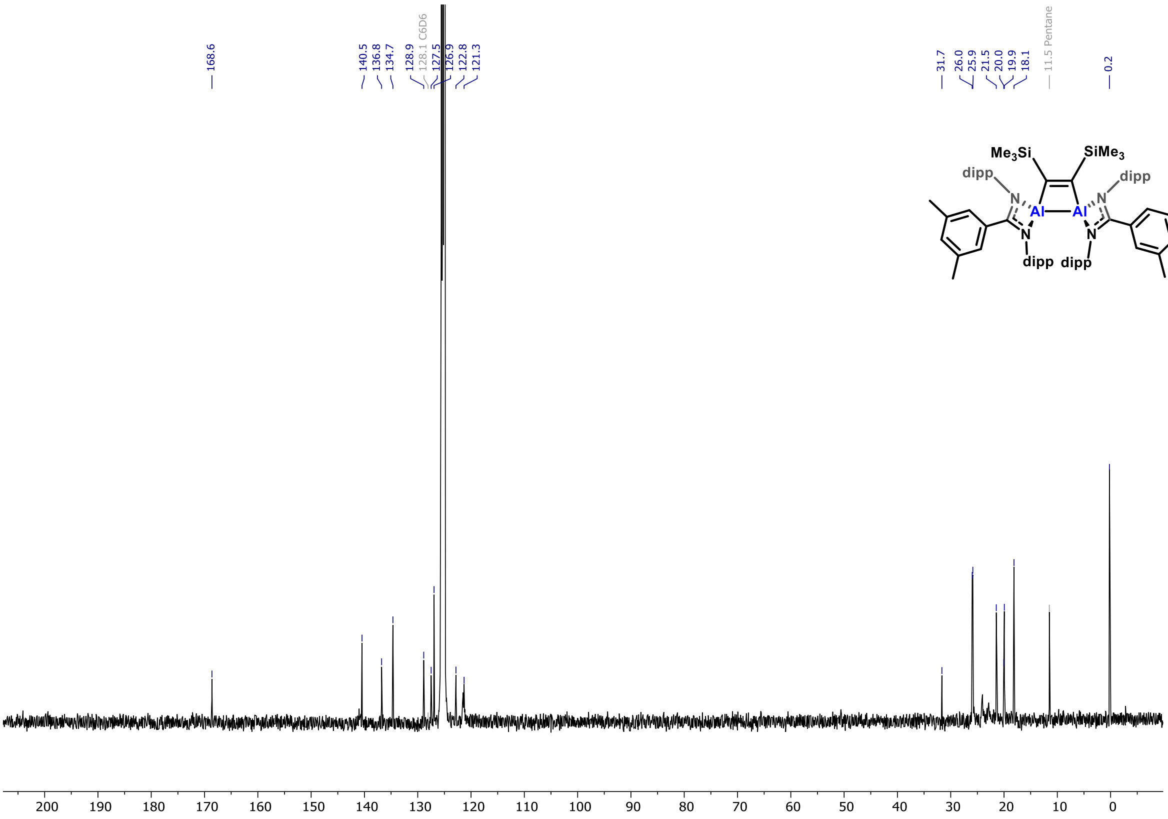

Figure S 90: $^{13}C\{^{1}H\}$ NMR (101 MHz, 298 K) spectrum of $\mathbf{6}^{m\text{-}xyl}$ in benzene-d_6

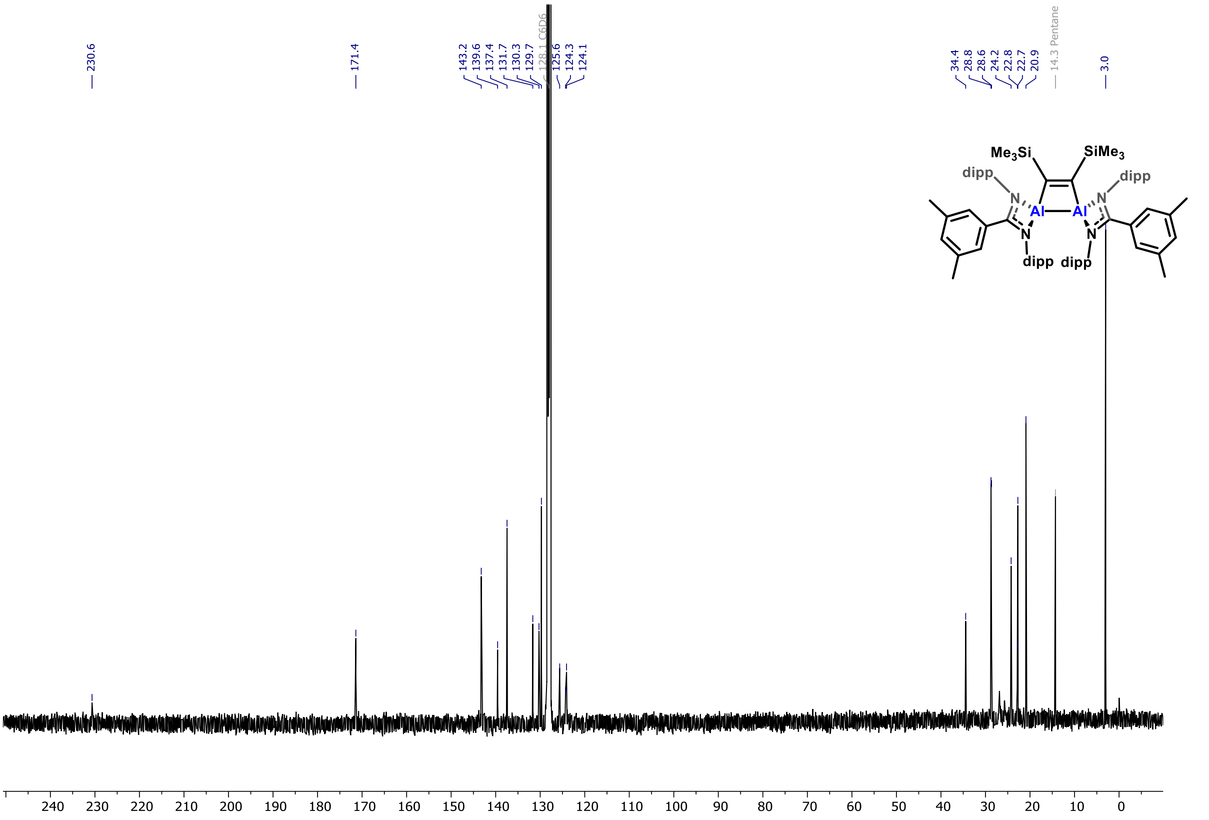

Figure S 91: wide $^{13}C\{^{1}H\}$ NMR (101 MHz, 298 K) spectrum of $\mathbf{6}^{m\text{-}xyl}$ in benzene-d_6

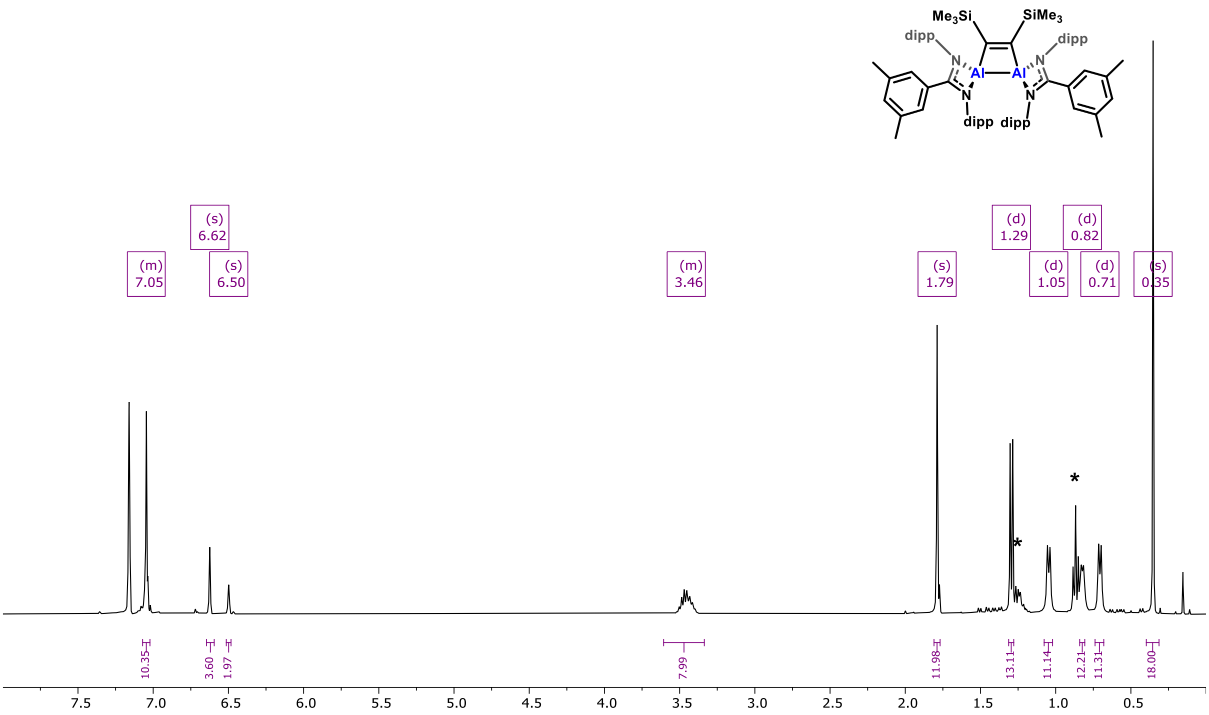

Figure S 92: ^{1}H NMR (400 MHz, 353 K) spectrum of $\mathbf{6}^{m\text{-}xyl}$ in benzene-d_6 (* residual pentane)

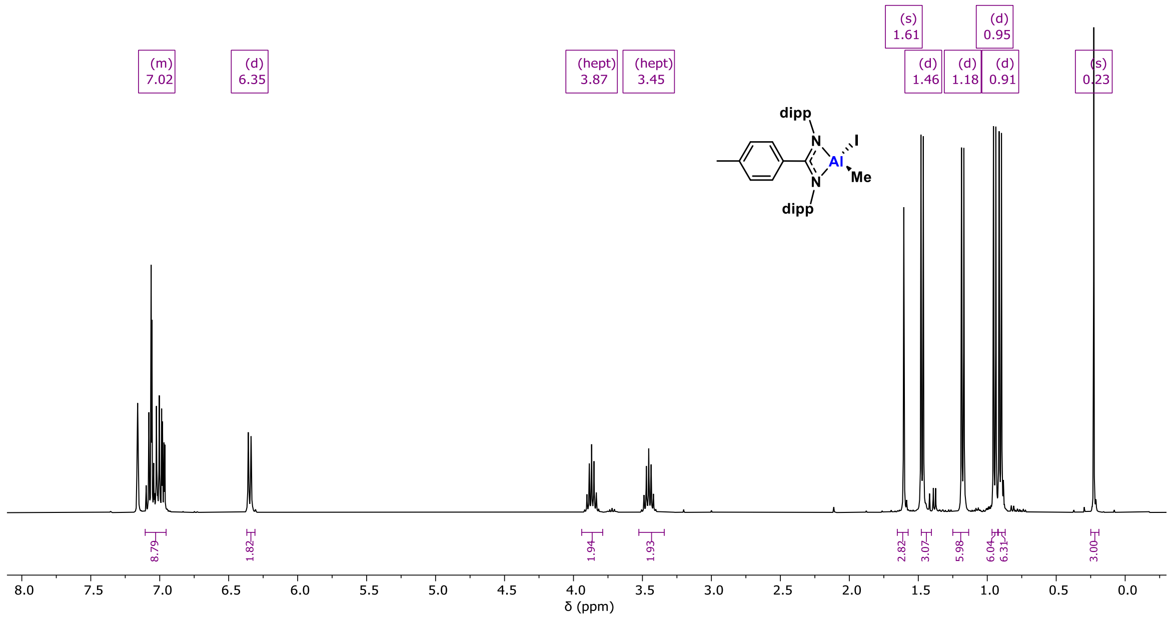

Figure S 93: ^{1}H NMR (400 MHz, 298 K) spectrum of ***7***$^{p\text{-}tol}$ in benzene-d_6

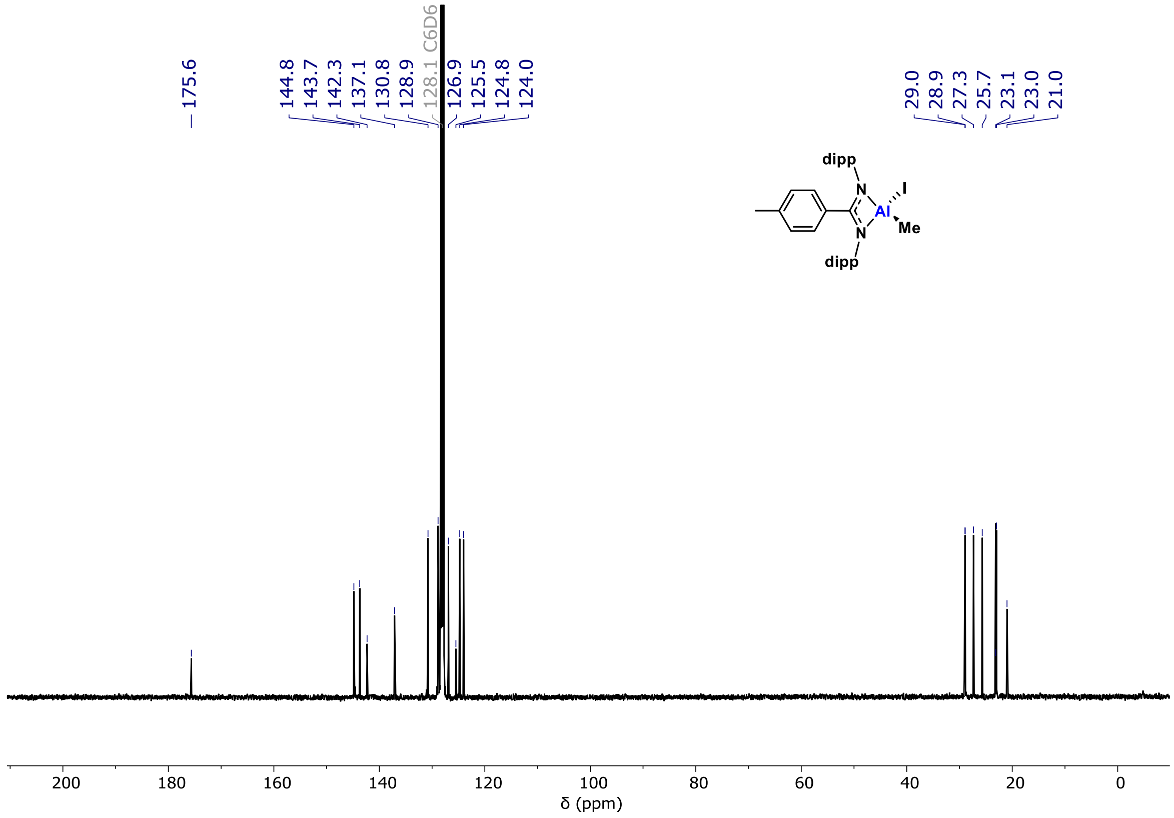

Figure S 94: $^{13}C\{^{1}H\}$ NMR (101 MHz, 298 K) spectrum of ***7***$^{p\text{-}tol}$ in benzene-d_6

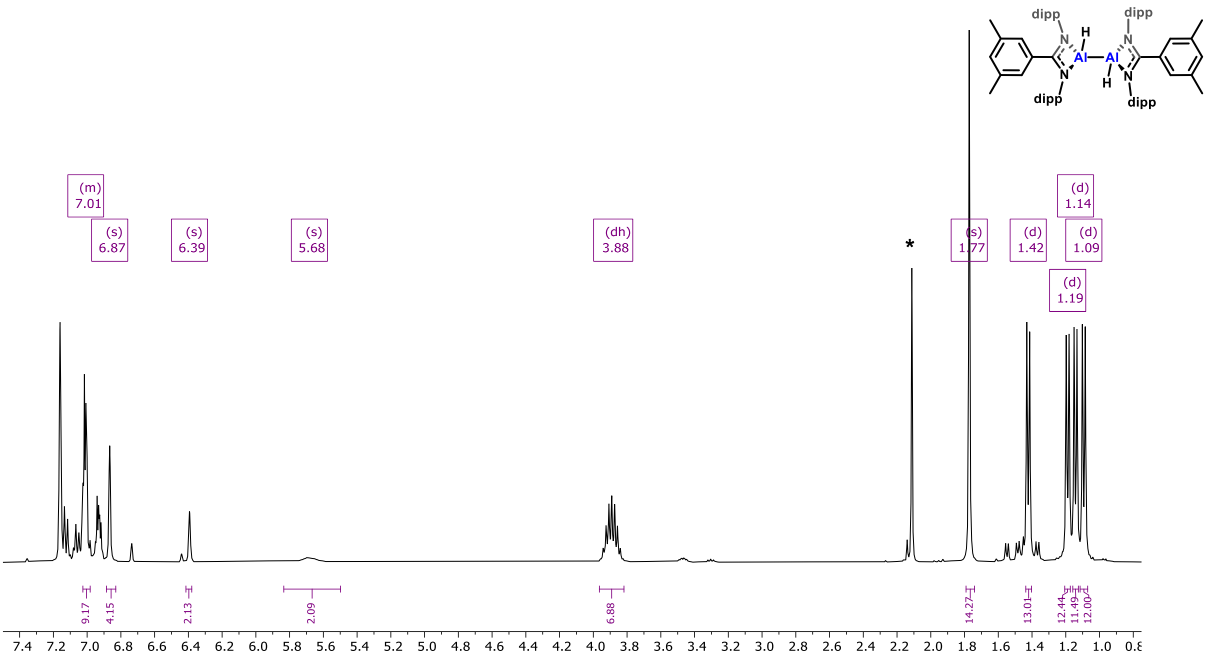

Figure S 95: ^{1}H NMR (400 MHz, 298 K) spectrum of $\boldsymbol{8^{m\text{-}xyl}}$ in benzene-d_6 (* residual toluene)

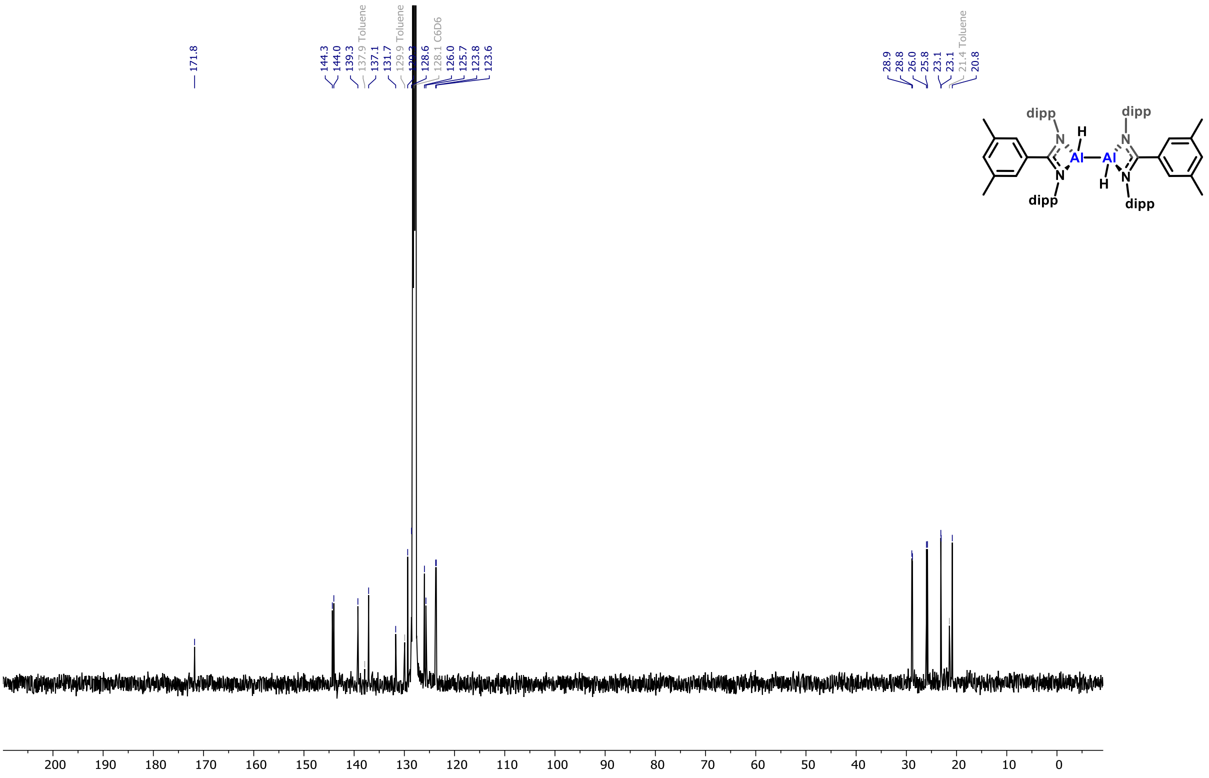

Figure S 96: $^{13}C\{^{1}H\}$ NMR (101 MHz, 298 K) spectrum of $\boldsymbol{8^{m\text{-}xyl}}$ in benzene-d_6

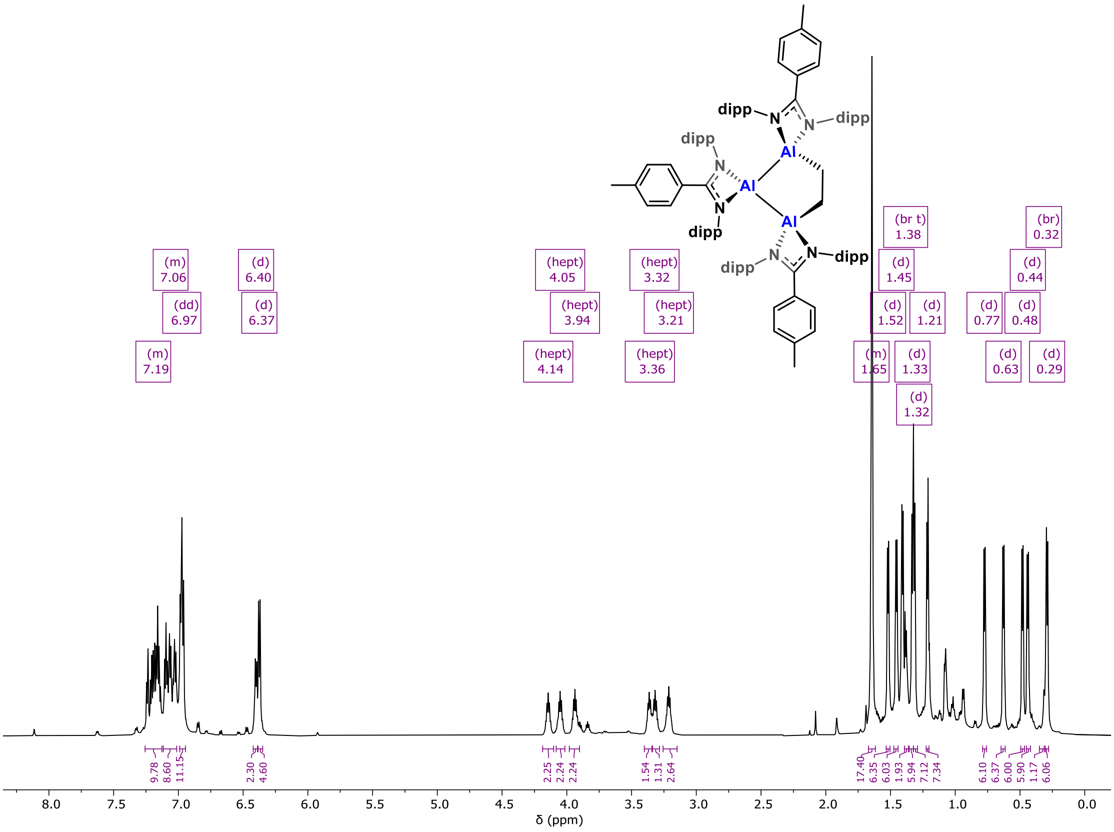

*Figure S 97: ^{1}H NMR (700 MHz, 298 K) spectrum of **9$^{p\text{-}tol}$** in benzene-d$_6$*

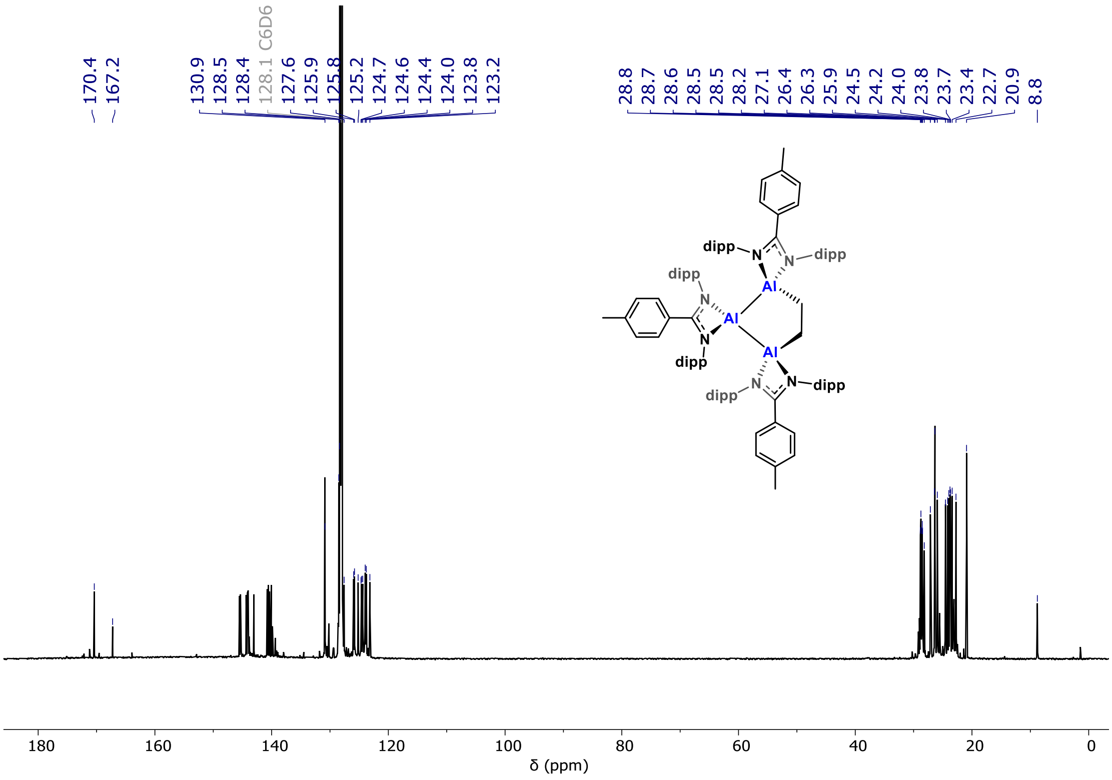

*Figure S 98: ^{13}C{^{1}H} NMR (176 MHz, 298 K) spectrum **9$^{p\text{-}tol}$** in benzene-d$_6$*

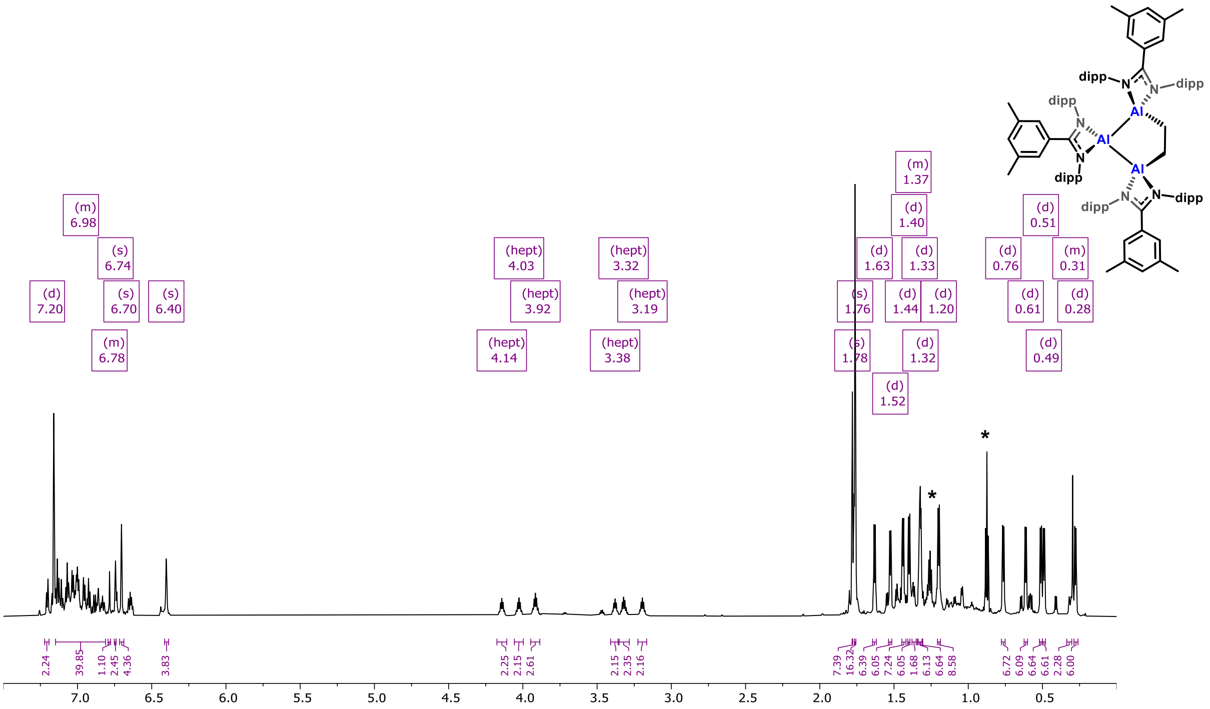

Figure S 99: ^{1}H NMR (800 MHz, 298 K) spectrum of **9**$^{m\text{-}xyl}$ in benzene-d$_6$ (* residual pentane)

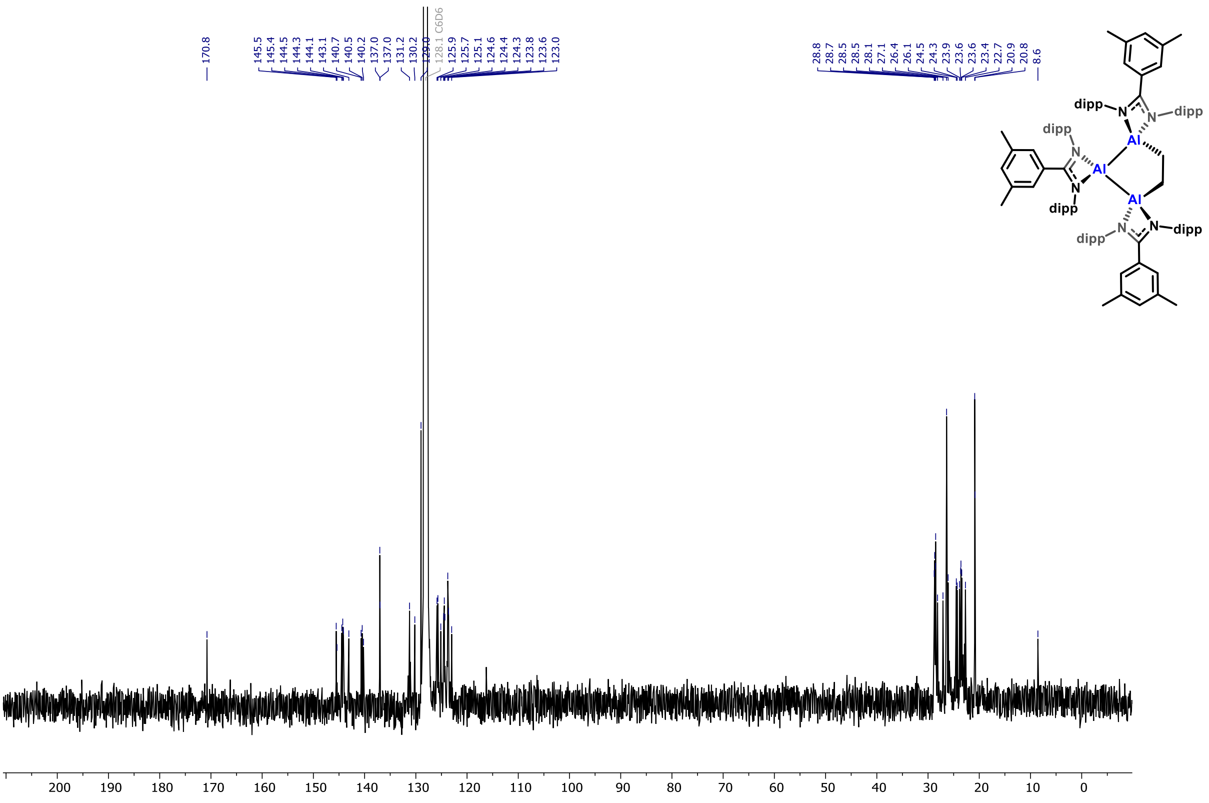

Figure S 100: ^{13}C{^{1}H} (201 MHz, 298 K) NMR spectrum of **9**$^{m\text{-}xyl}$ in benzene-d$_6$

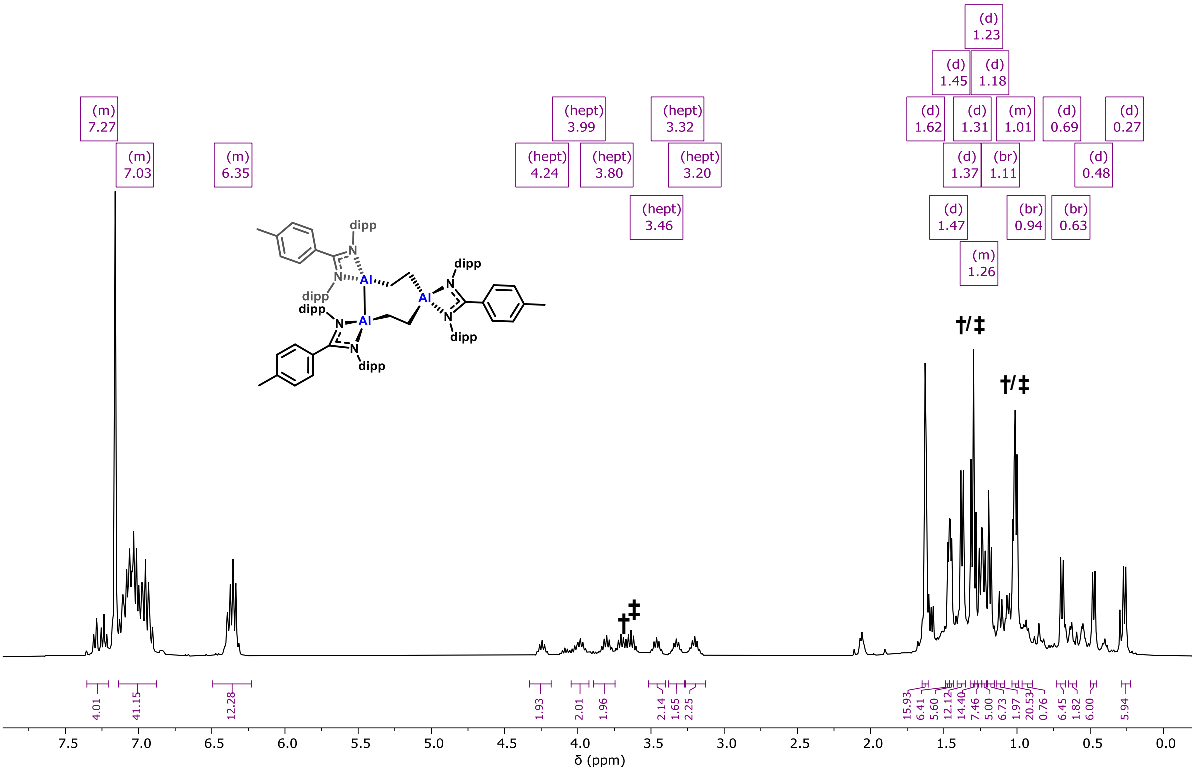

Figure S 101: ^{1}H NMR (400 MHz, 298 K) spectrum of **10$^{p\text{-}tol}$** in benzene-d_6 († **11$^{p\text{-}tol}$**, ‡ **12$^{p\text{-}tol}$**)

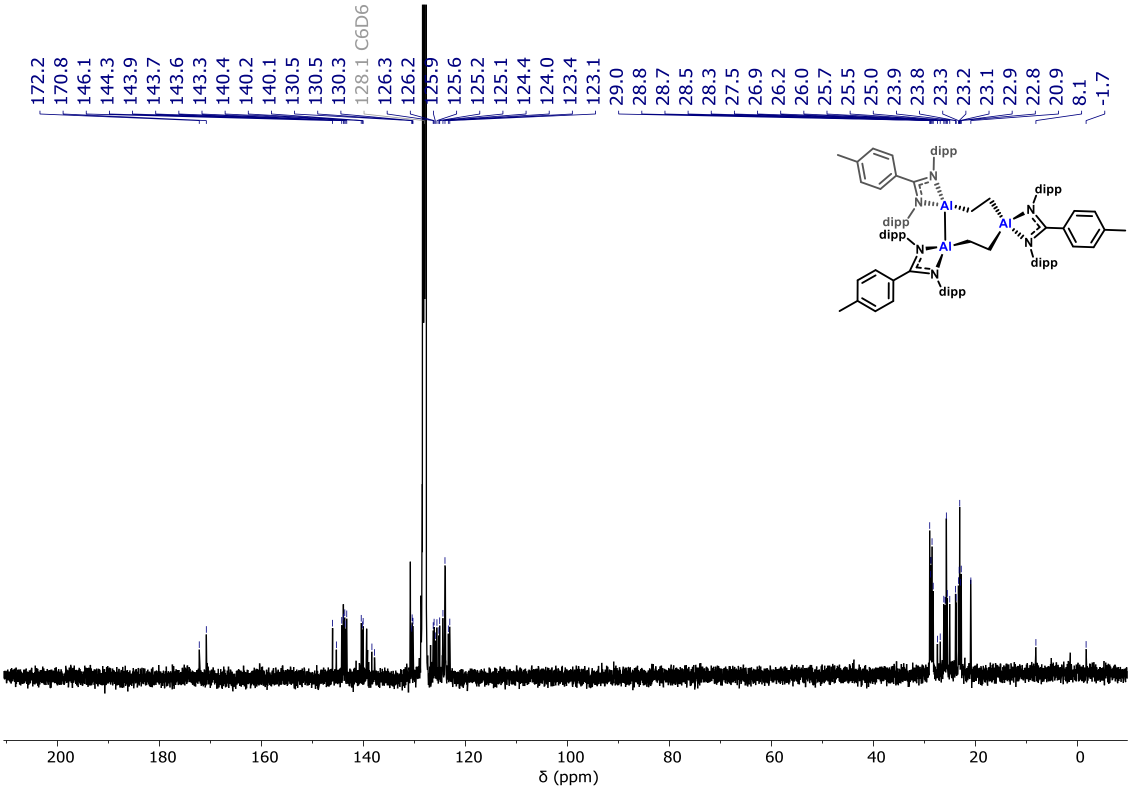

Figure S 102: ^{13}C{^{1}H} NMR (101 MHz, 298 K) spectrum of **10$^{p\text{-}tol}$** in benzene-d_6

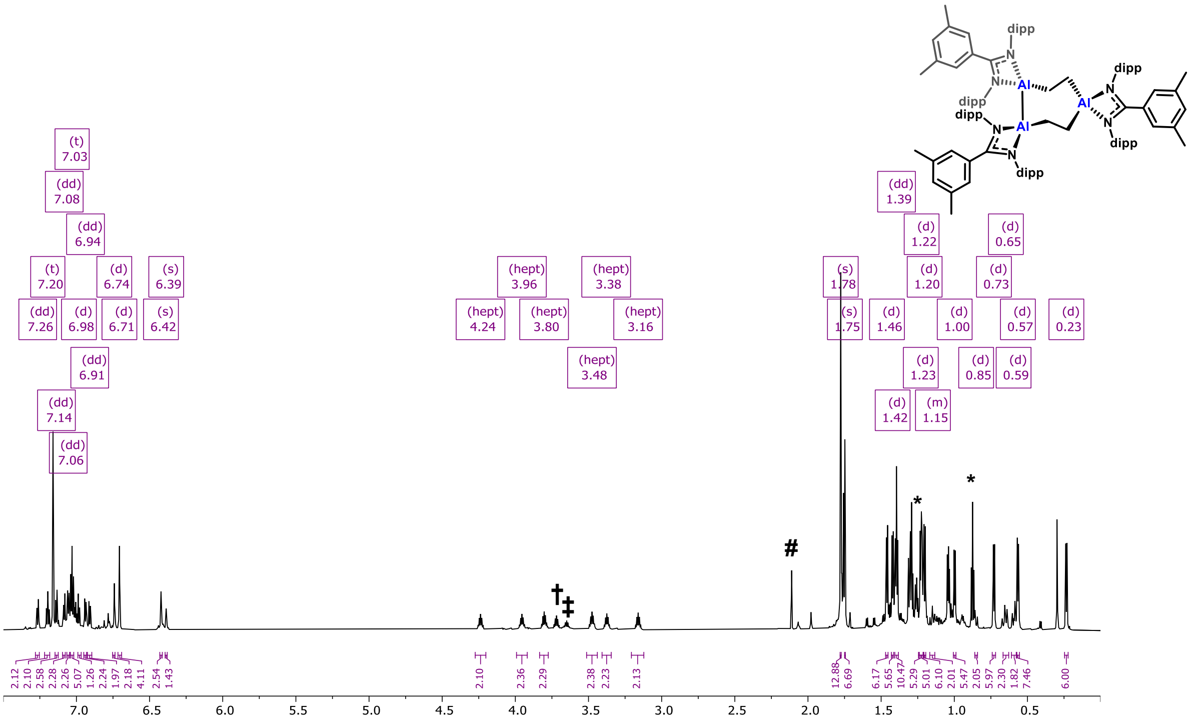

Figure S 103: ^{1}H (800 MHz, 298 K) NMR spectrum of $\mathbf{10}^{m\text{-}xyl}$ in benzene-d_6 (* residual pentane, # residual toluene, † $\mathbf{11}^{m\text{-}xyl}$, ‡ $\mathbf{12}^{m\text{-}xyl}$)

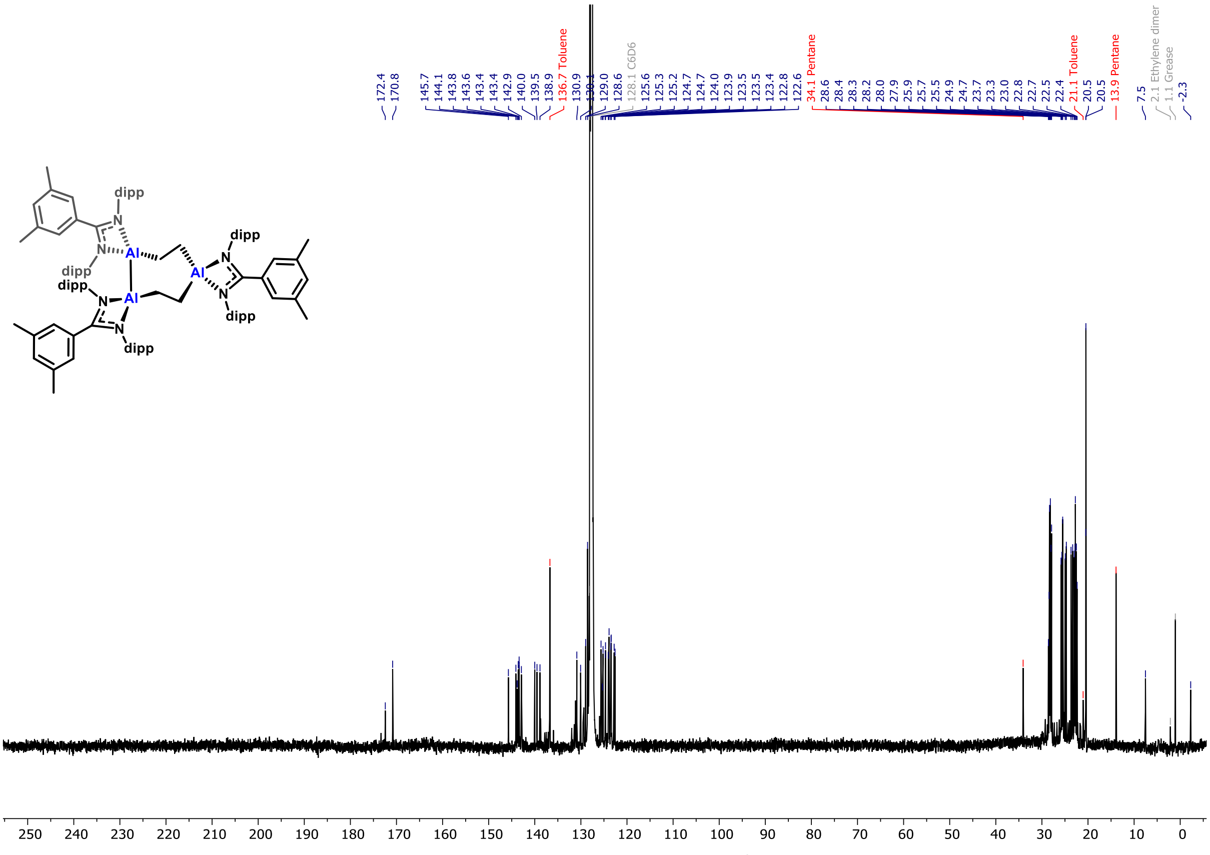

Figure S 104: $^{13}C\{^{1}H\}$ (201 MHz, 298 K) NMR spectrum of $\mathbf{10}^{m\text{-}xyl}$ in benzene-d_6

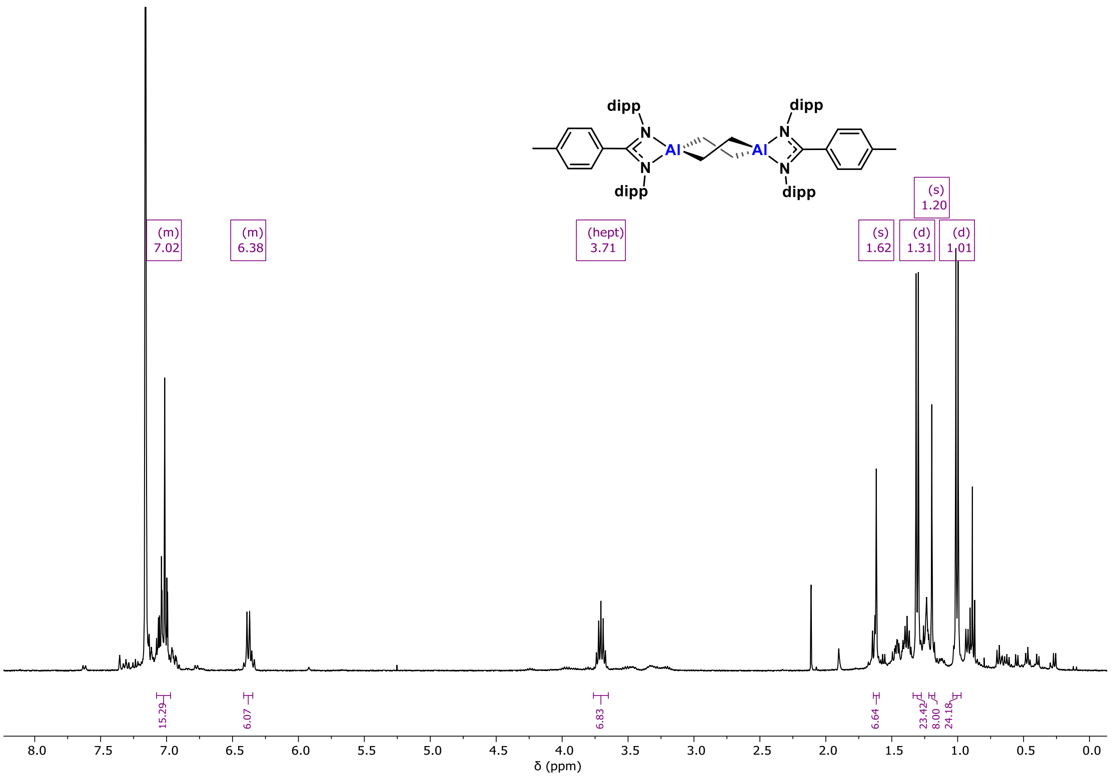

*Figure S 105: ^{1}H NMR (400 MHz, 298 K) spectrum of **11$^{p\text{-}tol}$** in benzene-d$_6$*

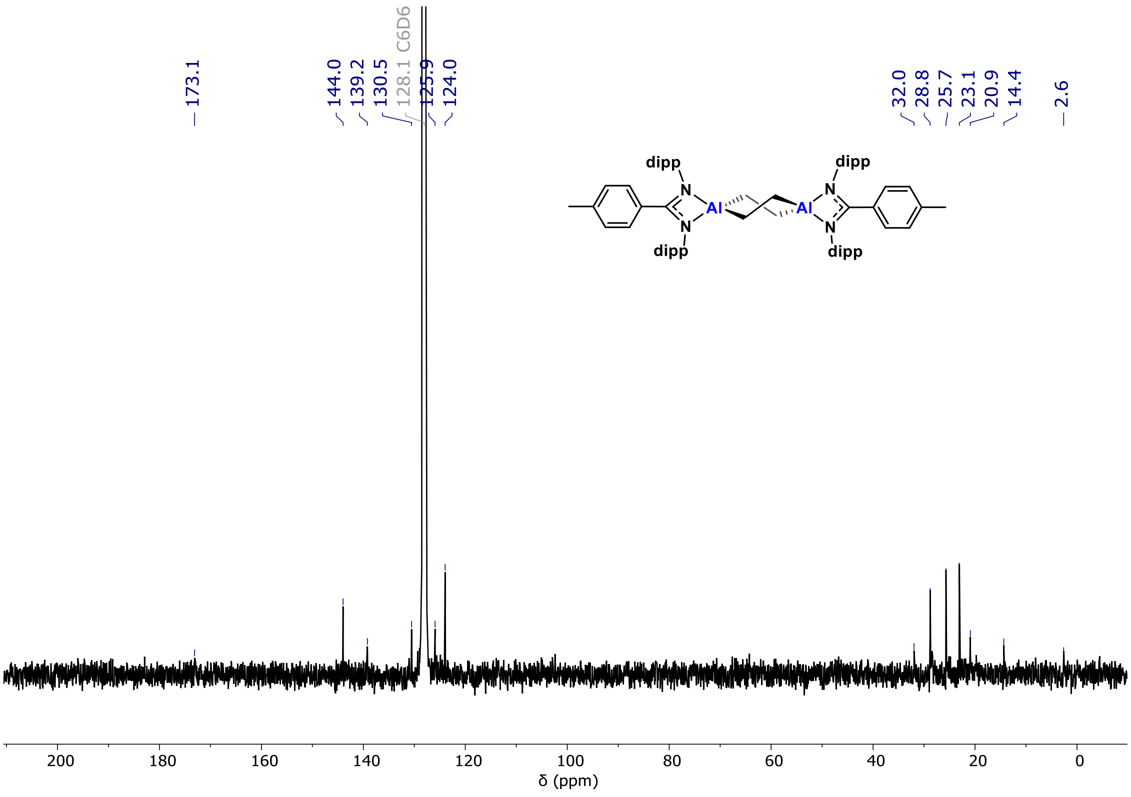

*Figure S 106: ^{13}C{^{1}H} NMR (101 MHz, 298 K) spectrum of **11$^{p\text{-}tol}$** in benzene-d$_6$*

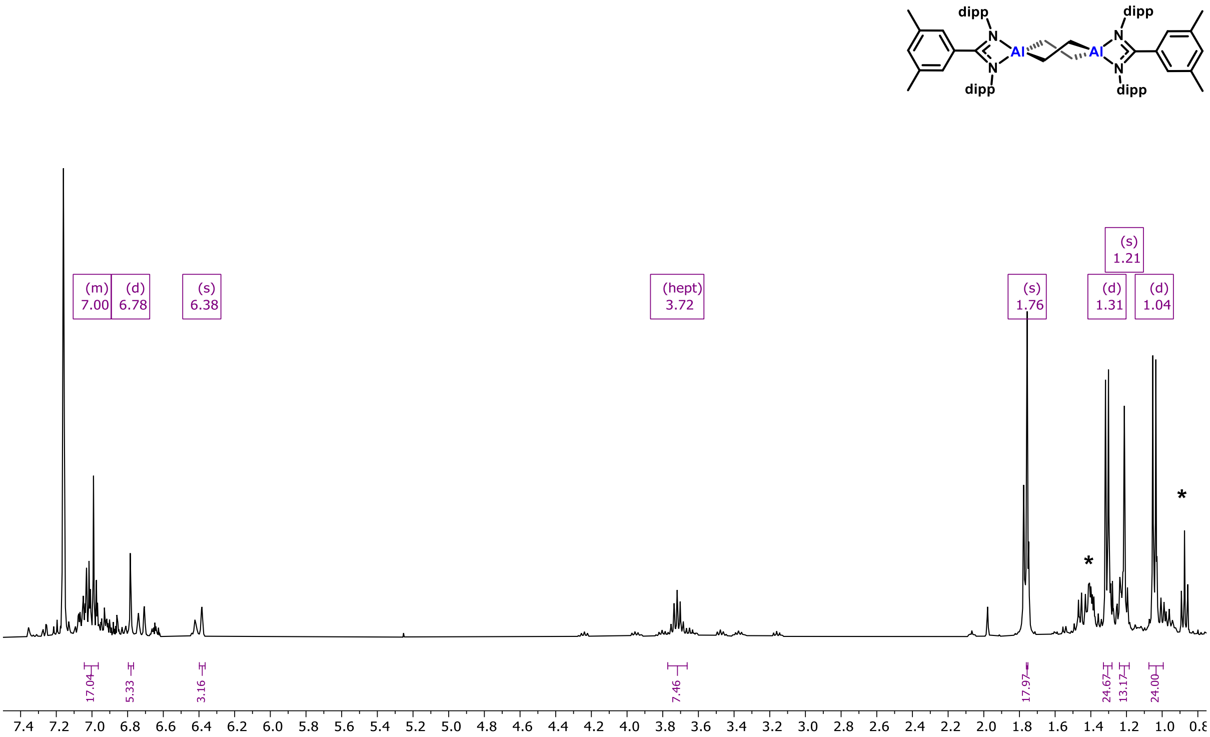

Figure S 107: ^{1}H NMR (400 MHz, 298 K) spectrum of **11**$^{m\text{-}xyl}$ in benzene-d$_6$ (* residual pentane, note substantial residual **10**$^{m\text{-}xyl}$)

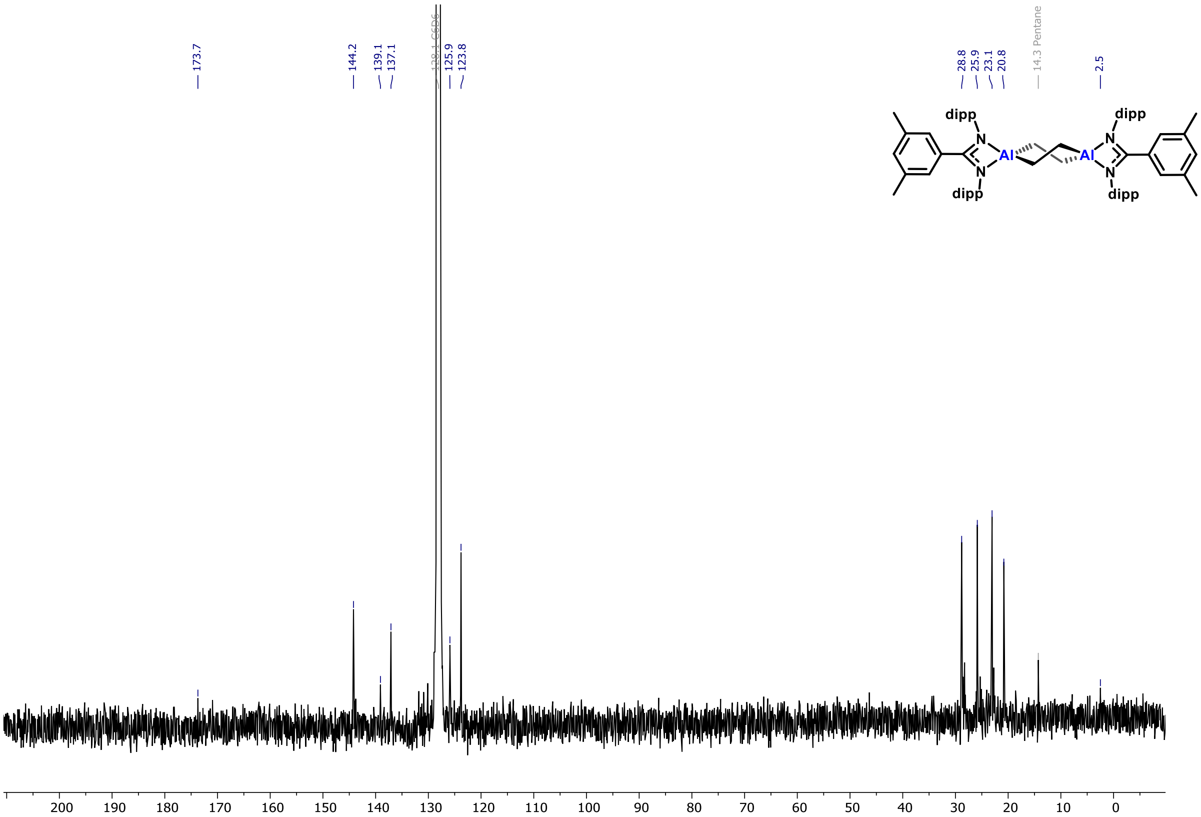

Figure S 108: ^{13}C{^{1}H} NMR (101 MHz, 298 K) spectrum of **11**$^{m\text{-}xyl}$ in benzene-d$_6$

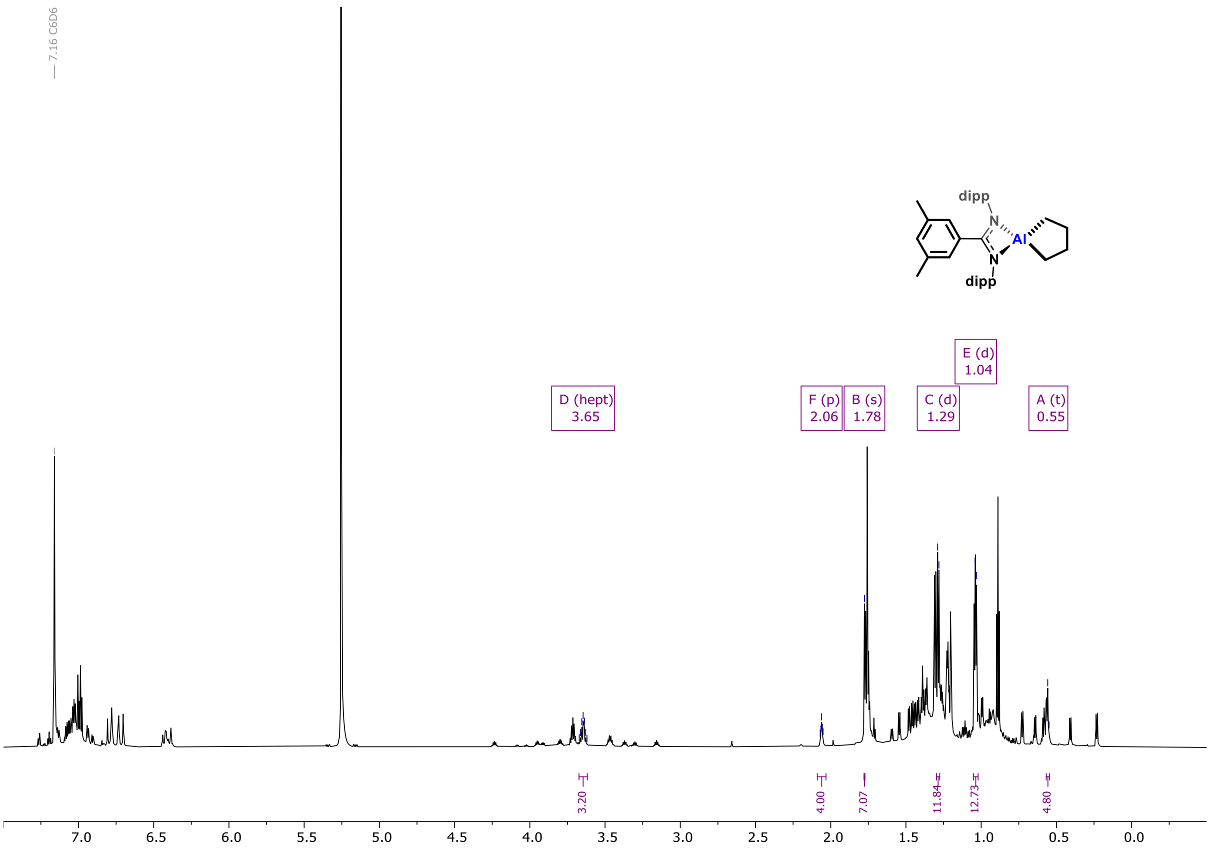

*Figure S 109: ^{1}H NMR (800 MHz, 298 K) spectrum of a mixture of **10**, **11** and **12**$^{m\text{-}xyl}$ in benzene-d$_6$, with the resonances for **12**$^{m\text{-}xyl}$ picked out, with ethene gas present*

7. References

1. Zhang, X. & Liu, L. L. A Free Aluminylene with Diverse σ-Donating and Doubly σ/π-Accepting Ligand Features for Transition Metals. *Angew. Chem. Int. Ed.* **60**, 27062–27069 (2021).
2. Boeré, R. T., Klassen, V. & Wolmershäuser, G. Synthesis of some very bulky N,N′-disubstituted amidines and initial studies of their coordination chemistry. *Dalton Trans.* 4147–4154 (1998).
3. Moxey, G. J. *et al.* Synthesis and characterisation of magnesium complexes containing sterically demanding N,N′-bis(aryl)amidinate ligands. *Dalton Trans.* **43**, 4838–4846 (2014).
4. Hobson, K., Carmalt, C. J. & Bakewell, C. Aluminum Amidinates: Insights into Alkyne Hydroboration. *Inorg. Chem.* **60**, 10958–10969 (2021).
5. Cui, C. *et al.* Synthesis and Structure of a Monomeric Aluminum(I) Compound [HC(CMeNAr)2Al] (Ar=2,6–iPr2C6H3): A Stable Aluminum Analogue of a Carbene. *Angew. Chem. Int. Ed.* **39**, 4274–4276 (2000).
6. Bonyhady, S. J. *et al.* Synthesis of a stable adduct of dialane(4) (Al2H4) via hydrogenation of a magnesium(I) dimer. *Nat. Chem.* **2**, 865–869 (2010).
7. Cherepanova, V. A., Gordeev, E. G. & Ananikov, V. P. Magnetic Stirring May Cause Irreproducible Results in Chemical Reactions. *JACS Au* **5**, 3789–3798 (2025).
8. Bakewell, C., Hobson, K. & Carmalt, C. J. Exploring Equilibria between Aluminium(I) and Aluminium(III): The Formation of Dihydroalanes, Masked Dialumenes and Aluminium(I) Species. *Angew. Chem. Int. Ed.* **61**, e202205901 (2022).
9. Squire, I., Tritto, M., Morell, J. & Bakewell, C. Probing the reactivity of a transient Al(I) species with substituted arenes. *Chem. Commun.* **60**, 12908–12911 (2024).
10. Boeré, R. T., Cole, M. L. & Junk, P. C. The syntheses and structures of some main group complexes of the sterically hindered N,N′-bis(2,6-diisopropylphenyl)-4-toluamidinate ligand. *New J. Chem.* **29**, 128–134 (2005).
11. Rigaku Oxford Diffraction, (2025), CrysAlisPro Software system, version 171.44.85, Rigaku Corporation, Wroclaw, Poland.
12. Sheldrick, G. M. SHELXT – Integrated space-group and crystal-structure determination. *Acta Crystallogr. A* **71**, 3–8 (2015).
13. Sheldrick, G. M. Crystal structure refinement with SHELXL. *Acta Crystallogr. C* **71**, 3–8 (2015).
14. Dolomanov, O. V., Bourhis, L. J., Gildea, R. J., Howard, J. A. K. & Puschmann, H. OLEX2: a complete structure solution, refinement and analysis program. *J. Appl. Cryst.* **42**, 339–341 (2009).
15. Neese, F. Software Update: The ORCA Program System—Version 6.0. *WIREs Computational Molecular Science* **15**, e70019 (2025).
16. King's College London e-Research team. King's Computational Research, Engineering and Technology Environment (CREATE). https://doi.org/10.18742/RNVF-M076 (2022) doi:10.18742/RNVF-M076.

17. Gaussian 16, Revision C.01, Frisch, M. J.; Trucks, G. W.; Schlegel, H. B.; Scuseria, G. E.; Robb, M. A.; Cheeseman, J. R.; Scalmani, G.; Barone, V.; Petersson, G. A.; Nakatsuji, H.; Li, X.; Caricato, M.; Marenich, A. V.; Bloino, J.; Janesko, B. G.; Gomperts, R.; Mennucci, B.; Hratchian, H. P.; Ortiz, J. V.; Izmaylov, A. F.; Sonnenberg, J. L.; Williams-Young, D.; Ding, F.; Lipparini, F.; Egidi, F.; Goings, J.; Peng, B.; Petrone, A.; Henderson, T.; Ranasinghe, D.; Zakrzewski, V. G.; Gao, J.; Rega, N.; Zheng, G.; Liang, W.; Hada, M.; Ehara, M.; Toyota, K.; Fukuda, R.; Hasegawa, J.; Ishida, M.; Nakajima, T.; Honda, Y.; Kitao, O.; Nakai, H.; Vreven, T.; Throssell, K.; Montgomery, J. A., Jr.; Peralta, J. E.; Ogliaro, F.; Bearpark, M. J.; Heyd, J. J.; Brothers, E. N.; Kudin, K. N.; Staroverov, V. N.; Keith, T. A.; Kobayashi, R.; Normand, J.; Raghavachari, K.; Rendell, A. P.; Burant, J. C.; Iyengar, S. S.; Tomasi, J.; Cossi, M.; Millam, J. M.; Klene, M.; Adamo, C.; Cammi, R.; Ochterski, J. W.; Martin, R. L.; Morokuma, K.; Farkas, O.; Foresman, J. B.; Fox, D. J. Gaussian, Inc., Wallingford CT, 2016.
18. Marenich, A. V., Cramer, C. J. & Truhlar, D. G. Universal Solvation Model Based on Solute Electron Density and on a Continuum Model of the Solvent Defined by the Bulk Dielectric Constant and Atomic Surface Tensions. *J. Phys. Chem. B* **113**, 6378–6396 (2009).
19. F. Weinhold, E. D. G., J, K. Badenhoop, A. E. Reed, J. E. Carpenter, J. A. Bohmann, C. M. Morales, P. Karafiloglou, C. R. Landis. NBO. Theoretical Chemistry Institute, University of Wisconsin, Madison (2018).
20. AIMAll (Version 19.10.12), Todd A. Keith, TK Gristmill Software, Overland Park KS, USA, 2019 (aim.tkgristmill.com).
21. Lu, T. & Chen, F. Multiwfn: A multifunctional wavefunction analyzer. *J. Comput. Chem.* **33**, 580–592 (2012).
22. Lu, T. A comprehensive electron wavefunction analysis toolbox for chemists, Multiwfn. *J. Chem. Phys.* **161**, 082503 (2024).
23. Knizia, G. Intrinsic Atomic Orbitals: An Unbiased Bridge between Quantum Theory and Chemical Concepts. *J. Chem. Theory Comput.* **9**, 4834–4843 (2013).
24. Stoychev, G. L., Auer, A. A. & Neese, F. Automatic Generation of Auxiliary Basis Sets. *J. Chem. Theory Comput.* **13**, 554–562 (2017).
25. Mitoraj, M. P., Michalak, A. & Ziegler, T. A Combined Charge and Energy Decomposition Scheme for Bond Analysis. *J. Chem. Theory Comput.* **5**, 962–975 (2009).
26. Sabando, R. C., Riplinger, C., Wennmohs, F., Neese, F. & Bistoni, G. Broadening the Scope of the ETS-NOCV Scheme: A Versatile Implementation in ORCA. *J. Chem. Theory Comput.* **21**, 7920–7934 (2025).
27. Chemcraft - graphical software for visualization of quantum chemistry computations. Version 1.8, build 682. https://www.chemcraftprog.com.
28. Pettersen, E. F. *et al.* UCSF Chimera--a visualization system for exploratory research and analysis. *J. Comput. Chem.* **25**, 1605–1612 (2004).
