# Supplementary material for: A neutral cyclic aluminium (I) trimer
Source: Nat Commun. 2026 Jan 30;17:1732. doi: 10.1038/s41467-026-68432-1 (PMC12913658; doi:10.1038/s41467-026-68432-1)
Supplement: Supplementary file 2 — Description of Additional Supplementary Files [file 41467_2026_68432_MOESM2_ESM.pdf]

Description of Additional Supplementary Files

File Name: Supplementary Data 1
Description: DFT coordinates text file
